# A dataset profiling the multiomic landscape of the prefrontal cortex in amyotrophic lateral sclerosis

**DOI:** 10.1093/gigascience/giae100

**Published:** 2024-12-18

**Authors:** Fabian Hausmann, Lucas Caldi Gomes, Sonja Hänzelmann, Robin Khatri, Sergio Oller, Marie Gebelin, Mojan Parvaz, Laura Tzeplaeff, Laura Pasetto, Qihui Zhou, Pavol Zelina, Dieter Edbauer, R Jeroen Pasterkamp, Hubert Rehrauer, Ralph Schlapbach, Christine Carapito, Valentina Bonetto, Stefan Bonn, Paul Lingor

**Affiliations:** Institute of Medical Systems Biology, Center for Biomedical AI (bAIome), Center for Molecular Neuroscience (ZMNH), University Medical Center Hamburg-Eppendorf, Hamburg 20251, Germany; Technical University of Munich, School of Medicine, rechts der Isar Hospital, Clinical Department of Neurology, Munich 81675, Germany; Institute of Medical Systems Biology, Center for Biomedical AI (bAIome), Center for Molecular Neuroscience (ZMNH), University Medical Center Hamburg-Eppendorf, Hamburg 20251, Germany; III Department of Medicine, University Medical Center Hamburg-Eppendorf, Hamburg 20251, Germany; Institute of Medical Systems Biology, Center for Biomedical AI (bAIome), Center for Molecular Neuroscience (ZMNH), University Medical Center Hamburg-Eppendorf, Hamburg 20251, Germany; Institute of Medical Systems Biology, Center for Biomedical AI (bAIome), Center for Molecular Neuroscience (ZMNH), University Medical Center Hamburg-Eppendorf, Hamburg 20251, Germany; Laboratoire de Spectrométrie de Masse Bio-Organique, Université de Strasbourg, Infrastructure Nationale de Protéomique, Strasbourg 67037, France; Technical University of Munich, School of Medicine, rechts der Isar Hospital, Clinical Department of Neurology, Munich 81675, Germany; Technical University of Munich, School of Medicine, rechts der Isar Hospital, Clinical Department of Neurology, Munich 81675, Germany; Research Center for ALS, Istituto di Ricerche Farmacologiche Mario Negri IRCCS, Milan 20156, Italy; German Center for Neurodegenerative Diseases (DZNE), Munich 81377, Germany; Munich Cluster for Systems Neurology (SyNergy), Munich 81377, Germany; Department of Translational Neuroscience, University Medical Center Utrecht, Utrecht University, Utrecht 3508, The Netherlands; German Center for Neurodegenerative Diseases (DZNE), Munich 81377, Germany; Munich Cluster for Systems Neurology (SyNergy), Munich 81377, Germany; Department of Translational Neuroscience, University Medical Center Utrecht, Utrecht University, Utrecht 3508, The Netherlands; Functional Genomics Center Zürich, ETH Zürich and University of Zürich, Zürich 8057, Switzerland; Functional Genomics Center Zürich, ETH Zürich and University of Zürich, Zürich 8057, Switzerland; Laboratoire de Spectrométrie de Masse Bio-Organique, Université de Strasbourg , Infrastructure Nationale de Protéomique, Strasbourg 67037, France; Research Center for ALS, Istituto di Ricerche Farmacologiche Mario Negri IRCCS, Milan 20156, Italy; Institute of Medical Systems Biology, Center for Biomedical AI (bAIome), Center for Molecular Neuroscience (ZMNH), University Medical Center Hamburg-Eppendorf, Hamburg 20251, Germany; Hamburg Center for Translational Immunology (HCTI), University Medical Center Hamburg-Eppendorf, Hamburg 20251, Germany; Technical University of Munich, School of Medicine, rechts der Isar Hospital, Clinical Department of Neurology, Munich 81675, Germany; German Center for Neurodegenerative Diseases (DZNE), Munich 81377, Germany; Munich Cluster for Systems Neurology (SyNergy), Munich 81377, Germany

**Keywords:** amyotrophic lateral sclerosis, multiomics analysis, neurodegeneration, prefrontal cortex, early disease mechanisms

## Abstract

Amyotrophic lateral sclerosis (ALS) is the most common motor neuron disease, which still lacks effective disease-modifying therapies. Similar to other neurodegenerative disorders, such as Alzheimer and Parkinson disease, ALS pathology is presumed to propagate over time, originating from the motor cortex and spreading to other cortical regions. Exploring early disease stages is crucial to understand the causative molecular changes underlying the pathology. For this, we sampled human postmortem prefrontal cortex (PFC) tissue from Brodmann area 6, an area that exhibits only moderate pathology at the time of death, and performed a multiomic analysis of 51 patients with sporadic ALS and 50 control subjects. To compare sporadic disease to genetic ALS, we additionally analyzed PFC tissue from 4 transgenic ALS mouse models (C9orf72-, SOD1-, TDP-43-, and FUS-ALS) using the same methods. This multiomic data resource includes transcriptome, small RNAome, and proteome data from female and male samples, aimed at elucidating early and sex-specific ALS mechanisms, biomarkers, and drug targets.

## Context

Amyotrophic lateral sclerosis (ALS) is a devastating motor neuron disease characterized by progressive paralysis and a shortened life span following symptom onset [1]. While the majority of ALS cases are sporadic (sALS) and lack a clear genetic predisposition, approximately 10% are associated with known genetic mutations (gALS) [2]. Among the most common genetic causes are mutations in the genes *C9orf72, SOD1, TARDBP*, and *FUS*. Interestingly, a subset of sALS patients also harbors disease-causing mutations [2–4]. Despite considerable research efforts, the exact etiology of sALS remains elusive, and effective disease-modifying treatments are currently unavailable [1, 5, 6]. Understanding the early mechanisms of ALS pathology is paramount for identifying diagnostic biomarkers and uncovering more effective therapeutic targets. Many investigations into ALS pathology have focused on end-stage disease by using postmortem central nervous system (CNS) tissue, which may obscure insights into earlier disease mechanisms that could offer more promising therapeutic avenues [7–9]. In contrast to the motor cortex, which is affected early in the disease and therefore often shows end-stage alterations at the death of the patients [7], the prefrontal cortex (PFC) is affected only later in the disease and thus presents a unique opportunity to explore earlier ALS pathology [10, 11]. Histological studies have revealed that while the motor cortex exhibits severe pathology in later stages of the disease [7–9], the PFC demonstrates intermediate TDP-43 pathology, suggesting its relevance in elucidating earlier disease-mediated alterations [10, 12]. A recent study employed multiomics to profile the molecular alterations in the spinal cord, another region heavily affected in ALS [13]. Other studies employed multiomic strategies in postmortem tissue from patients with ALS, but they included only a limited number of techniques, focusing on transcriptome- or genome-based technologies [14, 15]. Studies focusing on early alterations in ALS-affected brains in a comprehensive multiomic setting are still lacking [16].

In this context, the availability of omics datasets and robust analytical workflows is critical for advancing ALS research. Building upon our previous work [12], with this Data Note, we improved the accessibility of raw and processed data, alongside detailed descriptions of bioinformatics methodologies, as well as new data resources based on the original multiomic data. This includes extensive documentation of bioinformatics workflows and the provision of code to facilitate reproducibility and transparency in data analysis.

We provide a broad multiomic high-throughput sequencing data set of a cohort of 101 human samples from 4 different brain banks (*n* = 51 patients with sporadic ALS; *n* = 50 control subjects, males and females). The omic layers encompass mRNAomics, small RNAomics, and proteomics. Additionally, we provide corresponding data for 4 distinct ALS mouse models based on mutations in the genes *SOD1, C9orf72, FUS*, and *TARDBP*. Each mouse model includes both male and female samples, with transgenic and wild-type groups represented in each omic layer (cohort numbers balanced for sex and condition), ensuring a robust comparative analysis across species. Human PFC samples were provided by 4 different European brain banks (London Neurodegenerative Diseases Brain Bank, the Imperial College London—Multiple Sclerosis and Parkinson’s Tissue Bank, the Oxford Brain Bank, and the Netherlands Brain Bank). Subjects composing the control cohort did not present any signs of neurodegenerative diseases. Clinical features provided include age at death, postmortem interval (until the brains were sampled), disease onset, disease duration, and brain bank.

In brief, in our initial study [12], we identified distinct molecular subclusters within patients with ALS that showed varying patterns in gene, protein, and miRNA expression. This suggested the presence of different underlying disease mechanisms and underscored the need for personalized therapeutic approaches. Another important aspect of our study was the identification of pronounced sex differences captured in the molecular profiles of patients with ALS, with male patients exhibiting more pronounced alterations overall. Furthermore, our study emphasized and focused on the MAPK pathway as a critical therapeutic target. The involvement of this pathway suggests it could be a focal point for developing targeted treatments, which could improve the prognosis for patients with ALS. Other important pathways identified in our initial study were the activation of immune response, extracellular matrix composition, mitochondrial function, and RNA processing. The results from human analyses were corroborated in the selected ALS mouse models, which exhibited similar molecular patterns, and partially resembled the human subclusters revealed through the analysis of human brain tissue. The findings summarized here were validated across multiple datasets, reinforcing the significance of the identified molecular subclusters and pathways.

**Figure 1:**
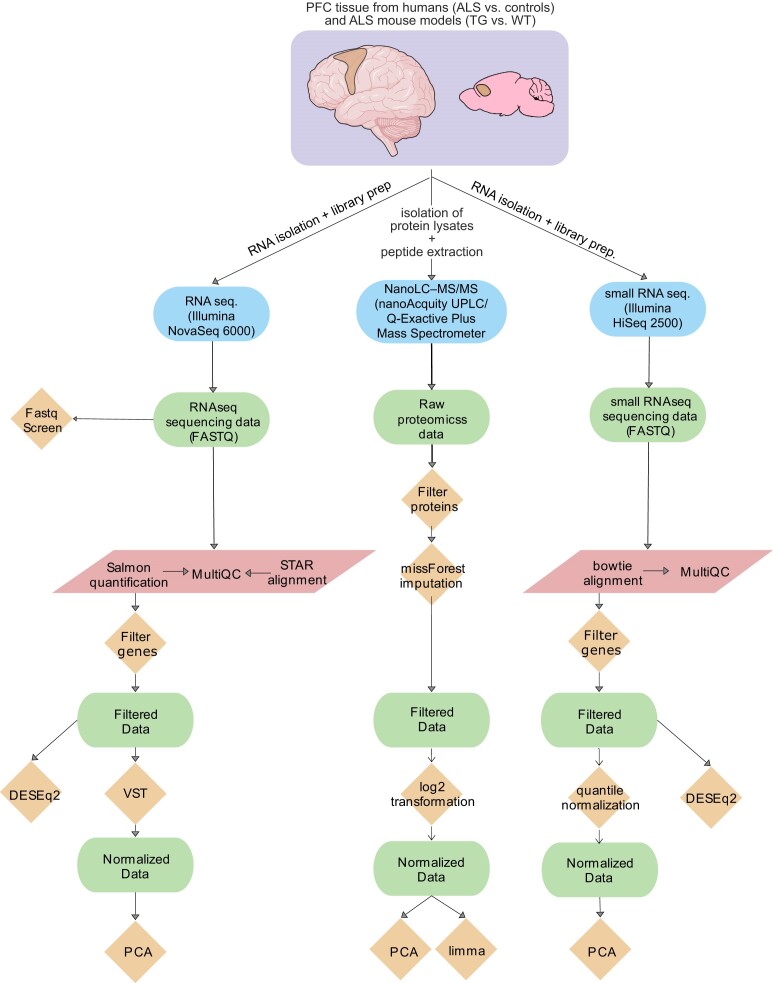
Overview of the bioinformatics workflow for RNA-seq, small RNA-seq, and proteomics data. Methods and processing scripts are shown in orange diamonds, high-throughput technologies depicted in blue rectangles with round edges, datasets available on disk in green rectangles with round edges, and Nextflow pipelines in red parallelograms. For the Nextflow pipelines, only a few steps are named here. The RNA-seq pipeline (v3.0) and the small RNA-seq smRNA-seq pipeline (v1.0) were used.

In summary, we describe here a findable, accessible, interoperable, and reproducible (FAIR) multiomics analysis workflow, including integration steps and accompanying data that are freely available. For this, we used standardized and versioned docker containers provided by Nextflow [17] for the preprocessing steps and documented the data-specific statistical and machine-learning analyses. An overview of this workflow can be found in Fig. 1.

## Methods

### Experimental data acquisition and preparation

#### Sample data description

This study investigates the molecular mechanisms underlying ALS using samples from human PFC and from 4 transgenic mouse models. The human cohort includes 51 patients with sALS and 50 control (CTR) subjects (Table 1). The ALS animal models include 4 genetically modified mouse strains: B6;129S6-Gt(ROSA)26Sortm1(TARDBP*M337V/Ypet)Tlbt/J mice (here simply referred to as TDP-43 mice) [18], B6SJL-Tg(SOD1*G93A)1Gur/J mice [19] (here referred to as SOD1 mice), (Poly)GA-NES/C9orf72(R26(CAG-Isl-175GA)-29×Nes-Cre) mice (here referred to as C9orf72 mice) [20], and Tg (Prnp-FUS)WT3Cshw/J mice (hereafter referred to as FUS mice) [21]. Each animal model cohort consists of 10 transgenic and 10 nontransgenic mice, balanced for sex (Table 2).

**Table 1:** Summary of the cohort numbers and demographics for the human cohort

Human cohort	Control	ALS
**Subjects**	50	51
**Age at death (in years)**	75 (43–94)	67 (44–83)
**Sex (F/M)**	28 F/22 M	16 F/35 M
**Disease duration (years)**	–	3 (1–28)
Unprecise/unknown	–	39.2%
**Brain bank origin**		
NBB	18.0%	17.6%
Oxford BB	20.0%	27.5%
ICL MS and PD TB	38.0%	0.0%
London NDBB	24.0%	54.9%

ALS: amyotrophic lateral sclerosis; ICL MS and PD TB: Imperial College London—Multiple Sclerosis and Parkinson’s Tissue Bank; London NDBB: London Neurodegenerative Diseases Brain Bank; NBB: The Netherlands Brain Bank; OBB: Oxford Brain Bank. Sex: male = M; female = F. A full description of the clinical features for the human cohort can be accessed within the supplementary data from our main publication [12].

**Table 2:** Summary of the cohort numbers for the ALS mouse models

Mouse cohorts	TDP-43	
**Condition (WT/TG)**	10 WT	10 TG
**Sex (F/M)**	5 F/5 M	5 F/5 M
	**SOD1**	
**Condition (WT/TG)**	11 WT	9 TG
**Sex (F/M)**	5 F/6 M	5 F/4 M
	**C9orf72**	
**Condition (WT/TG)**	10 WT	10 TG
**Sex (F/M)**	6 F/4 M	6 F/4 M
	**FUS**	
**Condition (WT/TG)**	10 WT	10 TG
**Sex (F/M)**	5 F/5 M	5 F/5 M

Condition: wild type = WT; transgenic = TG. Sex: male = M; female = F.

#### Sample acquisition methods

Data collection and handling are reported in Caldi Gomes et al. [12]. All experimental data presented here comply with the relevant ethical regulations. Consent for the donation of brain material for the subjects who compose the human cohorts was handled individually by the brain banks involved in this study. Ethical approval was obtained from the Ethics Committees of the University Medical Center Göttingen (2/8/18 AN) and the Technical University Munich (145/19 S-SR). All animal experiments complied with international and local animal welfare laws and were approved by the respective regulatory organs for each involved research center. Experiments with transgenic SOD1 and FUS mice were prospectively approved by the Mario Negri Institutional Animal Care and Use Committee and the Italian Ministry of Health (Prot. No. 9F5F5.143/Prot. No. 9F5F5.250). Experiments with C9orf72 transgenic mice followed the regulations from the German Animal Welfare Act (Tierschutzgesetz/Tierschutz-Versuchstierverordnung, Regierungsbezirke Oberbayern, Prot. No. TV 55.2–2532.Vet_02–17-106). Experiments with TDP43 transgenic animals were approved by the Centrale Commissie Dierproeven (CCD) of Utrecht University (CCD license: AVD 1150020171565), in accordance with Dutch animal welfare laws (Wet op de Dierproeven 2014) and European regulations (guideline 2010/63/EU).

### Human postmortem PFC samples

Human PFC samples were sourced from 4 brain banks: the Netherlands Brain Bank, London Neurodegenerative Diseases Brain Bank, Imperial College London Multiple Sclerosis and Parkinson’s Tissue Bank, and the Oxford Brain Bank. Samples were shipped on dry ice and stored at −80°C upon arrival at the rechts der Isar Hospital Department of Neurology, Technical University of Munich. PFC samples were sectioned using a cryostat at −20°C and processed to collect approximately 20 mg of tissue per sample, which was stored at −80°C until further use.

### ALS animal models

Four transgenic mouse models were used to represent the most frequent ALS-causing genes. The mice were kept in pathogen-free facilities with a 12-hour light/dark cycle and unrestricted access to food and water. Each mouse model was euthanized at specific presymptomatic or early symptomatic stages for biomaterial collection. Euthanization times were TDP-43 (26 weeks), SOD1 (14 weeks), C9orf72 (4.5 weeks), and FUS (4 weeks). Mice were perfused with ice-cold phosphate-buffered saline (PBS) before microdissection. The prefrontal cortex was isolated, transferred to nuclease-free tubes, and stored at −80°C until RNA and protein isolation. For each model, a total of 20 transgenic and control (wild-type) mice were selected and balanced for condition/sex (TDP-43: 5 females and 5 males for transgenic and control cohorts; SOD1: 5 females and 6 males for the control cohort, 5 females and 4 males for the transgenic cohort; C9orf72: 6 females and 4 males for the control cohort, 4 females and 6 males for the transgenic cohort; FUS: 5 females and 5 males for transgenic and control cohorts).

### RNA isolation

Total RNA from human and animal PFC samples was isolated using TRIzol Reagent. RNA was precipitated, washed with ethanol, reconstituted in nuclease-free water, and treated with DNase to remove DNA contamination. Nucleic acid concentration and purity were assessed using a NanoDrop One spectrophotometer and an Agilent 6000 NanoKit for RNA integrity.

### RNA sequencing

mRNA and small RNA sequencing (RNA-seq) experiments were conducted as single end at the Functional Genomics Center Zürich. For mRNA sequencing, the TruSeq Stranded mRNA Kit and the SMARTer Stranded Total RNA-Seq Kit v2 Pico Input Mammalian were used. The RealSeq-AC miRNA was used for small RNA-seq experiments. After library preparation, normalization was done using Tris–Cl (pH 8.5) containing 0.1% Tween 20 (at 10 nM for the TruSeeq kit, 5 nM for the SMARTer Stranded kit, and 2 nM for the RealSeq-AC miRNA kit). Sequencing was performed in the Illumina NovaSeq 6000 platform (for RNA-seq) and the HiSeq 2500 platform (for small RNA-seq).

### Proteomics

Proteins from human and mouse PFC tissue samples were extracted with a biosmasher using 350 µL MeOH:H_2_O (4:1), resuspended in 200 µL Laemmli buffer (10% sodium dodecyl sulfate [SDS], Tris 1 M, pH 6.8, glycerol) and then centrifuged at 11.135 rpm at 4°C for 5 minutes. Then, 100 µg of protein lysate was denatured by heating at 95°C for 5 minutes and stacked in an in-house prepared 5% acrylamide SDS–polyacrylamide gel electrophoresis (PAGE) stacking gel. Gel bands were reduced and alkylated. Digestion was performed overnight at 37°C using modified porcine trypsin (Mass Spec Grade, Promega; enzyme/protein ratio of 1:80). The peptides were extracted by sequential application of 60% acetonitrile and 100% acetonitrile (ACN). Peptides were resuspended in 30 µL H_2_O, 2% ACN, and 0.1% FA, and iRT peptides (Biognosys) were added according to the manufacturer’s instructions. The generated samples were analyzed using nanoLC-MS/MS (nanoAcquity UltraPerformance LC; Waters), coupled to a Q-Exactive Plus Mass Spectrometer (Thermo Fisher Scientific). Data were further processed using MaxQuant [22].

In addition, an open modification search was performed. MGF files from the mouse and human proteomics data were loaded into IonBot [23] software (v. 0.11.0). Provided databases were used, either human (9,606 entries) or *Mus musculus* (10,090 entries), with a K|R cleavage pattern. Error tolerances were set on default values: mass spectrometry (MS) precursor tolerance at 20 ppm and MS/MS fragment tolerance at 0.02 Da. Methionine oxidation and protein N-term acetylation were set as variable modifications, while cysteine carbamidomethylation was set as a fixed modification. Open modification search option was enabled.

### Data Preparation

#### mRNA-seq and small RNA-seq data processing

**Figure 2:**
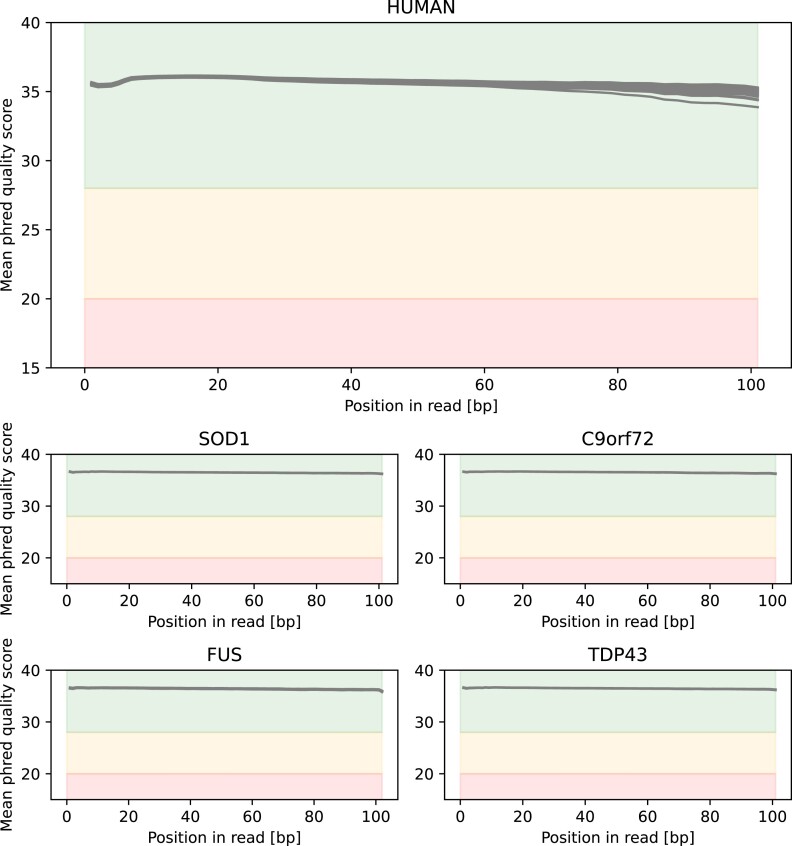
Demonstration of overall quality of reads on the transcriptome level. Mean phred quality scores, as reported by FastQC of the RNA-seq data, are displayed. Regions are colored according to FastQC’s quality definitions (greed: good, orange: okay, red: bad). Overall all reads show a good quality.

**Figure 3:**
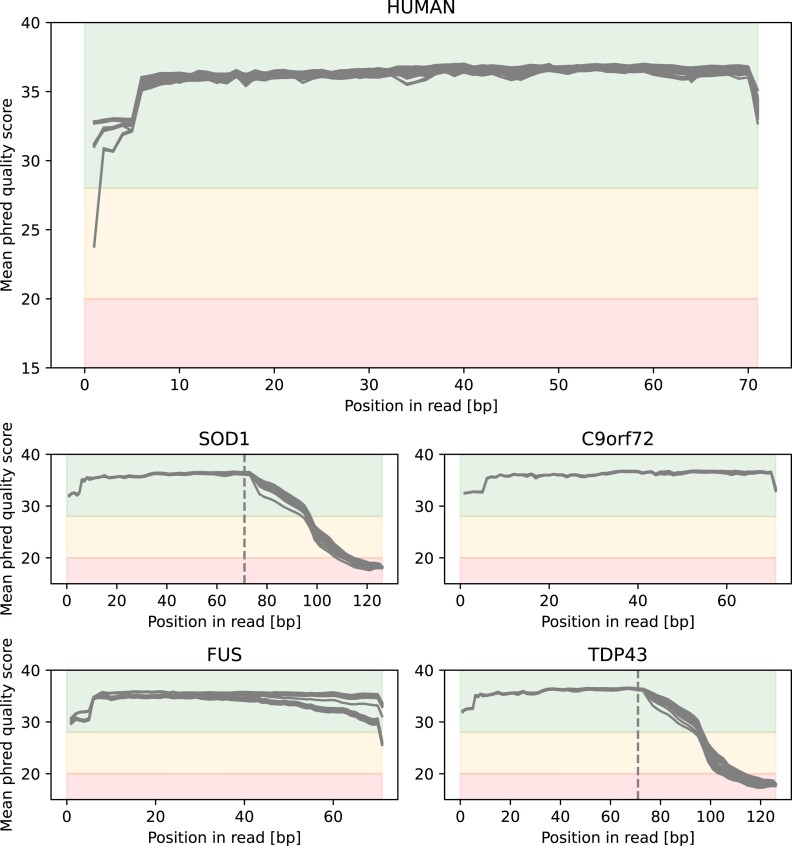
Demonstration of overall quality of reads on small RNA data level. Mean phred quality scores as reported by FastQC are displayed. Regions are colored according to FastQC’s quality definitions (greed: good, orange: okay, red: bad). For SOD1 and TDP43, the expected length after trimming is indicated by the gray-dotted line; for the others, only the expected length of reads was provided. Overall all reads show a good quality.

RNA-seq data were processed using the Nextflow [17] Core RNA-seq pipeline version 3.0 with the following parameters: –igenomes_ignore true –fasta <version>.genome.fa.gz –gtf gencode.<version>.annotation.gtf.gz –pseudo_aligner salmon –gencode –deseq2_vst. Quality checks were conducted with FastQC [24] (Fig. 2), and preprocessing steps included adapter trimming and quality filtering to remove low-quality reads and artifacts. Salmon [25] was used for pseudo-alignment and quantitation, with indices built from GENCODE [26] annotations GRCm39 for mouse and GRCh38 for human. Small RNA-seq data were processed using the Nextflow [17] Core smRNA-seq pipeline version 1.0 with the parameters –genome <genome> –mirna_gtf mirbase_<species>.gff3. FastQC [24] (Fig. 3) and miRTrace [27] were used for quality checks, followed by adapter trimming and quality filtering. Alignment was performed with Bowtie [28], and feature counting utilized samtools [29] using miRBase [30] annotations (version 22.1).

#### Filtering and transformation

For RNA-seq and small RNA-seq, count matrices were filtered to retain features with at least 10 counts in 50% of samples for any condition or sex. For the small RNA-seq data, normalization was performed using quantile normalization implemented in the preprocessCore [31] R package. For RNA-seq, variance-stabilizing transformation (VST) implemented in DESeq2 [32] was used for normalization, ensuring consistent and comparable expression values across samples. The total number of detected genes and small RNAs (sRNAs) for each dataset can be found in Table 3.

**Table 3:** Number of mapped entities for human and mouse data

	Transcriptomics	Proteomics	miRNA (mature/hairpin)
**Human**	19,641	2,363	736 (224/512)
**SOD1–Mouse**	16,583	2,854	893 (526/367)
**TDP43–Mouse**	16,801	2,802	907 (534/373)
**C9orf72–Mouse**	17,465	2,866	754 (271/483)
**FUS–Mouse**	17,230	2,522	812 (468/344)

#### Proteomics

Proteomics data were processed with MaxQuant [22] software. Protein peaks were assigned using trypsin/P specificity against an in-house–generated protein sequence database containing mouse entries from UniProtKB-SwissProt. The “match between runs” option facilitated protein quantification. Only Swiss-Prot proteins were retained, and low-abundance proteins detected in less than 50% of samples were filtered out. Missing values were imputed using the missForest [33] algorithm and intensities were log_2_-transformed for variance stabilization. A maximum false discovery rate (FDR) of 1% was applied at both peptide and protein levels. The total number of detected proteins for each dataset can be found in Table 3.

### Higher-level data analysis and machine learning

#### Differential expression and enrichment analyses

Downstream analyses of the RNA-seq and small RNA-seq data included differential expression analysis using DESeq2 [32] to identify differentially expressed genes/miRNAs between experimental conditions. Principal component analysis (PCA) was used for dimensionality reduction and visualization of sample relationships using VST-normalized RNA-seq data and quantile-normalized small RNA-seq data.

#### Proteomics data analysis

Linear modeling for differential abundance analysis was performed using the limma [34] package, with *P* values adjusted for multiple testing using the Benjamini–Hochberg correction. PCA was used for visualization.

#### Bioinformatics workflow

To allow for reproducible and interpretable bioinformatics workflow, we will describe the construction of our workflow here. Our workflow consists of multiple stages, equivalent to single scripts executed for 1 or multiple datasets with multiple parameters. We used Data Version Control (DVC) as a workflow management tool, because it allows the use of any script as stages in our computational workflow, automatically takes care of dependencies between these stages, and executes only stages that changed compared to the last execution. Furthermore, it provides the option to share raw and processed data between multiple users. The executed code for each stage is provided as script files written in bash, R, and Python. The execution of the scripts, their outputs, and their dependencies are defined in dvc.yaml files, with all important parameters found in params.yaml files. All scripts can be automatically executed using DVC. Since the execution of scripts depends on the package versions used, in R as well as in Python, proper maintenance of package versions is important.

Therefore, we containerized all applications by providing a docker image. In other instances, we used readily available docker images. This allows the automatic execution of our workflow using docker, if available, and prevents users from struggling to install the correct package versions. Furthermore, we value community efforts in providing reproducible workflows for the analysis of RNA-seq and small RNA-seq data implemented in Nextflow. These pipelines were integrated into our workflow as well, allowing us to easily adapt to recent developments in the workflow without much user effort.

All outputs of our workflow will be structured by mouse model and can be used for other applications, integrated into the workflow or not. However, we recommend integrating further analysis into the DVC workflow, as not to break the reproducibility principle of the extended workflow. In this case, also newly added scripts will automatically be executed if the underlying data or scripts are changing in any way.

## Data Validation and Quality Control

### RNA-seq data

**Figure 4:**
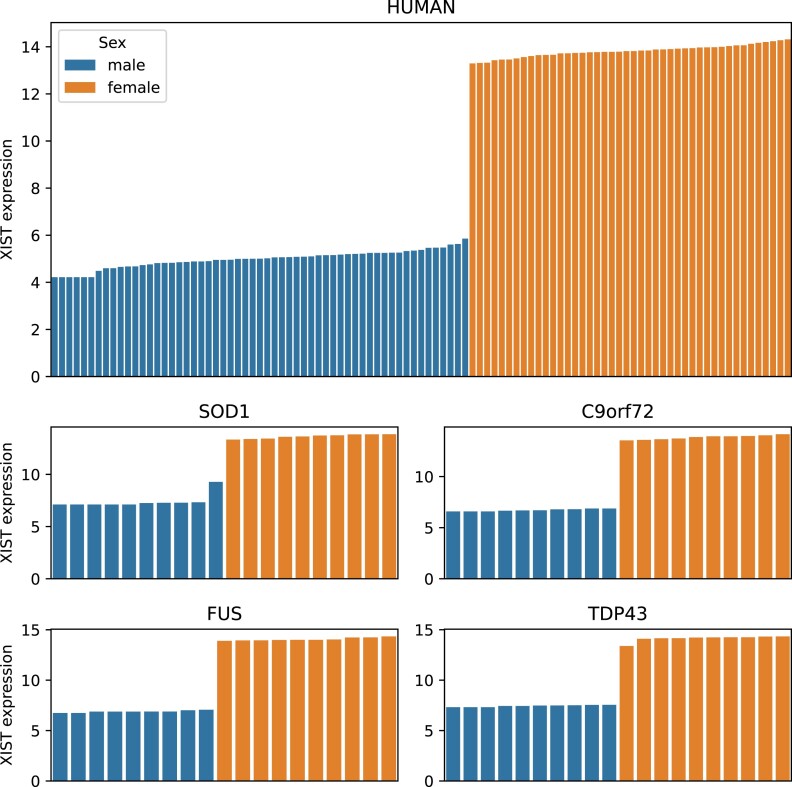
Verification of sex on the transcriptome level. VST-transformed *XIST* expression in human and mouse RNA-seq experiments colored by sex. *XIST* expression confirms the correct sex annotation.

In addition to the quality checks mentioned above, the quality of the dataset was evaluated. To verify the annotation of sex, the expression of *XIST* was investigated in each sample (Fig. 4). *XIST* is a long noncoding RNA, involved in X chromosome inactivation and therefore highly expressed in females [35]. We could not detect any mismatched sex annotation in the human or mouse samples (Fig. 4).

**Figure 5:**
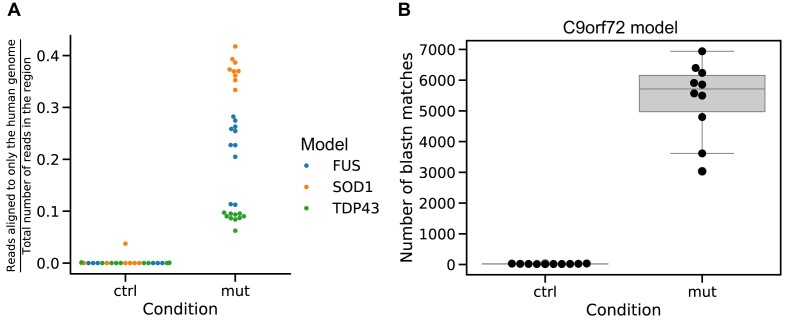
Verification of the transgenic animals. (A) Fraction of reads from the RNA-seq experiments aligning against the human genome in the region of the *Fus, Sod1*, and *Tardbp* genes (±200 bp) in the corresponding mouse model. Reads aligning against the human genome confirm that the corresponding samples express the transgenic transcript correctly. (B) Number of reads mapping to the pEGFP construct (U76561.1) using blastn [36] (v2.15.0) in C9orf72 transgenic animals.

Furthermore, we validated the expression of the transgenic variant for the FUS, SOD1, and TDP43 mouse models. The transgenic mouse models were generated by including the mutated human gene (SOD1 and TDP43) or overexpressing the wild-type human gene (FUS) in the mouse genome. Therefore, the fraction of reads aligning against this region of interest was compared to the total number of reads in that region (Fig. 5). The region of interest was defined as ±200 bp around the gene’s coding region. It is expected that control samples do not show any expression of human reads, while the mutated samples show a significant expression of the human variant. Thus, we could verify the expression of the transgene in these 3 mouse models (Fig. 5A).

**Figure 6:**
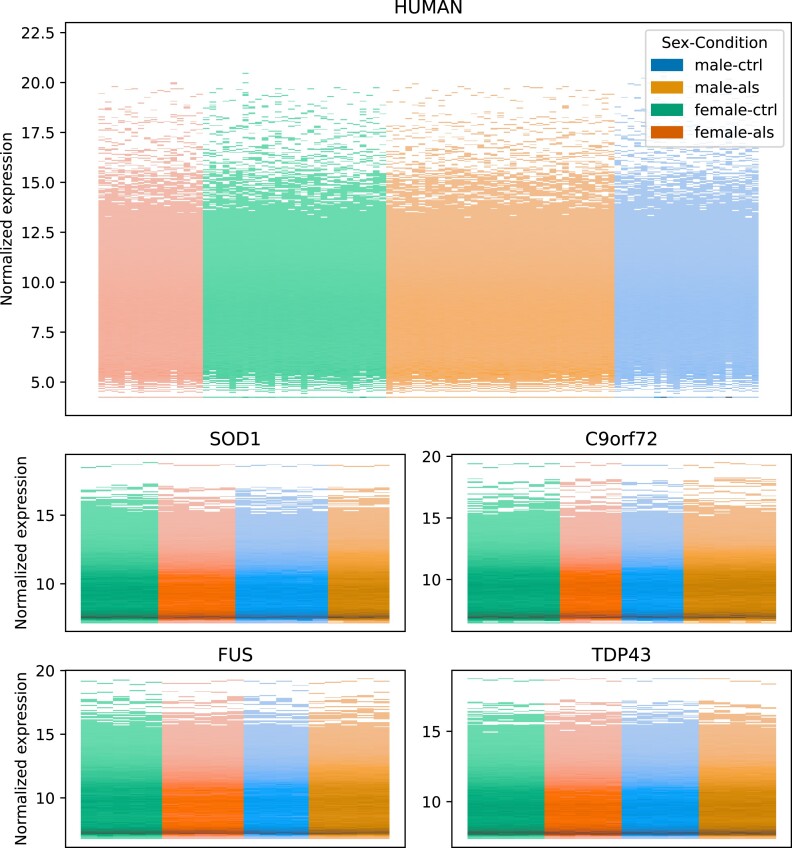
Overall quality of transformed transcriptomic data. Histogram of VST-transformed expression values with samples on the x-axis colored by sex and condition. No strong difference between the sexes and conditions could be observed.

The C9orf72 mouse model was generated by introducing a repeat expansion in the intronic region of C9orf72, which cannot be detected using the approach used for the other mouse models. Therefore, we used an indirect approach to detect the GFP expression of the construct used for integrating the repeat expansion [20]. We were able to detect the expression of the construct only in transgenic animals, thus indicating that the introduced repeat expansion is likely present as well in these animals (Fig 5B). Furthermore, the expression data were visualized using a histogram for each sample, showing no distinct pattern for individual samples (Fig. 6). Therefore, we consider the RNA-seq data good quality matching with the provided annotations.

### small RNA-seq data

**Figure 7:**
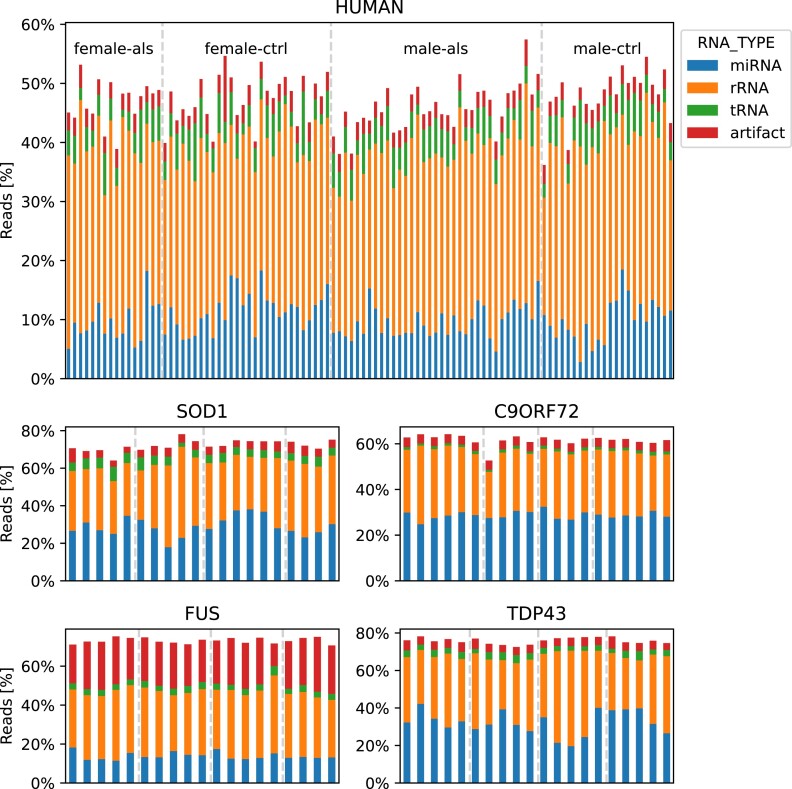
Evaluation of batch effects (sex and condition). Bar chart of miRTrace quality checks with samples on the x-axis colored by detected RNA type. The fraction of reads that could not be assigned to any of the RNA types is not displayed. No strong difference between the sexes or conditions could be observed.

The quality of the small RNA-seq data was additionally evaluated using miRTrace [27] as part of the Nextflow smrnaseq pipeline. miRTrace detected 17.07% reads as originating from miRNAs on average across the models (human: 10.12%, SOD1 29.05%, FUS: 13.37%, TDP43: 32.29%, C9orf72: 28.01%) and only a low percentage of artifacts (mean <6%). We could also not observe any large difference in the detected RNA types across samples (Fig. 7). However, the human samples showed a lower number of reads assigned to any class compared to the mouse samples.

**Figure 8:**
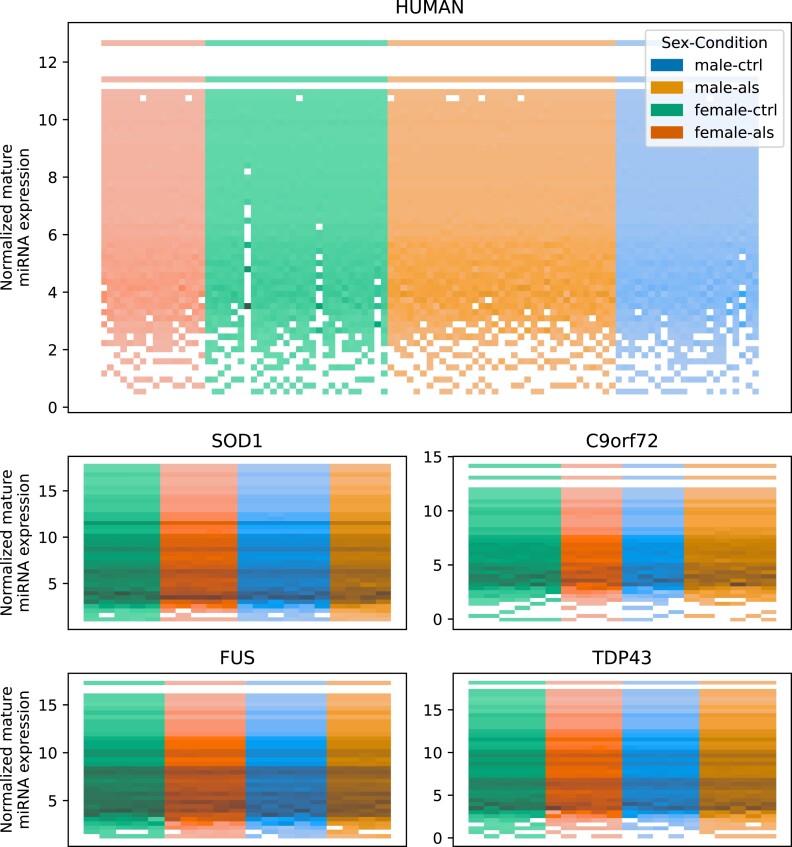
Overall quality of transformed small RNA-seq data. Histogram of normalized mature miRNA expression values with samples on the x-axis colored by sex and condition. No strong difference between the sexes and conditions could be observed.

Similar to the RNA-seq data, also a histogram of the miRNA expression was visualized (Fig. 8). The mouse models show a consistent expression pattern across samples, with only minor differences between the mouse models, conditions, and sexes. For the human samples, we observed a consistent expression pattern for most samples (Fig. 8).

### Proteomics data

**Figure 9:**
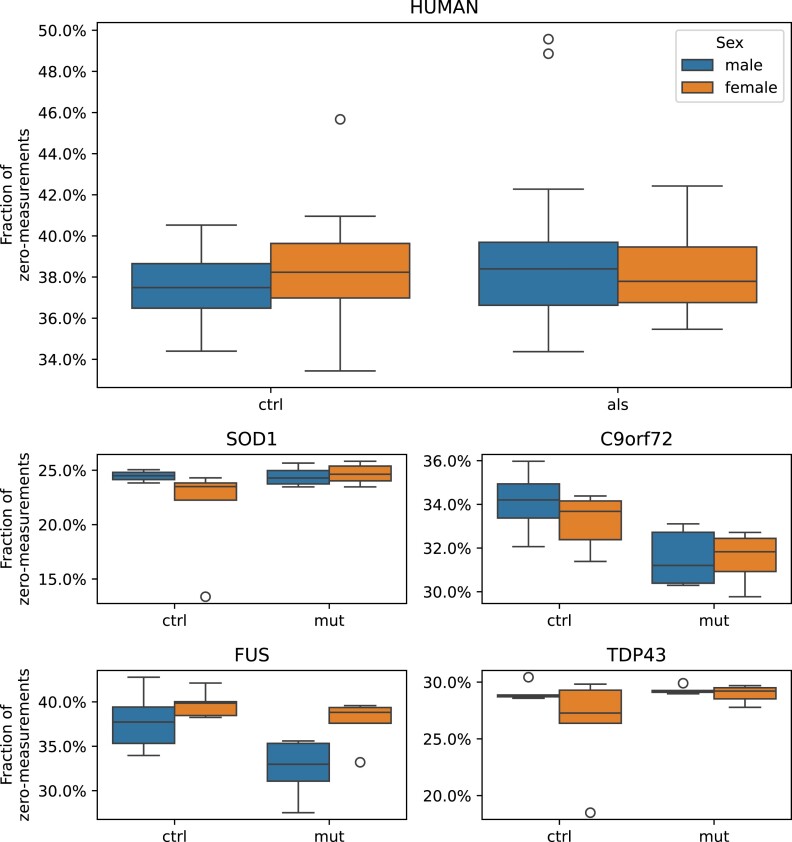
Completeness of raw proteomics data. Fraction of measured zeros in the proteomics experiments colored by sex. No difference in the distribution between the sexes and the condition could be detected.

**Figure 10:**
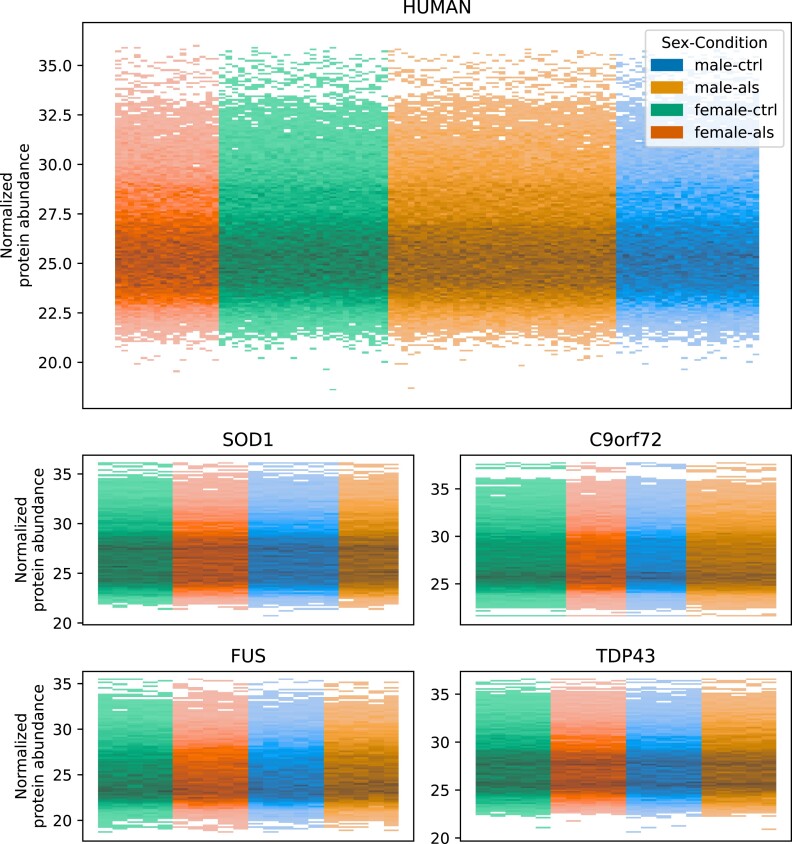
Overall quality of transformed proteomics data. Histogram of normalized protein abundance values with samples on the x-axis colored by sex and condition. No strong difference between the sexes and conditions could be observed.

**Figure 11:**
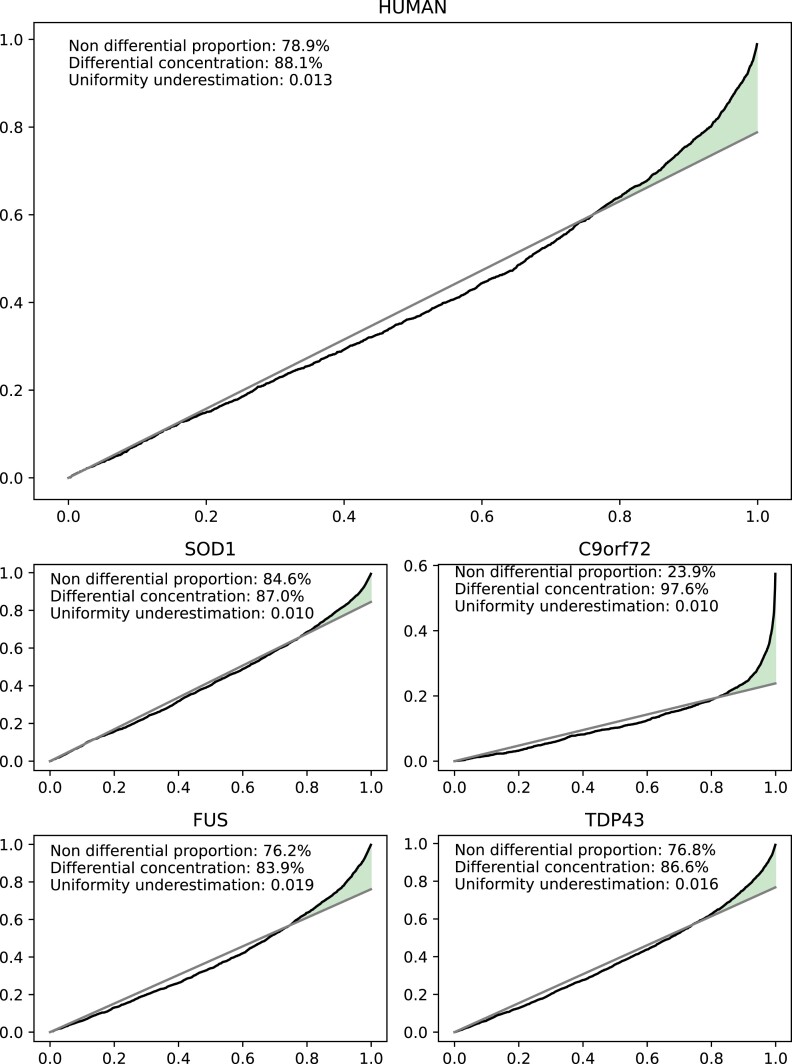
Evaluation of proteomics differential protein abundance analysis. Calibration plots for case vs. control differential protein abundance analysis to check if the *P* values respect the assumptions of classical FDR control procedures. A high (close to 100%) differential concentration (in green) and a low uniformity underestimation (close to 0) are preferred.

**Figure 12:**
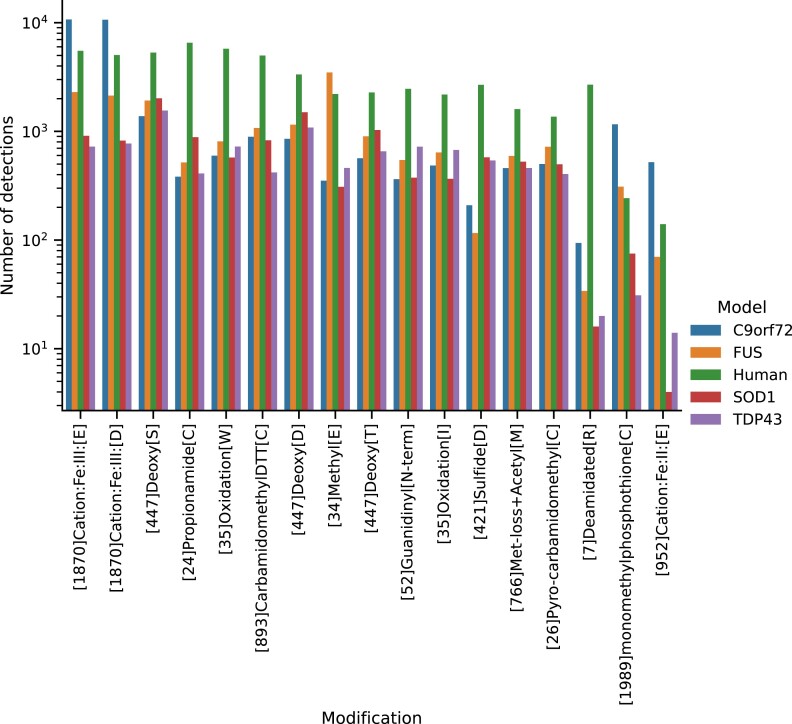
Top modifications found by the open modification search using ionbot for the 4 mouse models and human samples. For each model, the top 10 modifications were selected and the number of occurrences of the union of those (17 modifications) is displayed. Fixed modifications (Carbamidomethyl, Oxidation, Acetyl[N-term]) and sequence variations (Glu→Ser, Arg→Orn, Ser→Ala, Gln→pyro-Glu, Xle→Pro, Tyr→Phe, Delta:H(2)C(2)[N-term]) were removed for display.

The quality of the proteomics data was evaluated by calculating the fraction of measured zero values and the histogram of protein abundance values. We could not observe any significant difference between the fraction of zero measurements in the proteomics data (Fig. 9), indicating that there is no systematic bias impacting the sample quality. Furthermore, no systematic difference between the samples could be detected in the histograms of the normalized protein abundances (Fig. 10). Differential protein abundance analysis was conducted, and a calibration analysis was performed to verify that the obtained *P* values followed the assumptions of classical FDR control [37]. We detected a high differential protein abundance concentration (differential concentration >83%) and a low uniformity underestimation (<0.02) in all models (Fig. 11). This indicates that there are likely no violations of the FDR control assumptions. In addition, we performed an open modification search but did not detect any striking differences between the mouse models and human samples (Fig. 12). Overall, we detected no systematic biases, low intersample intramodel variability (especially for the mouse samples), and proven expression of the transgenes. Therefore, in our opinion, the dataset provides a unique resource for the (re)analysis of ALS considering multiple known ALS mouse models and human samples.

### Batch effects

**Figure 13:**
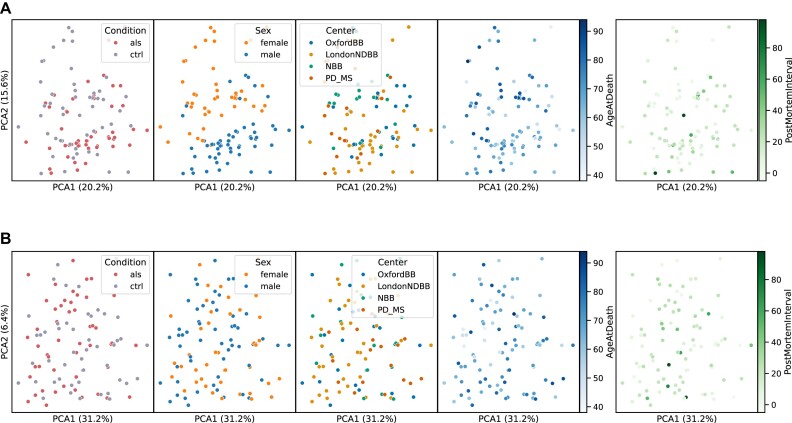
Evaluation of batch effects using PCA. PCA of human transcriptomics (A) and proteomics (B) data. The 500 most variable genes for the transcriptomics data and all proteins for the proteomics data were used.

To assess the possibility of batch effects in transcriptomics and proteomics data, PCA and sample distance heatmaps were used. Specifically, batch effects related to the factors brain bank, sex, case/control condition, and age at death were investigated. As reported in the previous publication (Fig. 13; Figure 1b of Caldi Gomes et al. [12]), sex-related differences were found, and all analyses were performed separately for each sex. For the other factors, there is no evidence that they influenced the results (Fig. 13; Supplementary Figure 1 of Caldi Gomes et al. [12]). For instance, if batch effects had been present, they would likely have caused distinct clustering or separation of samples. However, our analysis showed no such patterns, indicating that these factors did not introduce systematic biases into our data.

## Results Summary

In brief, in our initial study [12], this dataset revealed distinct molecular subclusters within patients with ALS. These subclusters showed varying patterns in gene, protein, and miRNA expression, suggesting the presence of different underlying disease mechanisms. One of these identified mechanisms was the MAPK pathway as a putative therapeutic target. Another important aspect of our study was the pronounced sex differences captured in the molecular profiles of patients with ALS, with male patients exhibiting more pronounced alterations overall.

The findings summarized here were validated across multiple models, reinforcing the significance of the identified molecular subclusters and pathways. This validation suggests that future ALS research should consider these frequently reported molecular differences and focus on developing personalized medicine approaches tailored to specific patient subgroups.

### Reuse potential

Our complex cross-species and sex-specific data can serve as a basis for future computational and experimental studies. Further, the stratification of patients with ALS into specific subtypes through our multiomics data could help with developing personalized, sex-specific, and efficient treatment approaches. Furthermore, newly found treatment candidates can be directly investigated in the 4 available mouse models to detect the potentially best mouse model for in vivo testing. Furthermore, this rich resource of human sALS and mouse models for gALS could be utilized to detect subtle differences between sALS and gALS (e.g., on splicing level), which are currently not well understood and can provide new biomarkers or treatment options in the early stages of ALS.

To facilitate future usage, intermediate files are saved in a format that is readable using most common programming languages, mainly in CSV format, allowing for flexible integration of new methods at every step of the existing pipeline. Several downstream applications, such as differential gene expression analysis, are already implemented and can be executed using DVC. Furthermore, these methods are highly configurable using the parameter files and allow for a multitude of different analyses. To achieve continuous high reproducibility, we recommend the implementation of executable scripts, which can be automatically executed by DVC.

In addition, the repository provides code for the analysis of transcription factor activity, RNA stability, and possible RNA variants. Further details can be found in the 3 following sections.

### Transcription factor activity

Transcription factor activity was estimated using decoupleR [38] with default settings. For activity estimation, a univariate linear model, a weighted sum, and a multivariate linear model were used as recommended by decoupleR. The DoRothEA [39] database was used for potential transcription factor targets. Only targets of at least category C were used (category A = high confidence, category E = low confidence).

### RNA stability analysis

RNA stability analysis was performed using REMBRANDTS [40]. Exon and intron regions, as required for the analysis with REMBRANDTS, were extracted from GENCODE v37 annotations for the human data and GENCODE vM26 for the mouse data. The quantification of exon and intron abundance was performed using htseq (v1.99.2) as described in Alkallas et al. [40] and the REMBRANDTS manual. REMBRANDTS was run using default arguments with a linear bias mode, a stringency of 0.99, and no further batch information.

### Variant analysis

To analyze variations in the mRNA, we performed variant calling on the RNA-seq data. Bcftools (v1.14) was used for variant calling based on the STAR alignments, as provided by the NextFlow Core RNA-seq pipeline, version 3.0, described in Ewels et al. [17].

## Conclusion

In summary, our study provides a valuable data resource, including sex-specific and cross-species datasets. The stratified multiomics data from ALS prefrontal cortices highlights male and female differences, with implications for future personalized treatment approaches. Additionally, we offer a robust analysis pipeline and high-quality data for investigating early ALS mechanisms.

Our methods ensure robust comparability and reproducibility of the analysis across all generated datasets, both within omics layers for the analyzed cohorts and also across species. Overall, these datasets can help with the understanding of ALS pathogenesis and assist in identifying new and personalized therapeutic targets for this devastating neurodegenerative disease.

## Supplementary Material

giae100_GIGA-D-24-00236_Original_Submission

giae100_GIGA-D-24-00236_Revision_1

giae100_Response_to_Reviewer_Comments_Original_Submission

giae100_Reviewer_1_Report_Original_Submission Adam Trautwig -- 7/23/2024

giae100_Reviewer_1_Report_Revision_1 Adam Trautwig -- 10/7/2024

giae100_Reviewer_2_Report_Original_Submission Lu Zeng -- 8/5/2024

giae100_Reviewer_2_Report_Revision_1 Lu Zeng -- 10/8/2024

## Data Availability

The workflow contains scripts for the automatic download of all mouse sequencing data from SRA to be used as input to the workflow. However, the workflow can also be used with manually downloaded files, which is required for human samples, due to the restricted access. Details about how to access the human data deposited in European Genome Phenome Archive are available online [41]. All supporting data and materials are available in the *GigaScience* database, GigaDB [42] and in WorkflowHub [43]. Raw RNA-seq data (FASTQ format) and processed data (CSV format) were deposited to the National Center for Biotechnology Information Gene Expression Omnibus database (GSE234245) and are openly available. Raw small RNA-seq data (FASTQ format) and processed data (CSV format) were deposited to the National Center for Biotechnology Information Gene Expression Omnibus database (GSE234243) and are openly available. Human raw data (FASTQ format) are encrypted and stored at the European Genome Phenome Archive (registered study: EGAS00001007318). These data are available upon request to the European Genome Phenome Archive. Details about how to access the human data deposited in European Genome Phenome Archive are available online [41]. Human and mouse proteomics data were deposited to the ProteomeXchange Consortium database (PXD043300) and are openly available. The results from the open modification search are available via Figshare [44].

## Availability of Source Code and Requirements

Project name: MAXOMOD

Project homepage: https://github.com/imsb-uke/MAXOMOD_Pipeline

Operating system(s): Platform independent

Programming language: Python, R, Bash, Nextflow

Other requirements: DVC (https://dvc.org/), Docker

License: MIT

Workflowhub.eu: 10.48546/workflowhub.workflow.1191.1.

All code for preprocessing and analyzing the data is available online [45]. The workflow consists of multiple scripts R and Python (src/directory), which can be executed in a dockerized environment, which is provided on GitHub as well. The execution order is provided as a Data Version Control workflow, which can be automatically executed with DVC. All parameters are provided in the params/directory. Further information can be found in the README file in the GitHub repository.

## Abbreviations

ALS: amyotrophic lateral sclerosis; CTR: control; DVC: Data Version Control; FDR: false discovery rate; gALS: genetic amyotrophic lateral sclerosis; PBS: phosphate-buffered saline; PCA: principal component analysis; PFC: prefrontal cortex; RNA-seq: RNA sequencing; sALS: sporadic amyotrophic lateral sclerosis.
