# Supplementary material for: A dataset profiling the multiomic landscape of the prefrontal cortex in amyotrophic lateral sclerosis
Source: Gigascience. 2024 Dec 18;13:giae100. doi: 10.1093/gigascience/giae100 (PMC11653894; doi:10.1093/gigascience/giae100)
Supplement: giae100_GIGA-D-24-00236_Original_Submission [file giae100_giga-d-24-00236_original_submission.pdf]

GigaScience

A Data Set Profiling the Multi-omic Landscape of the Prefrontal Cortex in Amyotrophic Lateral Sclerosis

--Manuscript Draft--

**Manuscript Number:**	GIGA-D-24-00236
**Full Title:**	A Data Set Profiling the Multi-omic Landscape of the Prefrontal Cortex in Amyotrophic Lateral Sclerosis
**Article Type:**	Data Note
**Funding Information:**	Bundesministerium für Bildung und Forschung (01GM1917A) — Prof Paul Lingor<br>Munich Cluster for Systems Neurology — Prof Paul Lingor<br>BMBF (grant 01GM2202A; STOP-FSGS and DFG, CRC1192) — Dr Sonja Hänzelmann<br>CRC1286, CRC1192 — Prof Stefan Bonn
**Abstract:**	Amyotrophic lateral sclerosis is the most common motor neuron disease, which still lacks effective disease-modifying therapies. Similar to other neurodegenerative disorders, such as Alzheimer's and Parkinson's disease, ALS pathology is presumed to propagate over time, originating from the motor cortex and spreading to other cortical regions. Exploring early disease stages is crucial to understand the causative molecular changes underlying the pathology. For this, we sampled human postmortem prefrontal cortex (PFC) tissue from Brodmann area 6, an area that exhibits only moderate pathology at the time of death, and performed a multiomic analysis of 51 sporadic ALS patients and 50 control subjects. To compare sporadic disease to genetic ALS, we additionally analyzed PFC tissue from four transgenic ALS mouse models (C9orf72-, SOD1-, TDP-43-, and FUS-ALS) using the same methods. This multiomic data resource includes transcriptome, small RNAome and proteome data from female and male samples, aimed at elucidating early and sex-specific ALS mechanisms, biomarkers, and drug targets.
**Corresponding Author:**	Paul Lingor<br>Technical University of Munich: Technische Universitat Munchen<br>München, GERMANY
**Corresponding Author Secondary Information:**	
**Corresponding Author's Institution:**	Technical University of Munich: Technische Universitat Munchen
**Corresponding Author's Secondary Institution:**	
**First Author:**	Fabian Hausmann
**First Author Secondary Information:**	
**Order of Authors:**	Fabian Hausmann<br>Lucas Caldi Gomes<br>Sonja Hänzelmann<br>Robin Khatri<br>Sergio Oller<br>Mojan Parvaz<br>Laura Tzeplaeff<br>Laura Pasetto<br>Marie Gebelin

Powered by Editorial Manager® and ProduXion Manager® from Aries Systems Corporation

	Qihui Zhou
	Pavol Zelina
	Dieter Edbauer
	R. Jeroen Pasterkamp
	Hubert Rehrauer
	Ralph Schlapbach
	Christine Carapito
	Valentina Bonetto
	Stefan Bonn
	Paul Lingor
**Order of Authors Secondary Information:**	
**Additional Information:**	
**Question**	**Response**
Are you submitting this manuscript to a special series or article collection?	No
**Experimental design and statistics**<br><br>Full details of the experimental design and statistical methods used should be given in the Methods section, as detailed in our Minimum Standards Reporting Checklist. Information essential to interpreting the data presented should be made available in the figure legends.<br><br>Have you included all the information requested in your manuscript?	Yes
**Resources**<br><br>A description of all resources used, including antibodies, cell lines, animals and software tools, with enough information to allow them to be uniquely identified, should be included in the Methods section. Authors are strongly encouraged to cite Research Resource Identifiers (RRIDs) for antibodies, model organisms and tools, where possible.<br><br>Have you included the information requested as detailed in our Minimum Standards Reporting Checklist?	Yes

Powered by Editorial Manager® and ProduXion Manager® from Aries Systems Corporation

**Availability of data and materials**<br><br>All datasets and code on which the conclusions of the paper rely must be either included in your submission or deposited in publicly available repositories (where available and ethically appropriate), referencing such data using a unique identifier in the references and in the "Availability of Data and Materials" section of your manuscript.<br><br>Have you have met the above requirement as detailed in our Minimum Standards Reporting Checklist?	Yes

Manuscript

Click here to access/download;Manuscript;Hausmann, Caldi Gomes and Haenzelmann et al. Giga Science.docx

A Data Set Profiling the Multi-omic Landscape of the Prefrontal Cortex in Amyotrophic Lateral Sclerosis

Fabian Hausmann[1*], Lucas Caldi Gomes[3*], Sonja Hänzelmann[1,4*], Robin Khatri[1], Sergio Oller[1], Mojan Parvaz[3], Laura Tzeplaeff[3], Laura Pasetto[5], Marie Gebelin[6], Qihui Zhou[8,9], Pavol Zelina[10], Dieter Edbauer[8,9], R. Jeroen Pasterkamp[10], Hubert Rehrauer[7], Ralph Schlapbach[7], Christine Carapito[6], Valentina Bonetto[5], Stefan Bonn[1,2§], Paul Lingor[3,8,9§,#]

1 Institute of Medical Systems Biology, Center for Biomedical AI (bAIome), Center for Molecular Neuroscience (ZMNH), University Medical Center Hamburg-Eppendorf, 20251 Hamburg, Germany
2 Hamburg Center for Translational Immunology (HCTI), University Medical Center Hamburg-Eppendorf, Hamburg 20251, Germany
3 Technical University of Munich, School of Medicine, rechts der Isar Hospital, Clinical Department of Neurology, Munich, Germany.
4 III Department of Medicine, University Medical Center Hamburg-Eppendorf, Hamburg, Germany.
5 Research Center for ALS, Istituto di Ricerche Farmacologiche Mario Negri IRCCS, Milan, Italy
6 Laboratoire de Spectrométrie de Masse Bio-Organique, Université de Strasbourg, Infrastructure Nationale de Protéomique, Strasbourg, France.
7 Functional Genomics Center Zürich, ETH Zürich and University of Zürich, Zürich, Switzerland.
8 German Center for Neurodegenerative Diseases (DZNE), München, Germany.
9 Munich Cluster for Systems Neurology (SyNergy), Munich, Germany.
10 Department of Translational Neuroscience, University Medical Center Utrecht, Utrecht University, Utrecht, The Netherlands.

*,[§] These authors contributed equally.

[#]Corresponding Author:

Paul Lingor; email: paul.lingor@tum.de; Clinical Department of Neurology, School of Medicine, rechts der Isar Hospital, Technical University of Munich, Ismaninger Straße 22, 81675 Munich, Germany, Tel.: +498941408257

Abstract

Amyotrophic lateral sclerosis is the most common motor neuron disease, which still lacks effective disease-modifying therapies. Similar to other neurodegenerative disorders, such as Alzheimer's and Parkinson's disease, ALS pathology is presumed to propagate over time, originating from the motor cortex and spreading to other cortical regions. Exploring early disease stages is crucial to understand the causative molecular changes underlying the pathology. For this, we sampled human postmortem prefrontal cortex (PFC) tissue from Brodmann area 6, an area that exhibits only moderate pathology at the time of death, and performed a multiomic analysis of 51 sporadic ALS patients and 50 control subjects. To compare sporadic disease to genetic ALS, we additionally analyzed PFC tissue from four transgenic ALS mouse models (C9orf72-, SOD1-, TDP-43-, and FUS-ALS) using the same methods. This multiomic data resource includes transcriptome, small RNAome and proteome data from female and male samples, aimed at elucidating early and sex-specific ALS mechanisms, biomarkers, and drug targets.

Keywords: Amyotrophic lateral sclerosis; multi-omics analysis; neurodegeneration; prefrontal cortex; early disease mechanisms

Context

Amyotrophic lateral sclerosis (ALS) is a devastating motor neuron disease characterized by progressive paralysis and a shortened lifespan following symptom onset[1]. While the majority of ALS cases are sporadic (sALS) and lack a clear genetic predisposition, approximately 10% are associated with known genetic mutations (gALS)[2]. Among the most common genetic causes are mutations in the genes of *C9orf72*, *SOD1*, *TARDBP*, and *FUS.* Interestingly, a subset of sALS patients also harbor disease-causing mutations[2–4]. Despite considerable research efforts, the exact etiology of sALS remains elusive, and effective disease-modifying treatments are currently unavailable[1,5,6]. Understanding the early mechanisms of ALS pathology is paramount for identifying diagnostic biomarkers and uncovering more effective therapeutic targets. Many investigations into ALS pathology have focused on end-stage

disease by using postmortem CNS tissue, which may obscure insights into earlier disease mechanisms that could offer more promising therapeutic avenues[7–9]. In contrast to the motor cortex, which is affected early in the disease and therefore often shows end-stage alterations at the death of the patients[7], the prefrontal cortex (PFC) is affected only later in the disease and thus presents a unique opportunity to explore earlier ALS pathology[10,11]. Histological studies have revealed that while the motor cortex exhibits severe pathology in later stages of the disease[7–9], the PFC demonstrates intermediate TDP-43 pathology, suggesting its relevance in elucidating earlier disease-mediated alterations[10,12]. A recent study employed multiomics to profile the molecular alterations in the spinal cord, another region heavily affected in ALS[13]. Other studies employed multiomic strategies in postmortem tissue from ALS patients, but they included only a limited number of techniques, focusing on transcriptome- or genome-based technologies[14,15]. Studies focusing on early alterations in ALS-affected brains in a comprehensive multiomic setting are still lacking[16].

In this context, the availability of omics datasets and robust analytical workflows is critical for advancing ALS research. Building upon our previous work[12], we improved the accessibility of raw and processed data, alongside detailed descriptions of bioinformatics methodologies. This includes extensive documentation of bioinformatics workflows and the provision of code to facilitate reproducibility and transparency in data analysis.

We provide a broad multi-omic high-throughput sequencing data set of a cohort of 101 human samples from four different brain banks (N = 51 patients with sporadic ALS; N = 50 control subjects, males and females). The omic layers encompass mRNAomics, small RNAomics, and proteomics. Additionally, we provide corresponding data for four distinct ALS mouse models based on mutations in the genes of *SOD1*, *C9orf72*, *FUS*, and *TARDBP*. Each mouse model includes both male and female samples, with transgenic and wild-type groups represented in each omic layer (cohort numbers balanced for sex and condition) ensuring a robust comparative analysis across species. Human PFC samples were provided by four different European brain banks (London Neurodegenerative Diseases Brain Bank, the Imperial College London - Multiple Sclerosis and Parkinson's Tissue Bank, the Oxford Brain Bank, and the

Netherlands Brain Bank). Subjects composing the control cohort did not present any signs of neurodegenerative diseases. Clinical features provided include age at death, postmortem interval (until the brains were sampled), disease onset, disease duration, and brain bank.

Here, we describe a findable, accessible, interoperable, and reproducible (FAIR) multiomics analysis workflow including integration steps and accompanying data that are freely available. For this, we used standardized and versioned docker containers provided by Nextflow[19] for the preprocessing steps and documented the data-specific statistical and machine-learning analyses. An overview of this workflow can be found in Fig. 1.

Methods

Experimental Data Acquisition and Preparation

Sample Data Description

This study investigates the molecular mechanisms underlying ALS using samples from human PFC and from four transgenic mouse models. The human cohort includes 51 patients with sALS and 50 control (CTR) subjects (Table 1). The ALS animal models include four genetically modified mouse strains: B6;129S6-Gt(ROSA)26Sortm1(TARDBP*M337V/Ypet)Tlbt/J mice (here simply referred to as TDP-43 mice)[20], B6SJL-Tg(SOD1*G93A)1Gur/J mice[21] (here referred to as SOD1 mice), (Poly)GA-NES/C9orf72(R26(CAG-Isl-175GA)-29×Nes-Cre) mice (here referred to as C9orf72 mice)[22], and Tg (Prnp-FUS)WT3Cshw/J mice (hereafter referred to as FUS mice)[23]. Each animal model cohort consists of ten transgenic and ten non-transgenic mice, balanced for sex (Table 2).

Sample Acquisition Methods

Ethics, consent and permissions

Data collection and handling is reported in Caldi Gomes, Hänzelmann et al. (2024)[12]. All experimental data presented here comply with the relevant ethical regulations. Consent for the donation of brain material for the subjects that

compose the human cohorts was handled individually by the brain banks involved in this study. Ethical approval was obtained from the Ethics Committees of the University Medical Center Göttingen (2/8/18 AN) and the Technical University Munich (145/19 S-SR). All animal experiments complied with international and local animal welfare laws and were approved by the respective regulatory organs for each involved research center. Experiments with transgenic SOD1 and FUS mice were prospectively approved by the Mario Negri Institutional Animal Care and Use Committee and the Italian Ministry of Health (Prot. No. 9F5F5.143/Prot. No. 9F5F5.250). Experiments with C9orf72 transgenic mice followed the regulations from the German Animal Welfare Act (Tierschutzgesetz/Tierschutz-Versuchstierverordnung, Regierungsbezirke Oberbayern, Prot. No. TV 55.2-2532.Vet_02-17-106). Experiments with TDP43 transgenic animals were approved by the (CCD) Centrale Commissie Dierproeven of Utrecht University (CCD license: AVD 1150020171565), in accordance with Dutch animal welfare laws (Wet op de Dierproeven 2014) and European regulations (guideline 2010/63/EU).

Human Postmortem PFC Samples

Human PFC samples were sourced from four brain banks: The Netherlands Brain Bank, London Neurodegenerative Diseases Brain Bank, Imperial College London Multiple Sclerosis and Parkinson's Tissue Bank, and the Oxford Brain Bank. Samples were shipped on dry ice and stored at -80 °C upon arrival at the rechts der Isar Hospital Department of Neurology, Technical University of Munich. PFC samples were sectioned using a cryostat at -20 °C and processed to collect approximately 20 mg of tissue per sample, which was stored at -80 °C until further use.

ALS Animal Models

Four transgenic mouse models were used to represent the most frequent ALS-causing genes. The mice were kept in pathogen-free facilities with a 12-hour light/dark cycle and unrestricted access to food and water. Each mouse model was euthanized at specific presymptomatic or early symptomatic stages for

biomaterial collection. Euthanization times were: TDP-43 (26 weeks), SOD1 (14 weeks), C9orf72 (4.5 weeks), and FUS (four weeks). Mice were perfused with ice-cold phosphate-buffered saline (PBS) before microdissection. The prefrontal cortex was isolated, transferred to nuclease-free tubes, and stored at -80 °C until RNA and protein isolation. For each model, a total of 20 transgenic and control (wild type) mice were selected and balanced for condition/sex (TDP-43: 5 females and 5 males for transgenic and control cohorts; SOD1: 5 females and 6 males for the control cohort; 5 females and 4 males for the transgenic cohort; C9orf72: 6 females and 4 males for the control cohort; 4 females and 6 males for the transgenic cohort; FUS: 5 females and 5 males for transgenic and control cohorts).

RNA Isolation

Total RNA from human and animal PFC samples was isolated using TRIzol Reagent. RNA was precipitated, washed with ethanol, reconstituted in nuclease-free water, and treated with DNase to remove DNA contamination. Nucleic acid concentration and purity were assessed using a NanoDrop One spectrophotometer and an Agilent 6000 NanoKit for RNA integrity.

RNA Sequencing

mRNA and small RNA sequencing (RNAseq) experiments were conducted at the Functional Genomics Center Zürich. For mRNA sequencing, the TruSeq Stranded mRNA Kit and the SMARTer Stranded Total RNA-Seq Kit v2 Pico Input Mammalian were used. The RealSeq-AC miRNA was used for small RNAseq experiments. After library preparation, normalization was done using Tris–Cl (pH 8.5) containing 0.1% Tween 20 (at 10 nM for the TruSeeq kit, 5 nM for the SMARTer Stranded kit, and 2nM for the RealSeq-AC miRNA kit). Sequencing was performed in the Illumina NovaSeq 6000 platform (for RNAseq) and the HiSeq 2500 platform (for small RNAseq).

Proteomics

Proteins from human and mouse PFC tissue samples were extracted with a biosmasher using 350 µL of MeOH:H_2O (4:1), resuspended in 200 µL Laemmli buffer (10% SDS, Tris 1M pH 6.8, glycerol) then centrifuged at 11.135 rpm at 4°C for 5 minutes. 100 µg of protein lysate was denatured by heating at 95°C for 5 minutes and stacked in an in-house prepared 5% acrylamide SDS-PAGE stacking gel. Gel bands were reduced and alkylated. Digestion was performed overnight at 37°C using modified porcine trypsin (Mass Spec Grade, Promega, Madison, USA; enzyme:protein ratio of 1:80). The peptides were extracted by sequential application of 60% acetonitrile and 100% acetonitrile (ACN). Peptides were resuspended in 30 µL of H_20, 2% ACN, 0.1% FA and iRT peptides (Biognosys, Schlieren, Switzerland) were added according to the manufacturer's instructions. The generated samples were analyzed using nanoLC–MS/MS (nanoAcquity UltraPerformance LC, Waters, Milford, MA, USA), coupled to a Q-Exactive Plus Mass Spectrometer (Thermo Fisher Scientific). Data was further processed using MaxQuant[24].

Data Preparation

mRNAseq and small RNAseq Data Processing

RNAseq data were processed using the Nextflow[19] Core RNASeq pipeline version 3.0 with the following parameters *--igenomes_ignore true --fasta <version>.genome.fa.gz --gtf gencode.<version>.annotation.gtf.gz --pseudo_aligner salmon --gencode --deseq2_vst.* Quality checks were conducted with FastQC[25] (Fig. 2) and preprocessing steps included adapter trimming and quality filtering to remove low-quality reads and artifacts. Salmon[26] was used for pseudo-alignment and quantitation, with indices built from GENCODE[27] annotations GRCm39 for mouse and GRCh38 for human. Small RNAseq data were processed using the Nextflow[19] Core smRNASeq pipeline version 1.0 with the parameters *--genome <genome> --mirna_gtf mirbase_<species>.gff3*. FastQC[25] (Fig. 3) and miRTrace[28] were used for quality checks, followed by adapter

trimming and quality filtering. Alignment was performed with Bowtie[29], and feature counting utilized samtools[30] using miRBase[31] annotations (version 22.1).

Filtering and Transformation

For RNAseq and small RNAseq count matrices were filtered to retain features with at least ten counts in 50% of samples for any condition or sex. For the small RNAseq data normalization was performed using quantile normalization implemented in the preprocessCore[32] R package. For RNAseq, variance-stabilizing transformation (VST) implemented in DESeq2[33] was used for normalization, ensuring consistent and comparable expression values across samples.

Proteomics

Proteomics data were processed with MaxQuant[24] software. Protein peaks were assigned using trypsin/P specificity against an in-house generated protein sequence database containing mouse entries from UniProtKB-SwissProt. The "match between runs" option facilitated protein quantification. Only Swiss-Prot proteins were retained and low-abundance proteins detected in less than 50% of samples were filtered out. Missing values were imputed using the missForest[34] algorithm and intensities were log2-transformed for variance stabilization. A maximum false discovery rate (FDR) of 1% was applied at both peptide and protein levels.

Higher-Level Data Analysis and Machine Learning

Differential Expression and Enrichment Analyses

Downstream analyses of the RNAseq and small RNAseq data included differential expression analysis using DESeq2[33] to identify differentially expressed genes/miRNAs between experimental conditions. Principal

Component Analysis (PCA) was used for dimensionality reduction and visualization of sample relationships using VST-normalized RNAseq data and quantile-normalized small RNAseq data.

Proteomics Data Analysis

Linear modeling for differential abundance analysis was performed using the limma[35] package, with p-values adjusted for multiple testing using the Benjamini–Hochberg correction. PCA was used for visualization.

Bioinformatics workflow

To allow for reproducible and interpretable bioinformatics workflow, we'll describe the construction of our workflow here. Our workflow consists of multiple stages, equivalent to single scripts executed for one or multiple datasets with multiple parameters. We used Data Version Control (DVC) as a workflow management tool, because it allows the use of any script as stages in our computational workflow, automatically takes care of dependencies between these stages, and executes only stages that changed compared to the last execution. Furthermore, it provides the option to share raw and processed data between multiple users. The executed code for each stage is provided as script files written in bash, R, and Python. The execution of the scripts, their outputs, and their dependencies are defined in dvc.yaml files, with all important parameters found in params.yaml files. All scripts can be automatically executed using DVC. Since the execution of scripts depends on the package versions used, in R as well as in Python, proper maintenance of package versions is important.

Therefore we containerized all applications by providing a docker image. In other instances, we used readily available docker images. This allows the automatic execution of our workflow using docker, if available, and prevents users from struggling to install the correct package versions. Furthermore, we value community efforts in providing reproducible workflows for the analysis of RNAseq & small RNAseq data implemented in Nextflow. These pipelines were

integrated into our workflow as well, allowing us to easily adapt to recent developments in the workflow without much user effort.
All outputs of our workflow will be structured by mouse model and can be used for other applications, integrated into the workflow or not. However, we recommend integrating further analysis into the DVC workflow, as not to break the reproducibility principle of the extended workflow. In this case, also newly added scripts will automatically be executed if the underlying data or scripts are changing in any way.

Data Validation and quality control

RNAseq Data

In addition to the quality checks mentioned above, the quality of the dataset was evaluated. To verify the annotation of sex, the expression of *XIST* was investigated in each sample (Fig. 4). *XIST* is a long non-coding RNA, involved in X-chromosome-inactivation, and therefore highly expressed in females[36]. We could not detect any mismatched sex annotation in the human or mouse samples (Fig. 4).
Furthermore, we validated the expression of the transgenic variant for the FUS, SOD1, and TDP43 mouse models. The transgenic mouse models were generated by including the mutated human gene (SOD1 and TDP43) or overexpressing the wild-type human gene (FUS) in the mouse genome. Therefore, the fraction of reads aligning against this region of interest was compared to the total number of reads in that region (Fig. 5). The Region of interest was defined as ±200 bp around the gene's coding region. It is expected that control samples don’t show any expression of human reads, while the mutated samples show a significant expression of the human variant. Thus, we could verify the expression of the transgene in these three mouse models (Fig. 5A).
The C9orf72 mouse model was generated by introducing a repeat expansion in the intronic region of C9orf72, which cannot be detected using the approach used for the other mouse models. Therefore, we used an indirect approach to detect the GFP expression of the construct used for integrating the repeat expansion[22]. We were able to detect the expression of the construct only in

transgenic animals, thus indicating that the introduced repeat expansion is likely present as well in these animals (Fig 5B). Furthermore, the expression data was visualized using a histogram for each sample, showing no distinct pattern for individual samples (Fig. 6). Therefore, we consider the RNAseq data good quality matching with the provided annotations.

small RNAseq Data

The quality of the small RNAseq data was additionally evaluated using miRTrace[28] as part of the Nextflow smrnaseq pipeline. miRTrace detected 17.07% reads as originating from miRNAs on average across the models (Human: 10.12%, SOD1 29.05%, FUS: 13.37%, TDP43: 32.29%, C9orf72: 28.01%) and only a low percentage of artifacts (mean < 6%). We could also not observe any large difference in the detected RNA types across samples (Fig. 7). However, the human samples showed a lower number of reads assigned to any class compared to the mouse samples.
Similar to the RNAseq data also a histogram of the miRNA expression was visualized (Fig. 8). The mouse models show a consistent expression pattern across samples, with only minor differences between the mouse models, conditions, and sexes. For the human samples, we observed a consistent expression pattern for most samples (Fig. 8).

Proteomics Data

The quality of the proteomics data was evaluated by calculating the fraction of measured zero values and the histogram of protein abundance values. We could not observe any significant difference between the fraction of zero-measurements in the proteomics data (Fig. 9), indicating that there is no systematic bias impacting the sample quality. Furthermore, no systematic difference between the samples could be detected in the histograms of the normalized protein abundances (Fig. 10). Differential protein abundance analysis was conducted and a calibration analysis was performed to verify that the obtained p-values followed the assumptions of classical false discovery rate (FDR) control[37]. We detected a high differential protein abundance

concentration (differential concentration > 83%) and a low uniformity underestimation (< 0.02) in all models (Fig. 11). This indicates that there are likely no violations of the FDR control assumptions. Overall, we detected no systematic biases, low inter-sample intra-model variability (especially for the mouse samples), and proven expression of the transgenes. Therefore, in our opinion, the dataset provides a unique resource for the (re-)analysis of ALS considering multiple known ALS mouse models and human samples.

Re-use potential

Our complex cross-species and sex-specific data can serve as a basis for future computational and experimental studies. Further, the stratification of ALS patients into specific subtypes through our multi-omic data could help with developing personalized, sex-specific, and efficient treatment approaches. Furthermore, newly found treatment candidates can be directly investigated in the four available mouse models, to detect the potentially best mouse model for in vivo testing. Furthermore, this rich resource of human sALS and mouse models for gALS could be utilized to detect subtle differences between sALS and gALS, e.g. on splicing level, which are currently not well understood and can provide new biomarkers or treatment options in the early stages of ALS. To facilitate future usage, intermediate files are saved in a format that is readable using most common programming languages, mainly in CSV format, allowing for flexible integration of new methods at every step of the existing pipeline. Several downstream applications, such as differential gene expression analysis, are already implemented and can be executed using DVC. Furthermore, these methods are highly configurable using the parameter files and allow for a multitude of different analyses. To achieve continuous high reproducibility, we recommend the implementation of executable scripts, which can be automatically executed by DVC.

Conclusion

In summary, our study offers a dual advantage for the scientific community by providing an extensive and rich data resource that includes sex-specific and

cross-species datasets, which can be foundational for future computational and experimental research. This stratified multi-omics data from ALS prefrontal cortices, which includes differences between male and female responses to ALS, can aid in developing personalized and sex-specific treatment approaches. Additionally, we offer a well-documented and robust analysis pipeline. In addition, our analyses provide a high-quality data resource for investigating early ALS mechanisms.

Our methods ensure robust comparability and reproducibility of the analysis across all generated datasets, both within omics layers for the analyzed cohorts, and also across species. Overall, these datasets can help with the understanding of ALS pathogenesis and assist in identifying new and personalized therapeutic targets for this devastating neurodegenerative disease.

Data Availability

The workflow contains scripts for the automatic download of all mouse sequencing data from SRA to be used as input to the workflow. However, the workflow can also be used with manually downloaded files, which is required for human samples, due to the restricted access. Details about how to access the human data deposited in European Genome Phenome Archive are available here: https://ega-archive.org/access/request-data/how-to-request-data/.

Mouse RNAseq data

Raw RNAseq data (FASTQ format) and processed data (CSV format) were deposited to the National Center for Biotechnology Information Gene Expression Omnibus database (GSE234245) and are openly available.

Mouse small RNAseq data

Raw small RNAseq data (FASTQ format) and processed data (CSV format) were deposited to the National Center for Biotechnology Information Gene Expression Omnibus database (GSE234243) and are openly available.

Human RNAseq & small RNAseq data

Human raw data (FASTQ format) is encrypted and stored at the European Genome Phenome Archive (registered study: EGAS00001007318). This data is available upon request to the European Genome Phenome Archive. Details about how to access the human data deposited in European Genome Phenome Archive are available here: https://ega-archive.org/access/request-data/how-to-request-data/.

Proteomics data

Human and mouse proteomics data were deposited to the ProteomeXchange Consortium database (PXD043300) and are openly available.

Availability of source code

Project name: MAXOMOD
Project home page: https://github.com/imsb-uke/MAXOMOD_Pipeline
Operating system(s): Platform independent
Programming language: Python, R, Bash, Nextflow
Other requirements: DVC, Docker
License: MIT

All code for preprocessing and analyzing the data is available at: https://github.com/imsb-uke/MAXOMOD_Pipeline. The workflow consists of multiple scripts R & Python (`src/directory`) which can be executed in a dockerized environment which is provided on GitHub as well. The execution order is provided as a Data Version Control (DVC) workflow, which can be

automatically executed with DVC. All parameters are provided in the params/ directory. Further information can be found in the `README` file in the GitHub repository.

List of abbreviations

PFC: prefrontal cortex

ALS: Amyotrophic lateral sclerosis

sALS: sporadic Amyotrophic lateral sclerosis

gALS: genetic Amyotrophic lateral sclerosis

CTR: control

PBS: phosphate-buffered saline

RNAseq: RNA sequencing

FDR: false discovery rate

PCA: Principal Component Analysis

DVC: Data Version Control

Acknowledgments

We thank all the members of the Lingor, Bonn, Carapito, Schlapbach, Pasterkamp, Edbauer, and Bonetto laboratories for their contribution to the generation of the omics data and subsequent analyses. This work was performed by the research consortium “Multi-omic analysis of axono-synaptic degeneration in motoneuron disease (MAXOMOD)” funded in the scope of the E-Rare Joint Transnational Call for Proposals 2018 “Transnational research projects on hypothesis-driven use of multi-omic integrated approaches for discovery of disease causes and/or functional validation in the context of rare diseases.” granted to the labs of SB, VB, CC, RS, and PL. LCG, MP, LT, and PL were supported by the Bundesministerium für Bildung und Forschung (01GM1917A), and by the Munich Cluster for Systems Neurology (SyNergy). SH received funding from BMBF (grant 01GM2202A; STOP- FSGS and DFG, CRC1192). SO was funded by CRC1286 SP02 and KFO296 P8. RK was funded by FOR5068 P9 and FH by the M3I excellence initiative and a UKE postdoctoral stipend. SB was funded by CRC1286 SP02, CRC1192 PB8, and

PC3. We thank Tobias B. Huber for his support. The funders had no role in designing the study or generating the data/analyses in this work.

Author contributions

PL and LCG conceived the project, designed the sample collection methodology, reviewed the sample/data quality, and coordinated the acquisition of human tissue samples from the selected brain banks. SH, FH, RK, SO, and SB conceptualized the data analysis. LCG, MP, LT, LP, MG, PZ, and QZ processed postmortem brain material for multiomics experiments with the infrastructure provided by RJP, DE, VB, CC, and PL. MG and HR performed high-throughput sequencing experiments (RNAseq/mass spectrometry runs, respectively) with the infrastructure provided by CC and RS. SH, FH, RK, and SO were responsible for the bioinformatics analysis, and conceived/performed the statistical analyses. FH, SH, and LCG wrote the manuscript; PL and SB reviewed the manuscript with input from all co-authors. LCG, SH, and FH contributed equally. SB and PL contributed equally.

Competing interests

The authors declare no competing interests.

Figure Legends

Figure 1: **Overview of the bioinformatics workflow for RNAseq, small RNAseq, and proteomics data.** Methods and processing scripts are shown in orange diamonds, high-throughput technologies depicted in blue rectangles with round edges, and datasets available on disk in green rectangles with round edges, and Nextflow pipelines in red parallelograms. For the Nextflow pipelines, only a few steps are named here. The RNASeq pipeline (v3.0) and the small RNAseq smRNASeq pipeline (v1.0) were used.

Figure 2: Demonstration of overall quality of reads on transcriptome level. Mean phred quality scores, as reported by FastQC of the RNAseq data are displayed. Regions are colored according to FastQC's quality definitions (greed: good, orange: ok, red: bad). Overall all reads show a good quality.

Figure 3: Demonstration of overall quality of reads on small RNA data level. Mean phred quality scores as reported by FastQC are displayed. Regions are colored according to FastQC's quality definitions (greed: good, orange: ok, red: bad). For SOD1 and TDP43 the expected length after trimming is indicated by the gray-dotted line, for the others only the expected length of reads was provided. Overall all reads show a good quality.

Figure 4: **Verification of sex on transcriptome level.** VST-transformed *XIST* expression in human and mouse RNAseq experiments colored by Sex. *XIST* expression confirms the correct sex annotation.

Figure 5: **Verification of the transgenic animals. A**: Fraction of reads from the RNAseq experiments aligning against the human genome in the region of the *Fus*, the *Sod1,* and the *Tardbp* gene (+-200 bp) in the corresponding mouse model. Reads aligning against the human genome, confirm that the corresponding samples express the transgenic transcript correctly. **B:** Number of reads mapping to the pEGFP construct (U76561.1) using blastn[38] (v2.15.0) in C9orf72 transgenic animals.

Figure 6: **Overall quality of transformed transcriptomic data.** Histogram of VST-transformed expression values with samples on the x-axis colored by sex and condition. No strong difference between the sexes and conditions could be observed.

Figure 7: **Evaluation of batch effects (sex and condition).** Barchart of miRTrace quality checks with samples on the x-axis colored by detected RNA type. The fraction of reads that could not be assigned to any of the RNA types is not displayed. No strong difference between the sexes or conditions could be observed.

Figure 8: **Overall quality of transformed small RNAseq data.** Histogram of normalized mature miRNA expression values with samples on the x-axis colored by sex and condition. No strong difference between the sexes and conditions could be observed.

Figure 9: **Completeness of raw proteomics data.** Fraction of measured zeros in the proteomics experiments colored by Sex. No difference in the distribution between the sexes and the condition could be detected.

Figure 10: **Overall quality of transformed proteomics data.** Histogram of normalized protein abundance values with samples on the x-axis colored by sex and condition. No strong difference between the sexes and conditions could be observed.

Figure 11: **Evaluation of proteomics differential protein abundance analysis.** Calibration plots for case vs control differential protein abundance analysis to check if the p-values respect the assumptions of classical FDR control procedures. A high (close to 100%) differential concentration (in green) and a low uniformity underestimation (close to 0) are preferred.

Tables

Table 1: Summary of the cohort numbers and demographics for the human cohort

Human cohort	**Control**	**ALS**
**Subjects**	50	51
**Age at death (in years)**	75 (43–94)	67 (44–83)
**Sex (F/M)**	28 F / 22 M	16 F / 35 M
**Disease duration (years)**	-	3 (1–28)
Unprecise/unknown	-	39.2%
**Brain bank origin**		
NBB	18.0%	17.6%
Oxford BB	20.0%	27.5%
ICL MS & PD TB	38.0%	0.0%
London NDBB	24.0%	54.9%

NBB: The Netherlands Brain Bank; OBB: Oxford Brain Bank; ICL MS&PD TB: Imperial College London - Multiple Sclerosis and Parkinson's Tissue Bank; London NDBB: London Neurodegenerative Diseases Brain Bank. ALS, amyotrophic lateral sclerosis. Sex: male = M; female = F. A full description of the clinical features for the human cohort can be accessed within the supplementary data from our main publication.[12]

Table 2: Summary of the cohort numbers for the ALS mouse models

Mouse cohorts	**TDP-43**	
**Condition (WT /TG)**	10 WT	10 TG
**Sex (F/M)**	5 F / 5 M	5 F / 5 M
	**SOD1**	
**Condition (WT /TG)**	11 WT	9 TG
**Sex (F/M)**	5 F / 6 M	5 F / 4 M

	**C9orf72**	
**Condition (WT /TG)**	10 WT	10 TG
**Sex (F/M)**	6 F / 4 M	6 F / 4 M
	**FUS**	
**Condition (WT /TG)**	10 WT	10 TG
**Sex (F/M)**	5 F / 5 M	5 F / 5 M

Condition: wild type = WT; transgenic = TG. Sex: male = M; female = F.

References

1. Goutman, S. A. Diagnosis and Clinical Management of Amyotrophic Lateral Sclerosis and Other Motor Neuron Disorders. *Contin. Minneap. Minn* **23**, 1332–1359 (2017).
2. Suzuki, N., Nishiyama, A., Warita, H. & Aoki, M. Genetics of amyotrophic lateral sclerosis: seeking therapeutic targets in the era of gene therapy. *J. Hum. Genet.* **68**, 131–152 (2023).
3. Chia, R., Chiò, A. & Traynor, B. J. Novel genes associated with amyotrophic lateral sclerosis: diagnostic and clinical implications. *Lancet Neurol.* **17**, 94–102 (2018).
4. Talbott, E. O., Malek, A. M. & Lacomis, D. The epidemiology of amyotrophic lateral sclerosis. *Handb. Clin. Neurol.* **138**, 225–238 (2016).
5. Kim, G., Gautier, O., Tassoni-Tsuchida, E., Ma, X. R. & Gitler, A. D. ALS Genetics: Gains, Losses, and Implications for Future Therapies. *Neuron* **108**, 822–842 (2020).
6. Holm, A., Hansen, S. N., Klitgaard, H. & Kauppinen, S. Clinical advances of RNA therapeutics for treatment of neurological and neuromuscular diseases. *RNA Biol.* **19**, 594–608 (2022).
7. Aronica, E. *et al.* Molecular classification of amyotrophic lateral sclerosis by unsupervised clustering of gene expression in motor cortex. *Neurobiol. Dis.* **74**, 359–376 (2015).
8. Morello, G. *et al.* Integrative multi-omic analysis identifies new drivers and pathways in molecularly distinct subtypes of ALS. *Sci. Rep.* **9**, 9968 (2019).
9. Tam, O. H. *et al.* Postmortem Cortex Samples Identify Distinct Molecular Subtypes of ALS: Retrotransposon Activation, Oxidative Stress, and Activated

Glia. *Cell Rep.* **29**, 1164-1177.e5 (2019).

10. Brettschneider, J. *et al.* Stages of pTDP-43 pathology in amyotrophic lateral sclerosis. *Ann. Neurol.* **74**, 20–38 (2013).
11. Brettschneider, J. *et al.* TDP-43 pathology and neuronal loss in amyotrophic lateral sclerosis spinal cord. *Acta Neuropathol. (Berl.)* **128**, 423–437 (2014).
12. Caldi Gomes, L. *et al.* Multiomic ALS signatures highlight subclusters and sex differences suggesting the MAPK pathway as therapeutic target. *Nat. Commun.* **15**, 4893 (2024).
13. Humphrey, J. *et al.* Integrative transcriptomic analysis of the amyotrophic lateral sclerosis spinal cord implicates glial activation and suggests new risk genes. *Nat. Neurosci.* **26**, 150–162 (2023).
14. Chen, G.-B. *et al.* Integrated multi-omics analysis identifies novel risk loci for amyotrophic lateral sclerosis in the Chinese population. Preprint at https://doi.org/10.21203/rs.3.rs-3967132/v1 (2024).
15. Grima, N., Henden, L., Watson, O., Blair, I. P. & Williams, K. L. Simultaneous Isolation of High-Quality RNA and DNA From Postmortem Human Central Nervous System Tissues for Omics Studies. *J. Neuropathol. Exp. Neurol.* **81**, 135–145 (2022).
16. Morello, G., Salomone, S., D'Agata, V., Conforti, F. L. & Cavallaro, S. From Multi-Omics Approaches to Precision Medicine in Amyotrophic Lateral Sclerosis. *Front. Neurosci.* **14**, 577755 (2020).
17. Tahedl, M. *et al.* Propagation patterns in motor neuron diseases: Individual and phenotype-associated disease-burden trajectories across the UMN-LMN spectrum of MNDs. *Neurobiol. Aging* **109**, 78–87 (2022).

18. Eshima, J. *et al.* Molecular subtypes of ALS are associated with differences in patient prognosis. *Nat. Commun.* **14**, 95 (2023).

19. Ewels, P. A. *et al.* The nf-core framework for community-curated bioinformatics pipelines. *Nat. Biotechnol.* **38**, 276–278 (2020).

20. Gordon, D. *et al.* Single-copy expression of an amyotrophic lateral sclerosis-linked TDP-43 mutation (M337V) in BAC transgenic mice leads to altered stress granule dynamics and progressive motor dysfunction. *Neurobiol. Dis.* **121**, 148–162 (2019).

21. Gurney, M. E. *et al.* Motor neuron degeneration in mice that express a human Cu,Zn superoxide dismutase mutation. *Science* **264**, 1772–1775 (1994).

22. LaClair, K. D. *et al.* Congenic expression of poly-GA but not poly-PR in mice triggers selective neuron loss and interferon responses found in C9orf72 ALS. *Acta Neuropathol. (Berl.)* **140**, 121–142 (2020).

23. Mitchell, J. C. *et al.* Overexpression of human wild-type FUS causes progressive motor neuron degeneration in an age- and dose-dependent fashion. *Acta Neuropathol. (Berl.)* **125**, 273–288 (2013).

24. Tyanova, S., Temu, T. & Cox, J. The MaxQuant computational platform for mass spectrometry-based shotgun proteomics. *Nat. Protoc.* **11**, 2301–2319 (2016).

25. Andrews, S. FastQC: A Quality Control Tool for High Throughput Sequence Data. (2010).

26. Patro, R., Duggal, G., Love, M. I., Irizarry, R. A. & Kingsford, C. Salmon provides fast and bias-aware quantification of transcript expression. *Nat. Methods* **14**, 417–419 (2017).

27. Frankish, A. *et al.* GENCODE 2021. *Nucleic Acids Res.* **49**, D916–D923 (2021).

28. Kang, W. *et al.* miRTrace reveals the organismal origins of microRNA sequencing data. *Genome Biol.* **19**, 213 (2018).
29. Langmead, B., Trapnell, C., Pop, M. & Salzberg, S. L. Ultrafast and memory-efficient alignment of short DNA sequences to the human genome. *Genome Biol.* **10**, R25 (2009).
30. Danecek, P. *et al.* Twelve years of SAMtools and BCFtools. *GigaScience* **10**, giab008 (2021).
31. Griffiths-Jones, S., Grocock, R. J., van Dongen, S., Bateman, A. & Enright, A. J. miRBase: microRNA sequences, targets and gene nomenclature. *Nucleic Acids Res.* **34**, D140-144 (2006).
32. Bolstad, B. bmbolstad/preprocessCore. (2024).
33. Love, M. I., Huber, W. & Anders, S. Moderated estimation of fold change and dispersion for RNA-seq data with DESeq2. *Genome Biol.* **15**, 550 (2014).
34. Stekhoven, D. J. & Bühlmann, P. MissForest--non-parametric missing value imputation for mixed-type data. *Bioinforma. Oxf. Engl.* **28**, 112–118 (2012).
35. Ritchie, M. E. *et al.* limma powers differential expression analyses for RNA-sequencing and microarray studies. *Nucleic Acids Res.* **43**, e47 (2015).
36. Chow, J. C., Yen, Z., Ziesche, S. M. & Brown, C. J. Silencing of the mammalian X chromosome. *Annu. Rev. Genomics Hum. Genet.* **6**, 69–92 (2005).
37. Giai Gianetto, Q. *et al.* Calibration plot for proteomics: A graphical tool to visually check the assumptions underlying FDR control in quantitative experiments. *Proteomics* **16**, 29–32 (2016).
38. Altschul, S. F., Gish, W., Miller, W., Myers, E. W. & Lipman, D. J. Basic local alignment search tool. *J. Mol. Biol.* **215**, 403–410 (1990).

Click here to access/download;Figure;Fig1.pdf

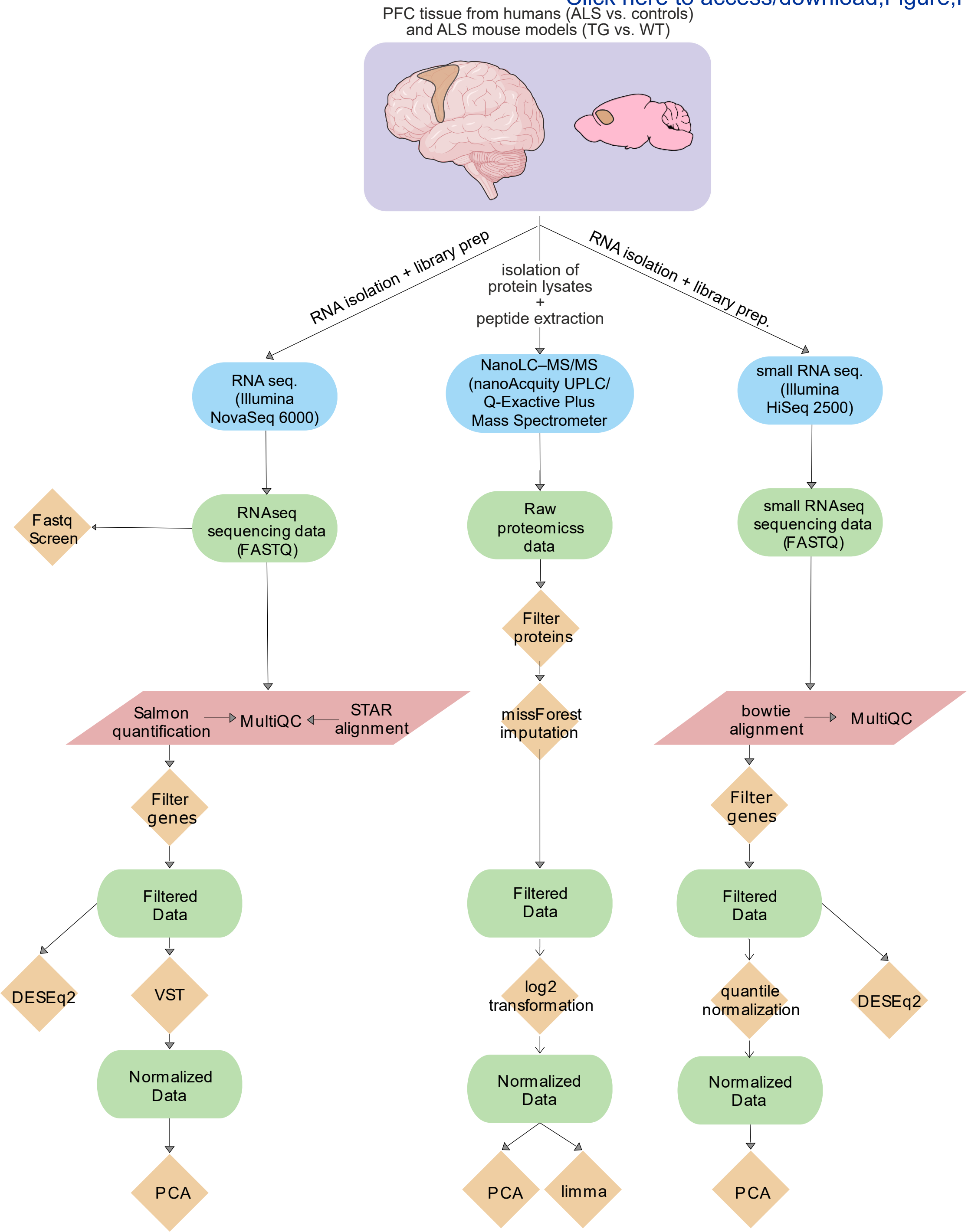

Click here to access/download;Figure;Fig2.pdf

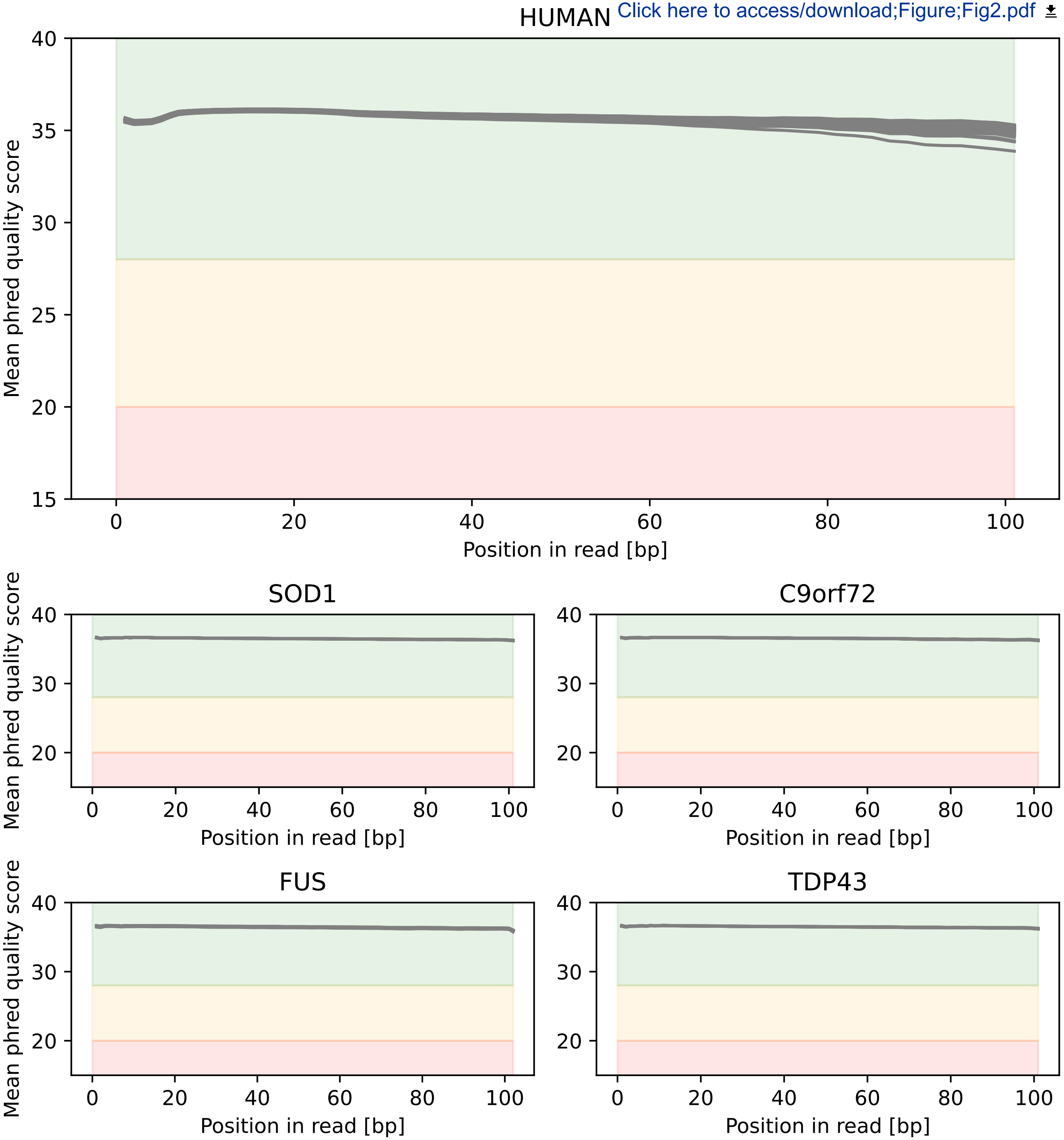

Click here to access/download;Figure;Fig3.pdf

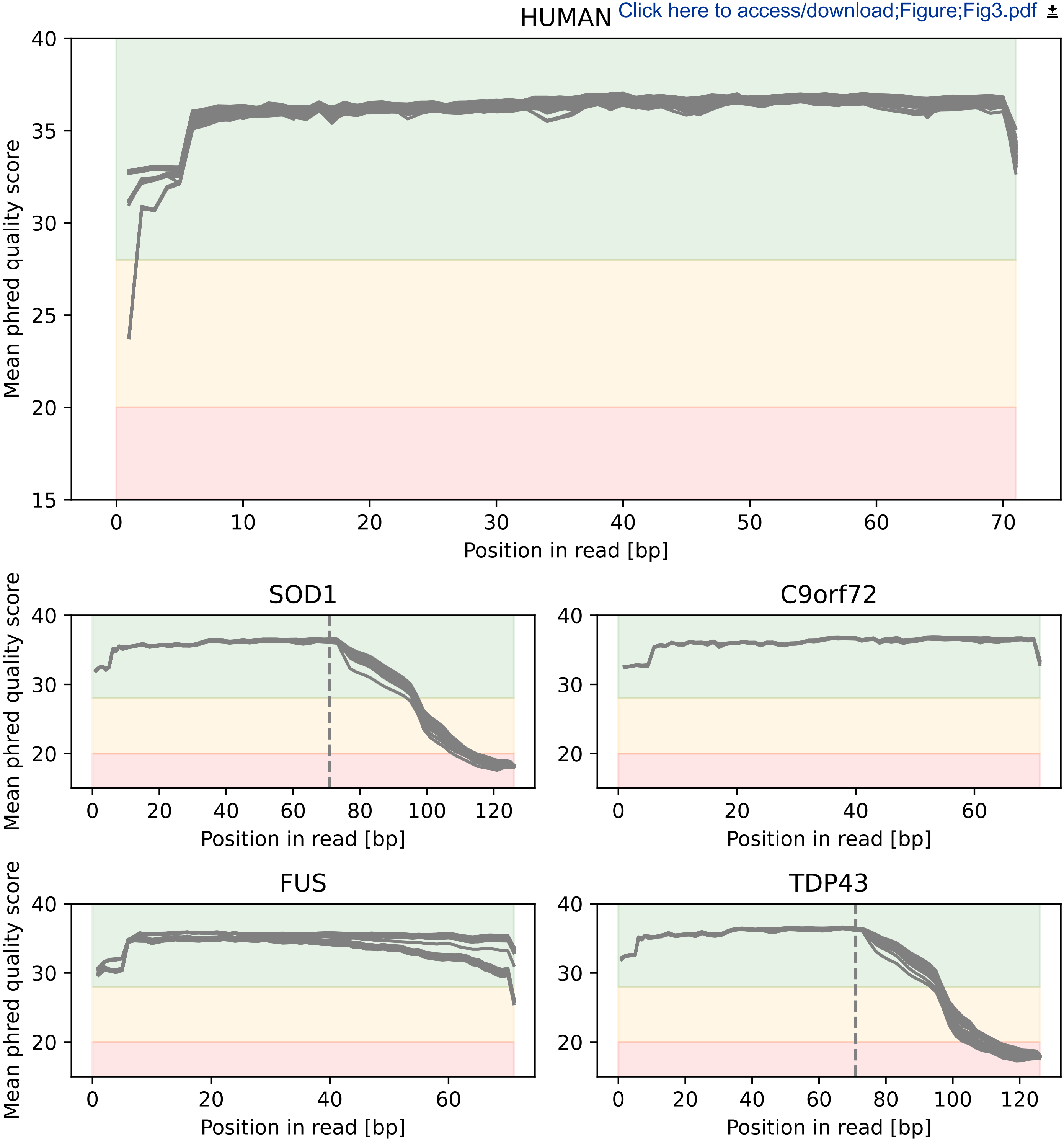

Click here to access/download;Figure;Fig4.pdf

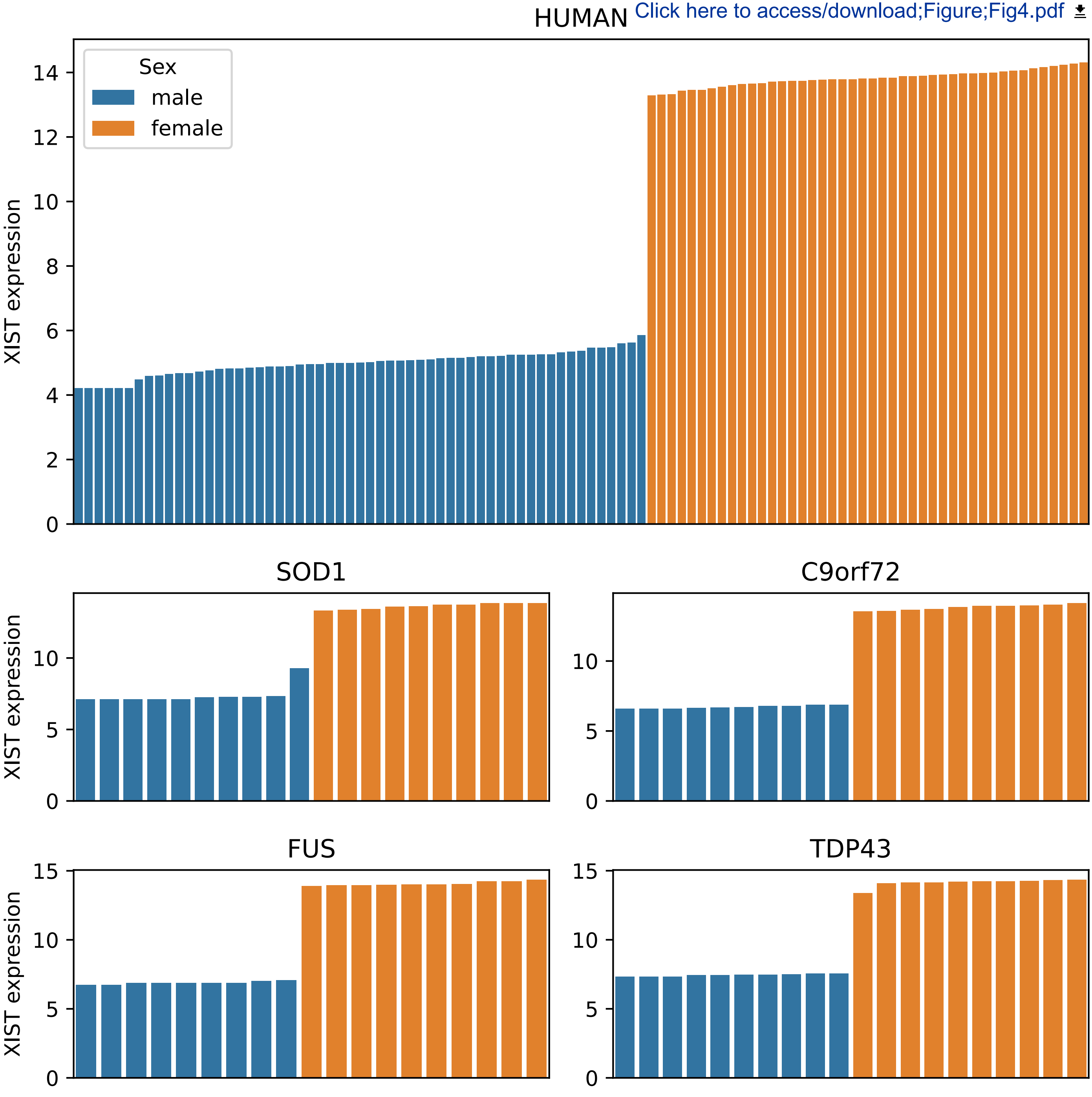

Click here to access/download;Figure;Fig5.pdf

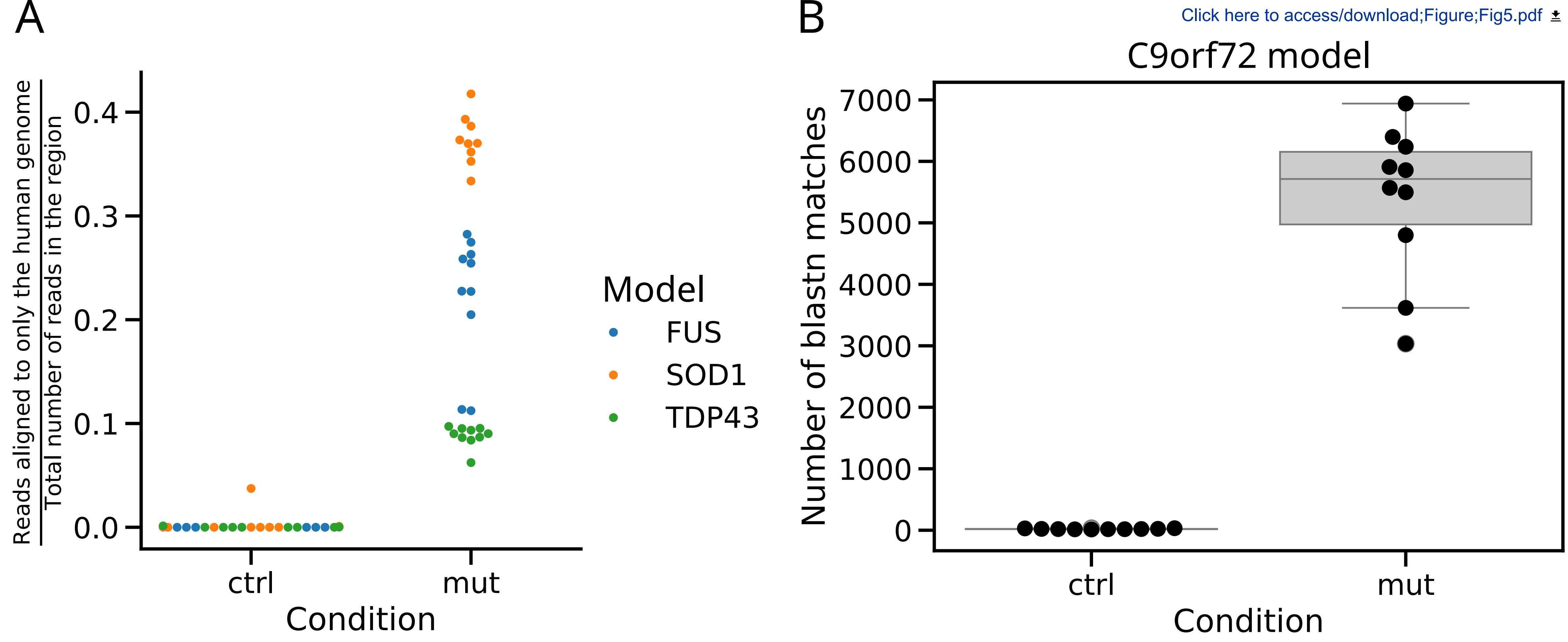

Click here to access/download;Figure;Fig6.pdf

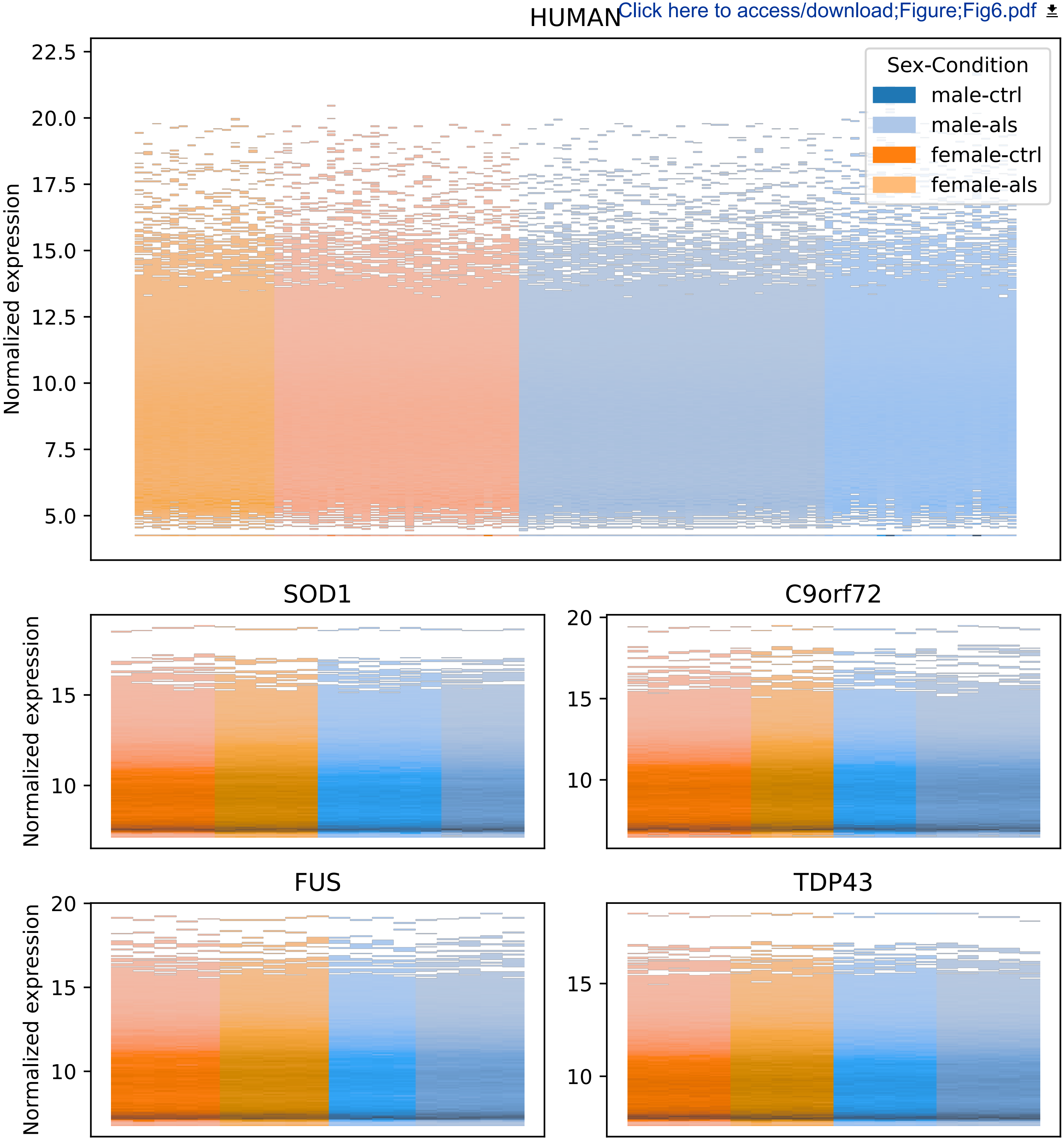

Click here to access/download;Figure;Fig7.pdf

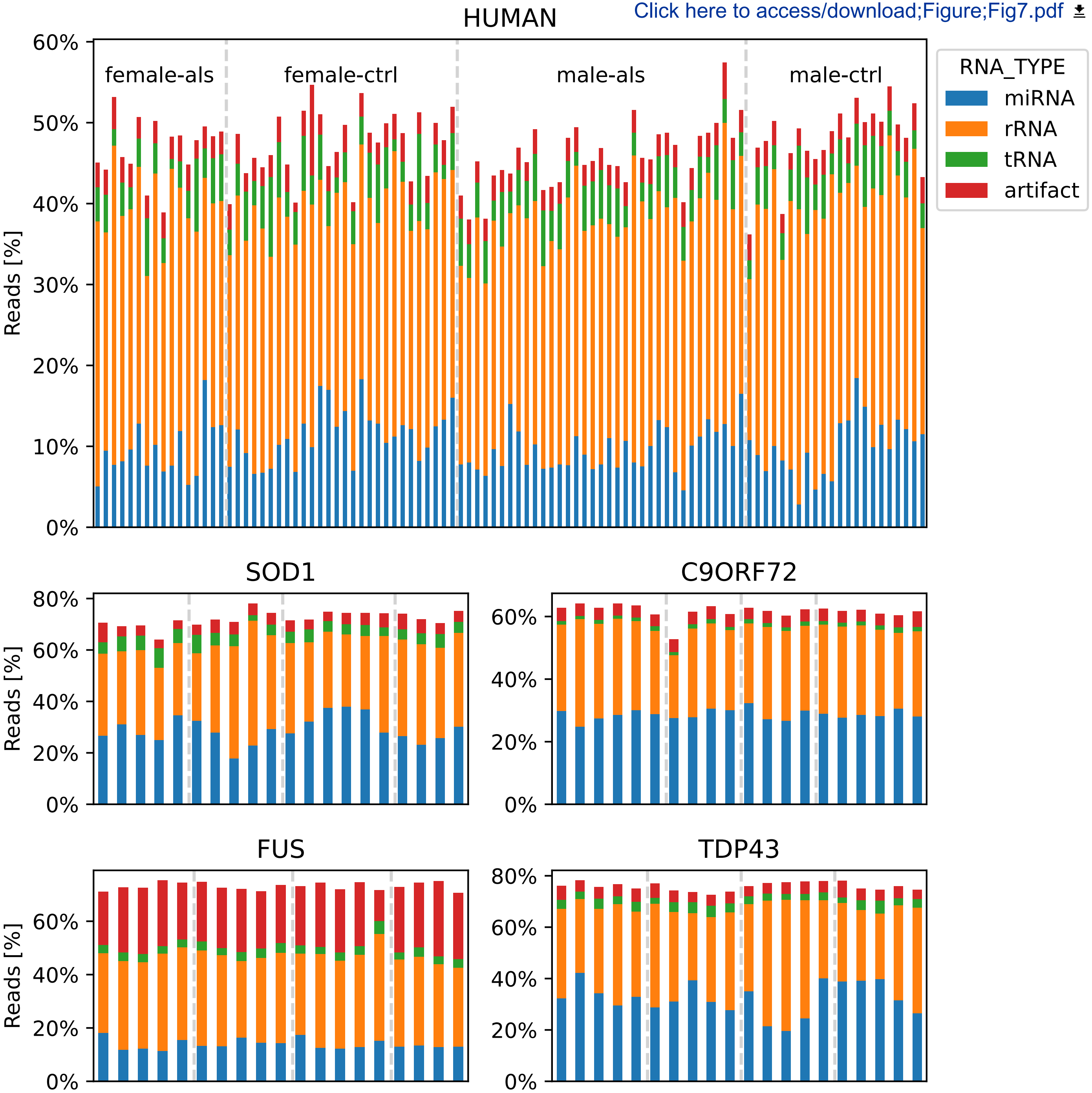

Click here to access/download;Figure;Fig8.pdf

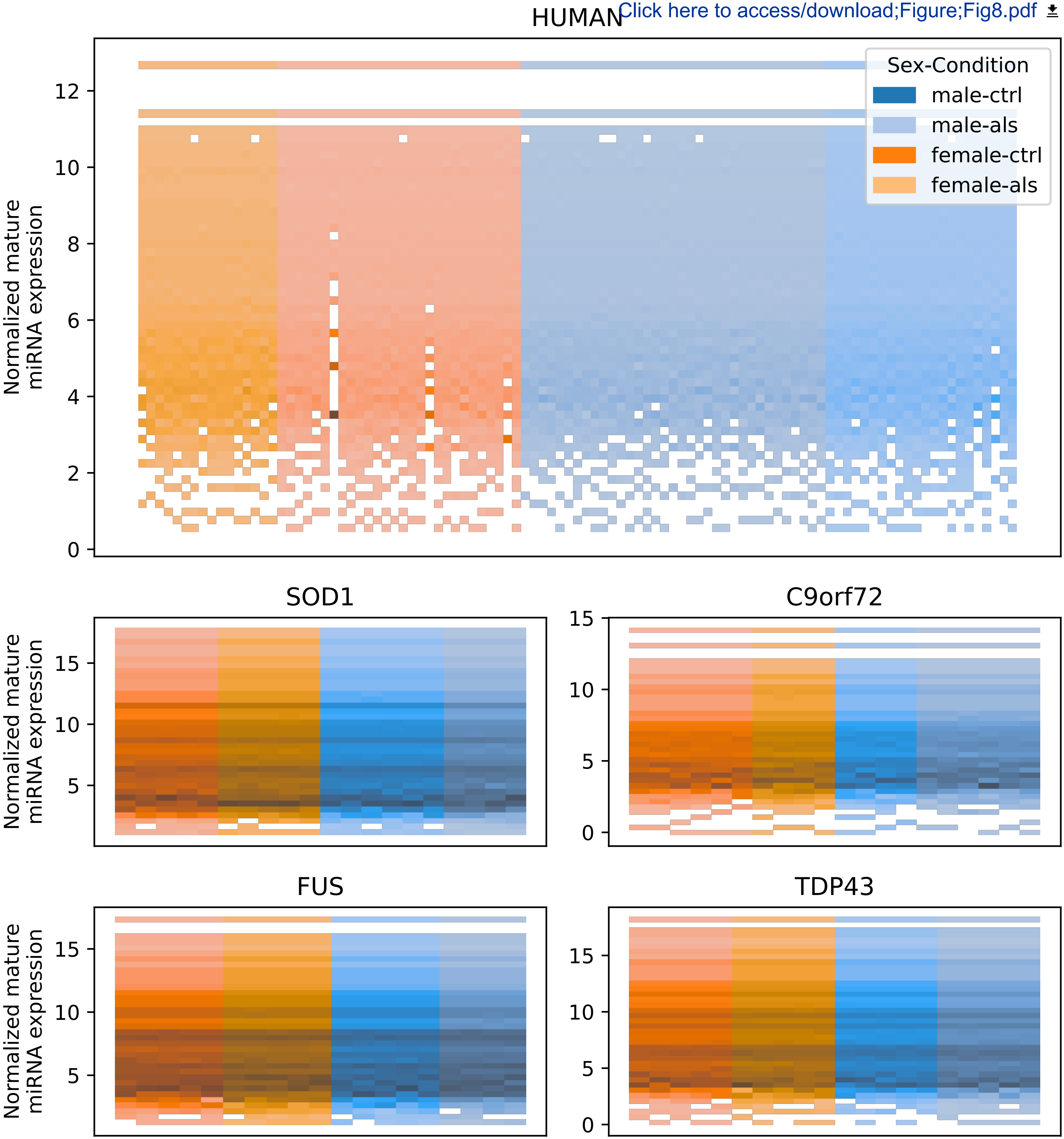

Click here to access/download;Figure;Fig9.pdf

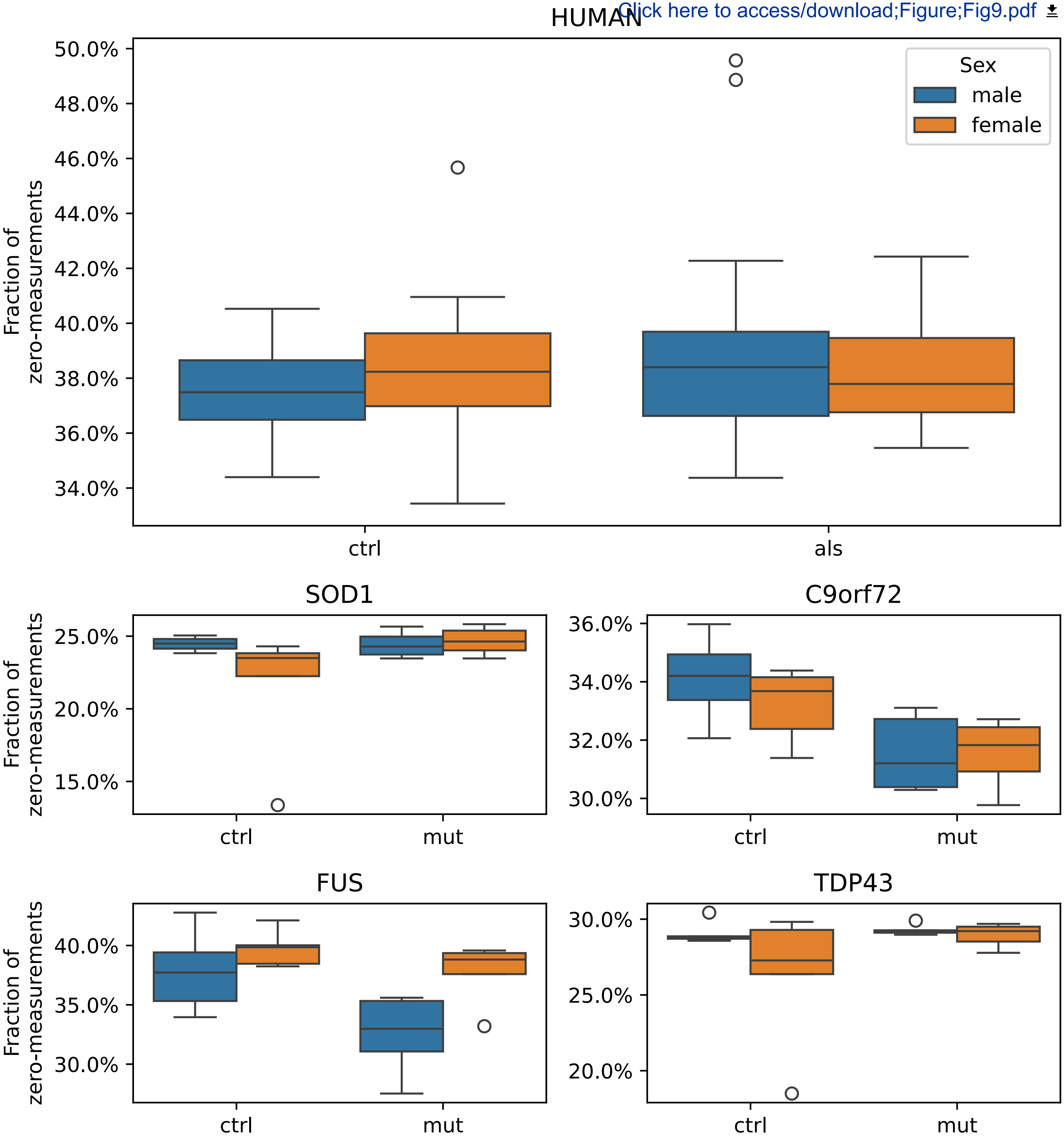

Click here to access/download;Figure;Fig10.pdf

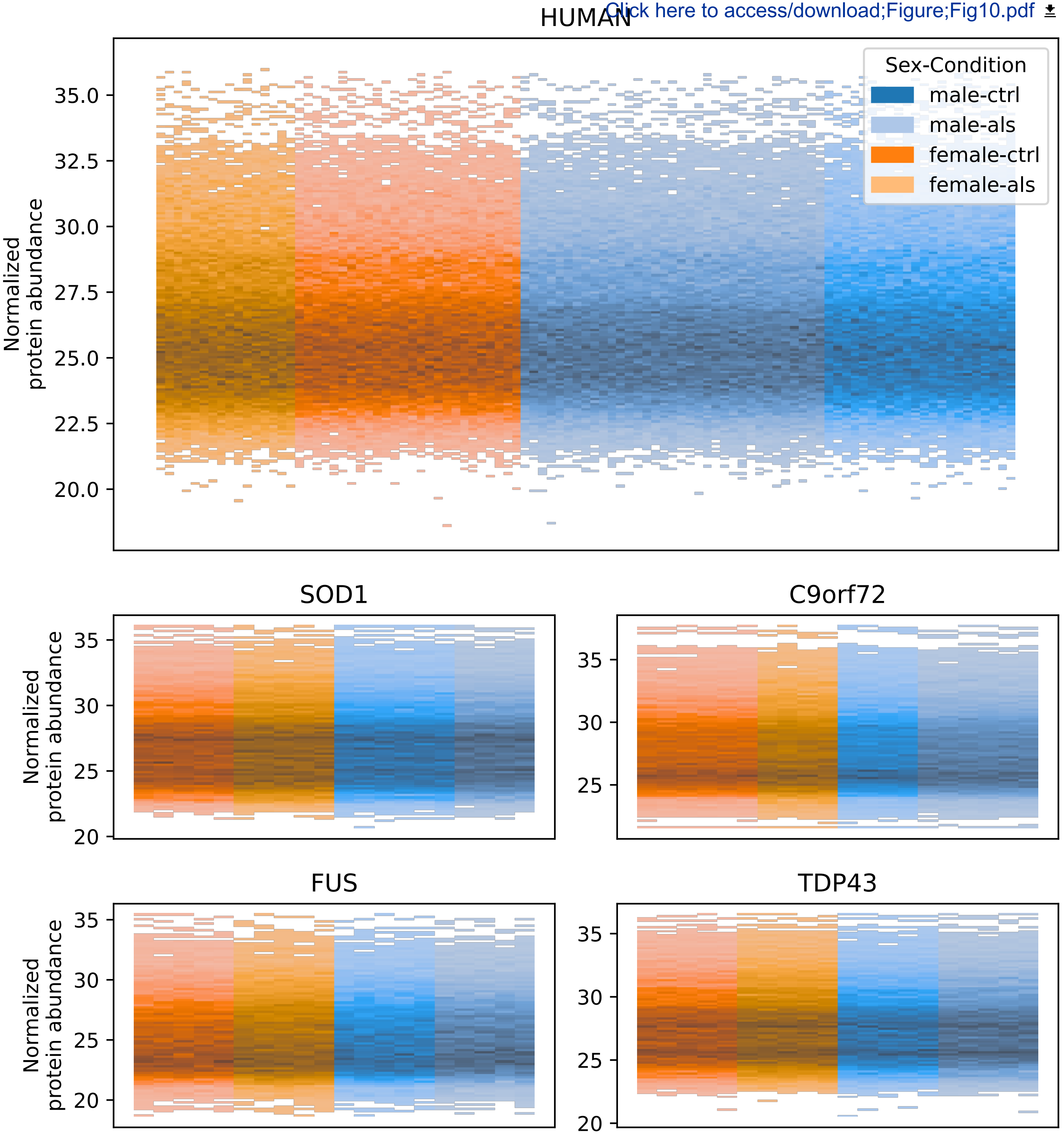

Click here to access/download;Figure;Fig11.pdf

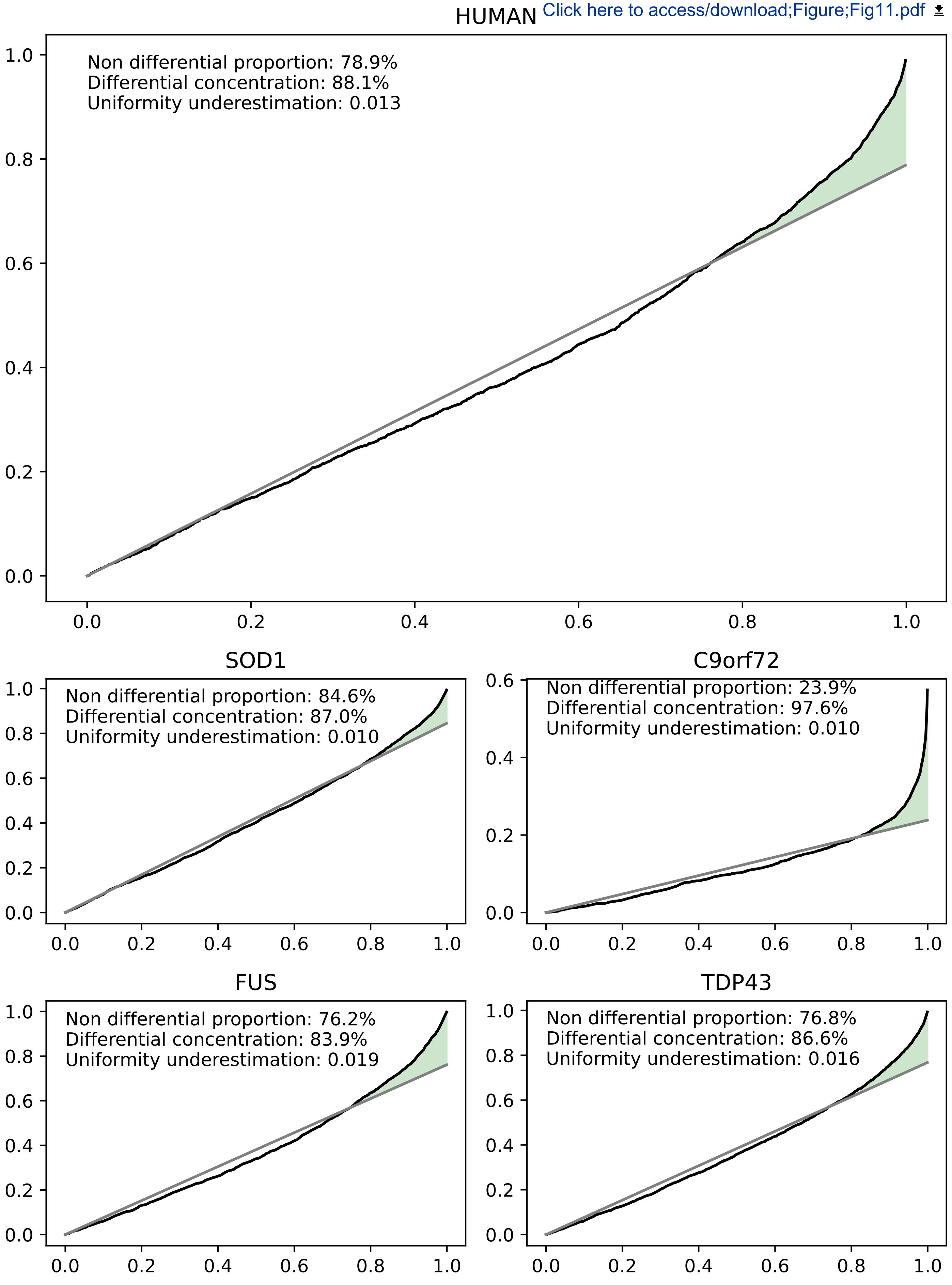

Author Cover Letter

Click here to access/download;Personal Cover;Cover Letter .docx

Klinikum rechts der Isar · Klinik für Neurologie · 81675 München

DIE DEUTSCHEN UNIVERSITÄTSKLINIKA®

Klinikum rechts der Isar

Klinikum rechts der Isar
Anstalt des öffentlichen Rechts

Klinik und Poliklinik für Neurologie
Direktor: Prof. Dr. B. Hemmer

Prof. Dr. Paul Lingor
Oberarzt

Ismaninger Straße 22
81675 München
E-Mail: paul.lingor@tum.de
Tel: 089 4140-8257
Fax: 089 4140-4867

To the Editor in Chief of *Giga Science*

Dr. Scott Edmunds

June 20th, 2024

Dear Dr. Edmunds,

We are pleased to submit our data description article for publication in *Giga Science*, as a Data Note paper. Our manuscript is titled "*A Data Set Profiling the Multi-omic Landscape of the Prefrontal Cortex in Amyotrophic Lateral Sclerosis*". The paper was co-authored by Fabian Hausmann, Lucas Caldi Gomes, Sonja Hänzelmann, Robin Khatri, Sergio Oller, Mojan Parvaz, Laura Tzeplaeff, Laura Pasetto, Marie Gebelin, Qihui Zhou, Pavol Zelina, Dieter Edbauer, R. Jeroen Pasterkamp, Hubert Rehrauer, Ralph Schlapbach, Christine Carapito, Valentina Bonetto and Stefan Bonn.

In brief, this manuscript provides a comprehensive summary of the multi-omic analyses conducted as part of our study recently published by *Nature Communications* titled "*Multiomic ALS signatures highlight subclusters and sex differences suggesting the MAPK pathway as therapeutic target*" (https://doi.org/10.1038/s41467-024-49196-y). This data description details the sources and computational workflow of the international research consortium "MAXOMOD - Multiomic analysis of axono-synaptic degeneration in motoneuron disease" funded by the E-Rare Joint Transnational Call for Proposals 2018.

In this study, we performed a detailed multi-omic analysis of the prefrontal cortex of 51 patients with sporadic amyotrophic lateral sclerosis (ALS) and 50 control subjects. Additionally, we analyzed four transgenic mouse models of C9orf72-, SOD1-, TDP43-, and FUS-ALS to characterize early and sex-specific disease mechanisms in ALS. Our research resulted in a unique data set and a reproducible and extendable computational workflow that integrates multiple omics types to understand the molecular architecture of ALS in the PFC comprehensively. Our data description manuscript includes detailed methodologies and quality control measures for RNA sequencing (mRNA and small RNA) and proteomics. This manuscript also emphasizes the importance of accessibility and reproducibility, providing extensive documentation of bioinformatics workflows and code to facilitate data reuse and transparency in analysis adhering to the FAIR (findable, accessible, interoperable, reproducible) principles.

As potential referees, we may suggest:

- Cedric Raoul, Montpellier (cedric.raoul@inserm.fr)
- Eran Hornstein, Jerusalem (eran.hornstein@weizmann.ac.il)
- Nilo Riva, Milan (nilo.riva@istituto-besta.it)
- Hans Fangohr, Hamburg (hans.fangohr@mpsd.mpg.de)
- Lorenz Adlung, Hamburg (l.adlung@uke.de)

Vorstand:
Dr. Martin Siess
(Ärztlicher Direktor, Vorsitzender)

Marie le Claire
(Kaufmännische Direktorin)

Silke Großmann
(Pflegedirektorin)

Prof. Dr. Stephanie E. Combs
(Dekanin)

Bankverbindung:
Bayer. Landesbank Girozentrale

BIC: BYLADEMM
IBAN: DE82 7005 0000 0000 0202 72
USt-IdNr. DE 129 52 3996

Technische Universität München

We believe our study makes a significant contribution to the field of ALS research. The detailed data description we present will serve as a valuable resource for your journal's readership. We are available to provide any further information or clarification needed.

This manuscript has not been published or presented elsewhere in part or in entirety and is not under consideration by another journal. The study design was approved by the appropriate ethics review board. We have read and understood your journal's policies, and we believe that neither the manuscript nor the study violates any of these. There are no conflicts of interest to declare.

We hope to now meet the high standards of *Giga Science* and look forward to hearing from you soon.

Yours sincerely,

Paul Lingor
(for all co-authors)
