# Supplementary material for: A dataset profiling the multiomic landscape of the prefrontal cortex in amyotrophic lateral sclerosis
Source: Gigascience. 2024 Dec 18;13:giae100. doi: 10.1093/gigascience/giae100 (PMC11653894; doi:10.1093/gigascience/giae100)
Supplement: giae100_GIGA-D-24-00236_Revision_1 [file giae100_giga-d-24-00236_revision_1.pdf]

GigaScience

A Data Set Profiling the Multi-omic Landscape of the Prefrontal Cortex in Amyotrophic Lateral Sclerosis

--Manuscript Draft--

Manuscript Number:	GIGA-D-24-00236R1
Full Title:	A Data Set Profiling the Multi-omic Landscape of the Prefrontal Cortex in Amyotrophic Lateral Sclerosis
Article Type:	Data Note

Funding Information:	Deutsche Forschungsgemeinschaft (CRC1286, CRC1192)	Prof Stefan Bonn
	Bundesministerium für Bildung und Forschung (MAXMOMD, 01GM1917A)	Prof Paul Lingor
	Munich Cluster for Systems Neurology	Prof Paul Lingor
	Deutsche Forschungsgemeinschaft (Immune-Mediated Glomerular Diseases – Basic Concepts and Clinical Implications, CRC1192)	Dr Sonja Hänzelmann
	Bundesministerium für Bildung und Forschung (grant 01GM2202A; STOP-FSGS)	Dr Sonja Hänzelmann

Abstract:	Amyotrophic lateral sclerosis is the most common motor neuron disease, which still lacks effective disease-modifying therapies. Similar to other neurodegenerative disorders, such as Alzheimer's and Parkinson's disease, ALS pathology is presumed to propagate over time, originating from the motor cortex and spreading to other cortical regions. Exploring early disease stages is crucial to understand the causative molecular changes underlying the pathology. For this, we sampled human postmortem prefrontal cortex (PFC) tissue from Brodmann area 6, an area that exhibits only moderate pathology at the time of death, and performed a multiomic analysis of 51 sporadic ALS patients and 50 control subjects. To compare sporadic disease to genetic ALS, we additionally analyzed PFC tissue from four transgenic ALS mouse models (C9orf72-, SOD1-, TDP-43-, and FUS-ALS) using the same methods. This multiomic data resource includes transcriptome, small RNAome and proteome data from female and male samples, aimed at elucidating early and sex-specific ALS mechanisms, biomarkers, and drug targets.

Corresponding Author:	Paul Lingor<br>Technical University of Munich: Technische Universitat Munchen<br>München, GERMANY
Corresponding Author Secondary Information:	
Corresponding Author's Institution:	Technical University of Munich: Technische Universitat Munchen
Corresponding Author's Secondary Institution:	
First Author:	Fabian Hausmann
First Author Secondary Information:	
Order of Authors:	Fabian Hausmann
	Lucas Caldi Gomes
	Sonja Hänzelmann
	Robin Khatri
	Sergio Oller
	Marie Gebelin

	Mojan Parvaz
	Laura Tzeplaeff
	Laura Pasetto
	Qihui Zhou
	Pavol Zelina
	Dieter Edbauer
	R. Jeroen Pasterkamp
	Hubert Rehrauer
	Ralph Schlapbach
	Christine Carapito
	Valentina Bonetto
	Stefan Bonn
	Paul Lingor
**Order of Authors Secondary Information:**	

Response to Reviewers:

Rebuttal Letter for GIGA-D-24-00236

A Data Set Profiling the Multi-omic Landscape of the Prefrontal Cortex in Amyotrophic Lateral Sclerosis
Fabian Hausmann; Lucas Caldi Gomes; Sonja Hänzelmann; Robin Khatri; Sergio Oller; Mojan Parvaz; Laura Tzeplaeff; Laura Pasetto; Marie Gebelin; Qihui Zhou; Pavol Zelina; Dieter Edbauer; R. Jeroen Pasterkamp; Hubert Rehrauer; Ralph Schlapbach; Christine Carapito; Valentina Bonetto; Stefan Bonn; Paul Lingor

Reviewer reports:

Reviewer #1: This "Data Note" appears to be a well-written introduction to the dataset that was used to write citation 12 (i.e. https://doi.org/10.1038/s41467-024-49196-y). In order to evaluate this Data Note I retrieved the journals stated purpose for such article types and have included the pertinent details below:
One of the aims of a Data Note is to incentivize and more rapidly release data before subsequent detailed analysis has been carried out. We do also publish Data Notes in coordination with or after the publication of an analysis paper, but we expect the Data Note to add value, especially in cases where the analysis paper has already been published. Data Notes should include significant additional detail that might not have been appropriate in the research paper, including information on data collection, detailed data validation, and information on exactly how these data can be re-used.
As the article has been published and the data made available, I would like to see the second part of the paragraph, an inclusion of significant additional detail, before publication, if that is what the authors and editor decide.

Answer: We thank the reviewer for this comment about reusability and agree that this is a very important aspect of this paper. We want to stress the point that none of the quality control and data validation procedures presented here are described in or are part of the original publication. We also present complete new datasets (e.g., open modification searches covering post translational modifications with the proteomics data). To further increase the reuse potential of the dataset we added three new sections about possible analyses and provided code to execute these, in addition to our previous section about the reuse potential.

I had a few questions related to the Data Note on its own that I would like to see addressed.

1) I applaud the authors focus on sporadic ALS. As the authors point out, a subset of

sALS patients harbor disease causing mutations citing references 2 and 3. Did you find any mutations associated with these cases? If so which and where? I'm also a little uncertain as to why the mouse models are the best method of interrogating the differences between sALS and gALS. Please expand on the utility of these specific mouse models and why you chose these models over gALS cases from the 4 brain banks.

Answer: We thank the reviewer for their thorough review and insightful comments. As explained in our original publication (Caldi Gomes & Hänzelmann et al., 2024, Nature Communications), we conducted a comprehensive genetic assessment of our ALS cohort using a gene panel analysis. This genetic screening included 30 major ALS-associated genes, and the findings indicated that the cohort predominantly consists of sporadic cases: only 2 out of the 51 ALS cases analyzed presented genetic alterations (one C9orf72 repeat expansion and one pathogenic variant of NEK1 [c.3107C>G, p.Ser1036Ter] were detected).

As there are currently no mouse models available that replicate sporadic ALS, we selected transgenic SOD1, TDP43, C9orf72, and FUS mouse models based on the importance and prevalence of these causative mutations in ALS. These models represent the most common ALS-causing mutations and provide a broad spectrum of disease mechanisms to study. Our choice was also influenced by the need to utilize well-characterized models that offer reproducibility and a wealth of existing data for comparison. The SOD1-G93A mouse model, for example, is the most-commonly used mouse model for ALS and provides a valuable benchmark for comparative studies.

Although sALS and gALS present differences in their etiology, both exhibit common key pathological features. These include, for example, mitochondrial dysfunction, neuroinflammation, and protein aggregation (PMID: 28871262, PMID: 32854276, PMID: 27830784). Indeed, up to 97% of all ALS patients (including sALS and gALS) demonstrate TDP43 pathology that can be reproduced in transgenic mice (https://doi.org/10.2217/fnl.10.47 ; PMID: 37638324; PMID: 32854276). While the selected models are based on specific genetic mutations often associated with familial ALS, they are very valuable for studying the broader mechanisms that are also relevant to sALS. All four models used here are known to exhibit key pathological features shared between gALS and sALS. In this way, they serve as powerful tools for investigating the underlying mechanisms contributing to motoneuron degeneration, regardless of whether the ALS is of genetic or sporadic form.

Thus, studying these models can provide insights that apply to both forms of the disease. Our data strongly suggests that each of the genetic mouse models used, represents a subgroup of sporadic ALS patients. In conclusion, although the mouse models employed here are based on genetic mutations, their ability to elucidate shared pathogenic pathways in ALS justified their use in our study of sporadic ALS.

2) I am also a little dubious as to the justification of PFC as having signs of early alterations that are absent in other brain regions post mortem. I would like to see additional evidence that the authors may not have had space to include in the original publication.

Answer: We appreciate the reviewer's request for further clarification regarding our focus on the prefrontal cortex (PFC) in ALS. Our decision to examine the PFC (particularly Brodmann area 6) is based on both pathological staging and the search for early disease-related alterations that may be less apparent in regions more severely affected by ALS at the time of death. Moreover, post-mortem motor cortex and spinal cord have already been extensively studied and described in the disease, but as most-severely affected regions are strongly biased by end-stage alterations.

As extensively characterized by Brettschneider et al. (2013) (PMID: 23686809), the spread of TDP-43 inclusions follows a distinct pattern: it shows a rostrocaudal progression, starting in the motor cortex and subsequently spreading to other cortical and subcortical areas. By the end stage of ALS, the motor cortex shows extensive degeneration and gliosis, which obscures the identification of early molecular changes due to the overwhelming presence of advanced and rather unspecific pathology.
With our multiomics studies in the PFC, we aimed to circumvent the description of end-

stage pathology. Instead, we tried to capture molecular changes that occur before widespread neuronal death/associated cellular and tissue changes, providing an overview of earlier disease mechanisms. Being affected only later in the disease course, the PFC is deemed to exhibit only intermediate levels of TDP-43 pathology at the time of death, possibly harboring early signs of disease-related alterations that are not as evident in more severely affected brain regions. It is worth noting that the findings of Brettschneider et al. (2013) have been consistently supported by subsequent studies, reinforcing the understanding that the prefrontal cortex is affected later in the disease course.

As requested by the reviewer, we present an overview of some of the studies that provide further evidence for the findings described in Brettschenider et al:
A study by Kassubek et al. (2014) used diffusion tensor imaging to demonstrate that ALS-related axonal damage in white-matter tracts affected the same regions as reported in the TDP-43 staging study of Brettschneider et al (PMID: 24736303). Moreover, a recent study using an ALS mouse model demonstrated that TDP-43-mediated pathology spreads through corticofugal tracts affected in ALS (PMID: 37011806). Another study investigated the TDP-43 spreading pattern in transgenic mice, showing that injecting TDP-43 preformed fibrils (PFFs) into the motor cortex resulted in the prion-like spread of TDP-43 pathology along the pyramidal tract. Over time, this led to widespread TDP-43 pathology throughout various brain regions, confirming the prion-like transmission of TDP-43 in ALS (PMID: 33461623).
Finally, a recent perspective paper (PMID: 32157757) compiled evidence from several studies that reinforce the anatomical spreading and propagation hypothesis of TDP-43 pathology in ALS-affected patients, as proposed by Brettschneider et al. Overall, these studies provide further evidence that the prefrontal cortex (PFC) is affected only later in the disease course, offering insights into the early mechanisms of ALS and potential intervention points.

In summary, by focusing on the PFC, we aim to uncover molecular changes that represent early, pre-symptomatic stages of ALS. Understanding these changes could provide critical insights into the initial pathogenic events that precede widespread neurodegeneration.

3) In the data preparation please include the number of files, fractionation, number of peptides, and total number of proteins and or transcripts.

Answer: As requested by the reviewer, we added in the data preparation section in more detail the number of detected transcripts, sRNAs and detected proteins (Table 3 and below). The sequencing experiments resulted in one FASTQ file per sample, e.g. 20 FASTQ files for SOD1 transcriptomics data.

ModelTranscriptomicsProteomicssRNA (mature/hairpin)
Human196412363736 (224/512)
SOD1 - Mouse165832854893 (526/367)
TDP43 - Mouse168012802907 (534/373)
C9orf72 - Mouse174652866754 (271/483)
FUS - Mouse172302522812 (468/344)

4) I would like to see additional searches for modifications to round out the inclusion of additional information about this dataset (for proteomics) that may not have fit in the original paper.

Answer:
Thank you for the suggestion. We performed an open modification search using ionbot and the results are available at 10.6084/m9.figshare.27108562 for re-use by other researchers. In addition, we added the newly created Figure 12 (shown below) as an overview of the results of the open modification search in the main manuscript.
Figure legend: Top modifications found by the open modification search using ionbot for the four mouse models and human samples. For each model, the top 10 modifications were selected and the number of occurrences of the union of those (17 modifications) is displayed. Fixed modifications (Carbamidomethyl, Oxidation, Acetyl[N-term]) and sequence variations (Glu->Ser, Arg->Orn, Ser->Ala, Gln->pyro-

Powered by Editorial Manager® and ProduXion Manager® from Aries Systems Corporation

Glu, Xle->Pro, Tyr->Phe, Delta:H(2)C(2)[N-term]) were removed for display.

The following methods description was added the methods part of the manuscript:

“In addition, an open modification search was performed. MGF files from the mouse and human proteomics data were loaded into IonBot (https://ionbot.cloud/) software (v. 0.11.0). Provided databases were used, either human (9606 entries) or mus musculus (10090 entries), with a K|R cleavage pattern. Error tolerances were set on default values: MS precursor tolerance at 20 ppm and MS/MS fragment tolerance at 0.02 Da. Methionine oxidation and protein N-term acetylation were set as variable modifications, while cysteine carbamidomethylation as a fixed modification. Open modification search option was enabled.”

Well done getting this information together, it was clearly a lot of work. Following major revisions I would be interested in seeing this note again.

Reviewer #2:
Major comments:
The authors have presented a useful omics resource for ALS studies, which could be used to identify biomarkers and drug targets for ALS treatments,. However, the following points should be addressed.

1. Although the authors have described differential expression and enrichment analyses, as well as proteomics data analysis in their methods section, they did not report any of these results in the manuscript. Including these findings is crucial for providing a comprehensive understanding of the data and its implications. The absence of these results limits the ability of readers to fully assess the scope and impact of the analyses performed. I recommend that the authors include detailed results from these analyses to enhance the manuscript's completeness and clarity.

Answer: We thank the reviewer for the constructive suggestions. We would like to clarify that the original multiomics data, as well as the functional interpretation for all datasets and models, have been extensively detailed in our prior publication in Nature Communications (https://doi.org/10.1038/s41467-024-49196-y). With our submission of this Data Note manuscript to GigaScience, we aimed at providing better documentation, improving data accessibility and reuse for this comprehensive set of data. We believe that revisiting these findings, describing yet again the main results to draw functional conclusions might lead to redundancy and detract from the primary aim of a data note article.

To improve clarity, we decided to provide a summary of our main results here, and also included a new paragraph for this purpose in our Context section for the current submission. In brief, in our initial study, we conducted a multiomic analysis of PFC tissue, revealing distinct molecular subclusters within ALS patients. These subclusters showed varying patterns in gene, protein and miRNA expression, suggesting the presence of different underlying disease mechanisms. These findings underscore the need for personalized therapeutic approaches. Another important aspect of our study was the identification of pronounced sex differences captured in the molecular profiles of ALS patients, with male patients exhibiting more pronounced alterations overall.

Our study emphasized and focused on the MAPK pathway as a critical therapeutic target. The involvement of this pathway suggests it could be a focal point for developing targeted treatments, which could improve the prognosis for ALS patients. Other important pathways identified in our study were the activation of immune response, extracellular matrix composition, mitochondrial function, and RNA processing.

In parallel, we have analyzed four different mouse models, bearing the most common ALS-causing mutations. The results from human analyses were corroborated in ALS mouse models, which exhibited similar molecular patterns, and partially resembled the human subclusters revealed through the analysis of human brain tissue. This cross-species validation strengthens the relevance of the identified subclusters and pathways as potential therapeutic targets.

Powered by Editorial Manager® and ProduXion Manager® from Aries Systems Corporation

The findings summarized here were validated across multiple datasets, reinforcing the significance of the identified molecular sub clusters and pathways. This validation suggests that future ALS research should consider these frequently reported molecular differences, and focus on developing personalized medicine approaches, tailored to specific patient subgroups.

We hope that this clarifies the points put forward by the reviewer, as well as the intent and scope of the present manuscript.

2. The authors employed DESeq2 for identifying differentially expressed genes (DEGs) and miRNAs between experimental conditions (ALS vs. controls). While DESeq2 is a robust tool for differential expression analysis, it has limitations, particularly in handling low-count genes and in estimating dispersion in small sample sizes.

I would suggest the authors perform DEGs using the limma/voom approach. Limma, with its voom transformation, can provide better performance in terms of handling heteroscedasticity and is particularly effective for RNA-Seq data with varied library sizes. It also offers more accurate estimation of mean-variance relationships, which can improve the detection of true DEGs.

Additionally, using limma/voom can complement DESeq2 results and provide a more comprehensive analysis by cross-validating the findings. This dual approach would enhance the robustness and reliability of the differential expression analysis, potentially uncovering more biologically relevant insights.

Answer: We thank the reviewer for these suggestions. While limma/voom have been adapted for RNA-Seq data, it is important to highlight that limma was originally developed for microarray data. The voom transformation referred by the reviewer allows limma to handle RNA-Seq data by converting raw counts into log2 counts per million (log-CPM). However, this additional layer of modeling may not always capture the unique characteristics of RNA-Seq data as effectively as DESeq2. Limma-voom was originally only tested against the previous version, DESeq, and not against DESeq2. Furthermore, in a recent benchmark by Li et al. (2022) [PMID: 36112652] DESeq2 remains the recommended method for most use cases with higher sample sizes (>6).
DESeq2, on the other hand, implements independent filtering and shrinkage estimation of dispersion, which makes it particularly robust in handling low-count genes — a common challenge in RNA-Seq data analysis. These features help minimize some false positives, ensuring more accurate results for low-count genes. Moreover, DESeq2’s robust statistical framework, including its use of a negative binomial distribution, accounts for variability between experimental conditions. This makes DESeq2 especially well-suited for studies with expected variability, such as those comparing ALS patients to controls.
As requested by the reviewer, we have performed comparative analyses for both methods and the human samples and found that, in general, DESeq2 detected more differentially expressed genes than limma/voom - especially for males, which encompassed the most changes captured in our datasets (Venn diagrams). This is also the case for our proteomics samples (379 DEPs in males vs 251 DEPs in females) where we didn’t use DESeq2 but limma (without voom) for our analysis.
While it can be valuable to use limma/voom as a complementary approach to validate DESeq2 findings, in our view, DESeq2 should remain the primary method due to its direct modeling of RNA-Seq data, and its handling of biological variability, low-count genes, the high detection rate for exploratory research and its consistency with the proteomics data. Employing both methods could enhance the robustness of the analysis, but DESeq2's approach is likely to yield more comprehensive primary results, particularly in our context.

3. The authors should provide a detailed description of how they adjusted for potential confounding variables in their DEGs and proteomics analyses analysis. Specifically, it is important to describe the methods used to account for batch effects, principal components of gene/protein expression, and the effects of age and sex on their analyses. Proper adjustment for these variables is crucial for ensuring the validity and reliability of the results.

Powered by Editorial Manager® and ProduXion Manager® from Aries Systems Corporation

	Answer: We used PCA and a sample distance heatmap to check for batch effects in our transcriptomics and proteomics analyses. We could not detect any batch effects in our proteomics and transcriptomics data and exemplify this in the transcriptomics data below. We specifically investigated batch effects related to the factors brain bank, sex, case/control condition, as well as age at death. However, we identified marked sex-related differences (Figure 1b of Caldi Gomes, Hänzelmann et al., 2024; shown below) and therefore, all analyses were performed separately for each sex.<br><br>For the other factors, we found no evidence that they influenced the results (Supplementary Figure 1 of Caldi Gomes, Hänzelmann et al., 2024; shown below). For instance, if batch effects had been present, they would likely have caused distinct clustering or separation of samples. However, our analysis showed no such patterns, indicating that these factors did not introduce systematic bias to our data.<br><br>Furthermore, we could not detect any influence of the post-mortem interval on the expression of known neuronal cell death markers, indicating measurable influence by the sample handling (Supplementary Figure 4a of Caldi Gomes, Hänzelmann et al., 2024; shown below).<br>We now discuss this in a newly added section of the manuscript and added Figure 13 showing all known factors in the PCA representation for the transcriptomic (A) and proteomics (B) data.<br><br>Minor comments:<br>1. The authors should describe whether their omics data consist of single-end or paired-end reads.<br><br>Answer: Single-end reads. We made sure that this information is more explicitly stated in the methods.<br><br>2. The color scheme in Figure 6 makes it difficult to differentiate between cases and controls, particularly male-ctrl and male-als. I recommend that the authors revise the color scheme to improve contrast and distinguishability.<br><br>Answer: We thank the reviewer for the comment and agree with the color choice change. We resorted to a color-blind-friendly palette now and adjusted the respective figures.<br><br><br>--<br>Please also take a moment to check our website at https://www.editorialmanager.com/giga/l.asp?i=191766&l=V4LNH38F for any additional comments that were saved as attachments. Please note that as GigaScience has a policy of open peer review, you will be able to see the names of the reviewers.<br><br>No additional files for our submission.
**Additional Information:**	
**Question**	**Response**
Are you submitting this manuscript to a special series or article collection?	No
**Experimental design and statistics**<br><br><br>Full details of the experimental design and statistical methods used should be given in the Methods section, as detailed in our Minimum Standards Reporting Checklist. Information essential to interpreting the data presented should be made available	Yes

in the figure legends.<br><br>Have you included all the information requested in your manuscript?	
**Resources**<br><br>A description of all resources used, including antibodies, cell lines, animals and software tools, with enough information to allow them to be uniquely identified, should be included in the Methods section. Authors are strongly encouraged to cite Research Resource Identifiers (RRIDs) for antibodies, model organisms and tools, where possible.<br><br>Have you included the information requested as detailed in our Minimum Standards Reporting Checklist?	Yes
**Availability of data and materials**<br><br>All datasets and code on which the conclusions of the paper rely must be either included in your submission or deposited in publicly available repositories (where available and ethically appropriate), referencing such data using a unique identifier in the references and in the "Availability of Data and Materials" section of your manuscript.<br><br>Have you have met the above requirement as detailed in our Minimum Standards Reporting Checklist?	Yes

Powered by Editorial Manager® and ProduXion Manager® from Aries Systems Corporation

Manuscript

Click here to access/download;Manuscript;Hausmann11.11.24.docx

A Data Set Profiling the Multi-omic Landscape of the Prefrontal Cortex in Amyotrophic Lateral Sclerosis

Fabian Hausmann[1*], Lucas Caldi Gomes[3*], Sonja Hänzelmann[1,4*], Robin Khatri[1], Sergio Oller[1], Marie Gebelin[6], Mojan Parvaz[3], Laura Tzeplaeff[3], Laura Pasetto[5], Qihui Zhou[8,9], Pavol Zelina[10], Dieter Edbauer[8,9], R. Jeroen Pasterkamp[10], Hubert Rehrauer[7], Ralph Schlapbach[7], Christine Carapito[6], Valentina Bonetto[5], Stefan Bonn[1,2§], Paul Lingor[3,8,9§,#]

1 Institute of Medical Systems Biology, Center for Biomedical AI (bAIome), Center for Molecular Neuroscience (ZMNH), University Medical Center Hamburg-Eppendorf, 20251 Hamburg, Germany

2 Hamburg Center for Translational Immunology (HCTI), University Medical Center Hamburg-Eppendorf, Hamburg 20251, Germany

3 Technical University of Munich, School of Medicine, rechts der Isar Hospital, Clinical Department of Neurology, Munich, Germany.

4 III Department of Medicine, University Medical Center Hamburg-Eppendorf, Hamburg, Germany.

5 Research Center for ALS, Istituto di Ricerche Farmacologiche Mario Negri IRCCS, Milan, Italy

6 Laboratoire de Spectrométrie de Masse Bio-Organique, Université de Strasbourg, Infrastructure Nationale de Protéomique, Strasbourg, France.

7 Functional Genomics Center Zürich, ETH Zürich and University of Zürich, Zürich, Switzerland.

8 German Center for Neurodegenerative Diseases (DZNE), München, Germany.

9 Munich Cluster for Systems Neurology (SyNergy), Munich, Germany.

10 Department of Translational Neuroscience, University Medical Center Utrecht, Utrecht University, Utrecht, The Netherlands.

*,[§] These authors contributed equally.

[#]Corresponding Author:

Paul Lingor; email: paul.lingor@tum.de; Clinical Department of Neurology, School of Medicine, rechts der Isar Hospital, Technical University of Munich, Ismaninger Straße 22, 81675 Munich, Germany, Tel.: +498941408257

ORCID iDs:

Fabian Hausmann [0000-0001-6110-5824]; Lucas Caldi Gomes [0000-0003-4959-2169]; Sonja Hänzelmann [0000-0003-4953-0101]; Robin Khatri [0009-0006-5311-1718]; Sergio Oller [0000-0002-8994-1549]; Mojan Parvaz [0000-0002-0644-5559]; Laura Tzeplaeff [0009-0003-7643-3232]; Laura Pasetto [0000-0003-0319-4448]; Marie Gebelin [0009-0001-4696-3799]; Qihui Zhou [0000-0001-5733-9820]; Pavol Zelina [0009-0008-6776-663X]; Dieter Edbauer [0000-0002-7186-4653]; R Jeroen Pasterkamp [0000-0003-1631-6440]; Hubert Rehrauer [0000-0001-7612-9394]; Ralph Schlapbach [0000-0002-7488-4262]; Christine Carapito [0000-0002-0079-319X]; Valentina Bonetto [0000-0003-0456-2054]; Stefan Bonn [0000-0003-4366-5662]; Paul Lingor [0000-0001-9362-7096];

Abstract

Amyotrophic lateral sclerosis is the most common motor neuron disease, which still lacks effective disease-modifying therapies. Similar to other neurodegenerative disorders, such as Alzheimer's and Parkinson's disease, ALS pathology is presumed to propagate over time, originating from the motor cortex and spreading to other cortical regions. Exploring early disease stages is crucial to understand the causative molecular changes underlying the pathology. For this, we sampled human postmortem prefrontal cortex (PFC) tissue from Brodmann area 6, an area that exhibits only moderate pathology at the time of death, and performed a multiomic analysis of 51 sporadic ALS patients and 50 control subjects. To compare sporadic disease to genetic ALS, we additionally analyzed PFC tissue from four transgenic ALS mouse models (C9orf72-, SOD1-, TDP-43-, and FUS-ALS) using the same methods. This multiomic data resource includes transcriptome, small RNAome and proteome data from female and male samples, aimed at elucidating early and sex-specific ALS mechanisms, biomarkers, and drug targets.

Keywords: Amyotrophic lateral sclerosis; multi-omics analysis; neurodegeneration; prefrontal cortex; early disease mechanisms

Context

Amyotrophic lateral sclerosis (ALS) is a devastating motor neuron disease characterized by progressive paralysis and a shortened lifespan following symptom onset[1]. While the majority of ALS cases are sporadic (sALS) and lack a clear genetic predisposition, approximately 10% are associated with known genetic mutations (gALS)[2]. Among the most common genetic causes are mutations in the genes of *C9orf72*, *SOD1*, *TARDBP*, and *FUS.* Interestingly, a subset of sALS patients also harbor disease-causing mutations[2-4]. Despite considerable research efforts, the exact etiology of sALS remains elusive, and effective disease-modifying treatments are currently unavailable[1,5,6]. Understanding the early mechanisms of ALS pathology is paramount for identifying diagnostic biomarkers and uncovering more effective therapeutic targets. Many investigations into ALS pathology have focused on end-stage disease by using postmortem CNS tissue, which may obscure insights into earlier disease mechanisms that could offer more promising therapeutic avenues[7-9]. In contrast to the motor cortex, which is affected early in the disease and therefore often shows end-stage alterations at the death of the patients[7], the prefrontal cortex (PFC) is affected only later in the disease and thus presents a unique opportunity to explore earlier ALS pathology[10,11]. Histological studies have revealed that while the motor cortex exhibits severe pathology in later stages of the disease[7–9], the PFC demonstrates intermediate TDP-43 pathology, suggesting its relevance in elucidating earlier disease-mediated alterations[10,12]. A recent study employed multiomics to profile the molecular alterations in the spinal cord, another region heavily affected in ALS[13]. Other studies employed multiomic strategies in postmortem tissue from ALS patients, but they included only a limited number of techniques, focusing on transcriptome- or genome-based technologies[14,15]. Studies focusing on early alterations in ALS-affected brains in a comprehensive multiomic setting are still lacking[16].

In this context, the availability of omics datasets and robust analytical workflows is critical for advancing ALS research. Building upon our previous work[12], with this *Data Note*, we improved the accessibility of raw and processed data, alongside detailed descriptions of bioinformatics methodologies, as well as new data resources based on the original multiomic data. This includes extensive

documentation of bioinformatics workflows and the provision of code to facilitate reproducibility and transparency in data analysis.

We provide a broad multi-omic high-throughput sequencing data set of a cohort of 101 human samples from four different brain banks (*N* = 51 patients with sporadic ALS; *N* = 50 control subjects, males and females). The omic layers encompass mRNAomics, small RNAomics, and proteomics. Additionally, we provide corresponding data for four distinct ALS mouse models based on mutations in the genes of *SOD1*, *C9orf72*, *FUS*, and *TARDBP*. Each mouse model includes both male and female samples, with transgenic and wild-type groups represented in each omic layer (cohort numbers balanced for sex and condition) ensuring a robust comparative analysis across species. Human PFC samples were provided by four different European brain banks (London Neurodegenerative Diseases Brain Bank, the Imperial College London - Multiple Sclerosis and Parkinson's Tissue Bank, the Oxford Brain Bank, and the Netherlands Brain Bank). Subjects composing the control cohort did not present any signs of neurodegenerative diseases. Clinical features provided include age at death, postmortem interval (until the brains were sampled), disease onset, disease duration, and brain bank.

In brief, in our initial study[12], we identified distinct molecular subclusters within ALS patients that showed varying patterns in gene, protein and miRNA expression. This suggested the presence of different underlying disease mechanisms and underscored the need for personalized therapeutic approaches. Another important aspect of our study was the identification of pronounced sex differences captured in the molecular profiles of ALS patients, with male patients exhibiting more pronounced alterations overall. Furthermore, our study emphasized and focused on the MAPK pathway as a critical therapeutic target. The involvement of this pathway suggests it could be a focal point for developing targeted treatments, which could improve the prognosis for ALS patients. Other important pathways identified in our initial study were the activation of immune response, extracellular matrix composition, mitochondrial function, and RNA processing. The results from human analyses were corroborated in the selected ALS mouse models, which exhibited similar molecular patterns, and partially resembled the human subclusters revealed through the analysis of human brain tissue. The findings summarized here were

validated across multiple datasets, reinforcing the significance of the identified molecular sub clusters and pathways.

In summary, we describe here a findable, accessible, interoperable, and reproducible (FAIR) multiomics analysis workflow including integration steps and accompanying data that are freely available. For this, we used standardized and versioned docker containers provided by Nextflow[17] for the pre-processing steps and documented the data-specific statistical and machine-learning analyses. An overview of this workflow can be found in Fig. 1.

Methods

Experimental Data Acquisition and Preparation

Sample Data Description

This study investigates the molecular mechanisms underlying ALS using samples from human PFC and from four transgenic mouse models. The human cohort includes 51 patients with sALS and 50 control (CTR) subjects (Table 1). The ALS animal models include four genetically modified mouse strains: B6;129S6-Gt(ROSA)26Sortm1(TARDBP*M337V/Ypet)Tlbt/J mice (here simply referred to as TDP-43 mice)[18], B6SJL-Tg(SOD1*G93A)1Gur/J mice[19] (here referred to as SOD1 mice), (Poly)GA-NES/C9orf72(R26(CAG-Isl-175GA)-29×Nes-Cre) mice (here referred to as C9orf72 mice)[20], and Tg (Prnp-FUS)WT3Cshw/J mice (hereafter referred to as FUS mice)[21]. Each animal model cohort consists of ten transgenic and ten non-transgenic mice, balanced for sex (Table 2).

Sample Acquisition Methods

Data collection and handling is reported in Caldi Gomes, Hänzelmann et al. (2024)[12]. All experimental data presented here comply with the relevant ethical regulations. Consent for the donation of brain material for the subjects that compose the human cohorts was handled individually by the brain banks involved in this study. Ethical approval was obtained from the Ethics Committees of the University Medical Center Göttingen (2/8/18 AN) and the Technical University Munich (145/19 S-SR). All animal experiments complied with international and local animal welfare laws and were approved by the

respective regulatory organs for each involved research center. Experiments with transgenic SOD1 and FUS mice were prospectively approved by the Mario Negri Institutional Animal Care and Use Committee and the Italian Ministry of Health (Prot. No. 9F5F5.143/Prot. No. 9F5F5.250). Experiments with C9orf72 transgenic mice followed the regulations from the German Animal Welfare Act (Tierschutzgesetz/Tierschutz-Versuchstierverordnung, Regierungsbezirke Oberbayern, Prot. No. TV 55.2-2532.Vet_02-17-106). Experiments with TDP43 transgenic animals were approved by the (CCD) Centrale Commissie Dierproeven of Utrecht University (CCD license: AVD 1150020171565), in accordance with Dutch animal welfare laws (Wet op de Dierproeven 2014) and European regulations (guideline 2010/63/EU).

Human Postmortem PFC Samples

Human PFC samples were sourced from four brain banks: The Netherlands Brain Bank, London Neurodegenerative Diseases Brain Bank, Imperial College London Multiple Sclerosis and Parkinson's Tissue Bank, and the Oxford Brain Bank. Samples were shipped on dry ice and stored at -80 °C upon arrival at the rechts der Isar Hospital Department of Neurology, Technical University of Munich. PFC samples were sectioned using a cryostat at -20 °C and processed to collect approximately 20 mg of tissue per sample, which was stored at -80 °C until further use.

ALS Animal Models

Four transgenic mouse models were used to represent the most frequent ALS-causing genes. The mice were kept in pathogen-free facilities with a 12-hour light/dark cycle and unrestricted access to food and water. Each mouse model was euthanized at specific presymptomatic or early symptomatic stages for biomaterial collection. Euthanization times were: TDP-43 (26 weeks), SOD1 (14 weeks), C9orf72 (4.5 weeks), and FUS (four weeks). Mice were perfused with ice-cold phosphate-buffered saline (PBS) before microdissection. The prefrontal cortex was isolated, transferred to nuclease-free tubes, and stored at -80 °C until RNA and protein isolation. For each model, a total of 20 transgenic and control (wild type) mice were selected and balanced for condition/sex (TDP-43: 5 females and 5 males for transgenic and control cohorts; SOD1: 5 females

and 6 males for the control cohort; 5 females and 4 males for the transgenic cohort; C9orf72: 6 females and 4 males for the control cohort; 4 females and 6 males for the transgenic cohort; FUS: 5 females and 5 males for transgenic and control cohorts).

RNA Isolation

Total RNA from human and animal PFC samples was isolated using TRIzol Reagent. RNA was precipitated, washed with ethanol, reconstituted in nuclease-free water, and treated with DNase to remove DNA contamination. Nucleic acid concentration and purity were assessed using a NanoDrop One spectrophotometer and an Agilent 6000 NanoKit for RNA integrity.

RNA Sequencing

mRNA and small RNA sequencing (RNAseq) experiments were conducted as single end at the Functional Genomics Center Zürich. For mRNA sequencing, the TruSeq Stranded mRNA Kit and the SMARTer Stranded Total RNA-Seq Kit v2 Pico Input Mammalian were used. The RealSeq-AC miRNA was used for small RNAseq experiments. After library preparation, normalization was done using Tris–Cl (pH 8.5) containing 0.1% Tween 20 (at 10 nM for the TruSeeq kit, 5 nM for the SMARTer Stranded kit, and 2nM for the RealSeq-AC miRNA kit). Sequencing was performed in the Illumina NovaSeq 6000 platform (for RNAseq) and the HiSeq 2500 platform (for small RNAseq).

Proteomics

Proteins from human and mouse PFC tissue samples were extracted with a biosmasher using 350 µL of MeOH:H_2O (4:1), resuspended in 200 µL Laemmli buffer (10% SDS, Tris 1M pH 6.8, glycerol) then centrifuged at 11.135 rpm at 4°C for 5 minutes. 100 µg of protein lysate was denatured by heating at 95°C for 5 minutes and stacked in an in-house prepared 5% acrylamide SDS-PAGE stacking gel. Gel bands were reduced and alkylated. Digestion was performed overnight at 37°C using modified porcine trypsin (Mass Spec Grade, Promega, Madison, USA; enzyme:protein ratio of 1:80). The peptides were extracted by sequential application of 60% acetonitrile and 100% acetonitrile (ACN).

Peptides were resuspended in 30 µL of $H_2$0, 2% ACN, 0.1% FA and iRT peptides (Biognosys, Schlieren, Switzerland) were added according to the manufacturer's instructions. The generated samples were analyzed using nanoLC–MS/MS (nanoAcquity UltraPerformance LC, Waters, Milford, MA, USA), coupled to a Q-Exactive Plus Mass Spectrometer (Thermo Fisher Scientific). Data was further processed using MaxQuant[22].

In addition, an open modification search was performed. MGF files from the mouse and human proteomics data were loaded into IonBot[23] software (v. 0.11.0). Provided databases were used, either human (9606 entries) or mus musculus (10090 entries), with a K|R cleavage pattern. Error tolerances were set on default values: MS precursor tolerance at 20 ppm and MS/MS fragment tolerance at 0.02 Da. Methionine oxidation and protein N-term acetylation were set as variable modifications, while cysteine carbamidomethylation as a fixed modification. Open modification search option was enabled.

Data Preparation

mRNAseq and small RNAseq Data Processing

RNAseq data were processed using the Nextflow[17] Core RNASeq pipeline version 3.0 with the following parameters *--igenomes_ignore true --fasta <version>.genome.fa.gz --gtf gencode.<version>.annotation.gtf.gz --pseudo_aligner salmon --gencode --deseq2_vst.* Quality checks were conducted with FastQC[24] (Fig. 2) and preprocessing steps included adapter trimming and quality filtering to remove low-quality reads and artifacts. Salmon[25] was used for pseudo-alignment and quantitation, with indices built from GENCODE[26] annotations GRCm39 for mouse and GRCh38 for human. Small RNAseq data were processed using the Nextflow[17] Core smRNASeq pipeline version 1.0 with the parameters *--genome <genome> --mirna_gtf mirbase_<species>.gff3*. FastQC[24] (Fig. 3) and miRTrace[27] were used for quality checks, followed by adapter trimming and quality filtering. Alignment was performed with Bowtie[28], and feature counting utilized samtools[29] using miRBase[30] annotations (version 22.1).

Filtering and Transformation

For RNAseq and small RNAseq count matrices were filtered to retain features with at least ten counts in 50% of samples for any condition or sex. For the small RNAseq data normalization was performed using quantile normalization implemented in the preprocessCore[31] R package. For RNAseq, variance-stabilizing transformation (VST) implemented in DESeq2[32] was used for normalization, ensuring consistent and comparable expression values across samples. The total number of detected genes and sRNAs for each dataset can be found in Table 3.

Proteomics

Proteomics data were processed with MaxQuant[22] software. Protein peaks were assigned using trypsin/P specificity against an in-house generated protein sequence database containing mouse entries from UniProtKB-SwissProt. The "match between runs" option facilitated protein quantification. Only Swiss-Prot proteins were retained and low-abundance proteins detected in less than 50% of samples were filtered out. Missing values were imputed using the missForest[33] algorithm and intensities were log2-transformed for variance stabilization. A maximum false discovery rate (FDR) of 1% was applied at both peptide and protein levels. The total number of detected proteins for each dataset can be found in Table 3.

Higher-Level Data Analysis and Machine Learning

Differential Expression and Enrichment Analyses

Downstream analyses of the RNAseq and small RNAseq data included differential expression analysis using DESeq2[32] to identify differentially expressed genes/miRNAs between experimental conditions. Principal Component Analysis (PCA) was used for dimensionality reduction and

visualization of sample relationships using VST-normalized RNAseq data and quantile-normalized small RNAseq data.

Proteomics Data Analysis

Linear modeling for differential abundance analysis was performed using the limma[34] package, with p-values adjusted for multiple testing using the Benjamini–Hochberg correction. PCA was used for visualization.

Bioinformatics workflow

To allow for reproducible and interpretable bioinformatics workflow, we'll describe the construction of our workflow here. Our workflow consists of multiple stages, equivalent to single scripts executed for one or multiple datasets with multiple parameters. We used Data Version Control (DVC) as a workflow management tool, because it allows the use of any script as stages in our computational workflow, automatically takes care of dependencies between these stages, and executes only stages that changed compared to the last execution. Furthermore, it provides the option to share raw and processed data between multiple users. The executed code for each stage is provided as script files written in bash, R, and Python. The execution of the scripts, their outputs, and their dependencies are defined in dvc.yaml files, with all important parameters found in params.yaml files. All scripts can be automatically executed using DVC. Since the execution of scripts depends on the package versions used, in R as well as in Python, proper maintenance of package versions is important.

Therefore we containerized all applications by providing a docker image. In other instances, we used readily available docker images. This allows the automatic execution of our workflow using docker, if available, and prevents users from struggling to install the correct package versions. Furthermore, we value community efforts in providing reproducible workflows for the analysis of RNAseq & small RNAseq data implemented in Nextflow. These pipelines were

integrated into our workflow as well, allowing us to easily adapt to recent developments in the workflow without much user effort.
All outputs of our workflow will be structured by mouse model and can be used for other applications, integrated into the workflow or not. However, we recommend integrating further analysis into the DVC workflow, as not to break the reproducibility principle of the extended workflow. In this case, also newly added scripts will automatically be executed if the underlying data or scripts are changing in any way.

Data Validation and quality control

RNAseq Data

In addition to the quality checks mentioned above, the quality of the dataset was evaluated. To verify the annotation of sex, the expression of *XIST* was investigated in each sample (Fig. 4). *XIST* is a long non-coding RNA, involved in X-chromosome-inactivation, and therefore highly expressed in females[35]. We could not detect any mismatched sex annotation in the human or mouse samples (Fig. 4).
Furthermore, we validated the expression of the transgenic variant for the FUS, SOD1, and TDP43 mouse models. The transgenic mouse models were generated by including the mutated human gene (SOD1 and TDP43) or overexpressing the wild-type human gene (FUS) in the mouse genome. Therefore, the fraction of reads aligning against this region of interest was compared to the total number of reads in that region (Fig. 5). The Region of interest was defined as ±200 bp around the gene's coding region. It is expected that control samples don't show any expression of human reads, while the mutated samples show a significant expression of the human variant. Thus, we could verify the expression of the transgene in these three mouse models (Fig. 5A).
The C9orf72 mouse model was generated by introducing a repeat expansion in the intronic region of C9orf72, which cannot be detected using the approach used for the other mouse models. Therefore, we used an indirect approach to detect the GFP expression of the construct used for integrating the repeat expansion[20]. We were able to detect the expression of the construct only in

transgenic animals, thus indicating that the introduced repeat expansion is likely present as well in these animals (Fig 5B). Furthermore, the expression data was visualized using a histogram for each sample, showing no distinct pattern for individual samples (Fig. 6). Therefore, we consider the RNAseq data good quality matching with the provided annotations.

small RNAseq Data

The quality of the small RNAseq data was additionally evaluated using miRTrace[27] as part of the Nextflow smrnaseq pipeline. miRTrace detected 17.07% reads as originating from miRNAs on average across the models (Human: 10.12%, SOD1 29.05%, FUS: 13.37%, TDP43: 32.29%, C9orf72: 28.01%) and only a low percentage of artifacts (mean < 6%). We could also not observe any large difference in the detected RNA types across samples (Fig. 7). However, the human samples showed a lower number of reads assigned to any class compared to the mouse samples.

Similar to the RNAseq data also a histogram of the miRNA expression was visualized (Fig. 8). The mouse models show a consistent expression pattern across samples, with only minor differences between the mouse models, conditions, and sexes. For the human samples, we observed a consistent expression pattern for most samples (Fig. 8).

Proteomics Data

The quality of the proteomics data was evaluated by calculating the fraction of measured zero values and the histogram of protein abundance values. We could not observe any significant difference between the fraction of zero-measurements in the proteomics data (Fig. 9), indicating that there is no systematic bias impacting the sample quality. Furthermore, no systematic difference between the samples could be detected in the histograms of the normalized protein abundances (Fig. 10). Differential protein abundance analysis was conducted and a calibration analysis was performed to verify that the obtained p-values followed the assumptions of classical false discovery rate (FDR) control[36]. We detected a high differential protein abundance

concentration (differential concentration > 83%) and a low uniformity underestimation (< 0.02) in all models (Fig. 11). This indicates that there are likely no violations of the FDR control assumptions. In addition, we performed an open modification search, but did not detect any striking differences between the mouse models and human samples (Fig. 12). Overall, we detected no systematic biases, low inter-sample intra-model variability (especially for the mouse samples), and proven expression of the transgenes. Therefore, in our opinion, the dataset provides a unique resource for the (re-)analysis of ALS considering multiple known ALS mouse models and human samples.

Batch effects

To assess the possibility of batch effects in transcriptomics and proteomics data, principle component analysis, and sample distance heatmaps were used. Specifically, batch effects related to the factors brain bank, sex, case/control condition, as well as age at death were investigated. As reported in the previous publication (Figure 13; Figure 1b of Caldi Gomes, Hänzelmann et al., 2024[12]) sex-related differences were found, and all analyses were performed separately for each sex. For the other factors, there is no evidence that they influenced the results (Figure 13; Supplementary Figure 1 of Caldi Gomes, Hänzelmann et al., 2024). For instance, if batch effects had been present, they would likely have caused distinct clustering or separation of samples. However, our analysis showed no such patterns, indicating that these factors did not introduce systematic biases into our data.

Results Summary

In brief, in our initial study[12], this dataset revealed distinct molecular subclusters within ALS patients. These subclusters showed varying patterns in gene, protein, and miRNA expression, suggesting the presence of different underlying disease mechanisms. One of these identified mechanisms was the MAPK pathway as a putative therapeutic target. Another important aspect of our study was the pronounced sex differences captured in the molecular profiles of ALS patients, with male patients exhibiting more pronounced alterations overall.

The findings summarized here were validated across multiple models, reinforcing the significance of the identified molecular subclusters and pathways. This validation suggests that future ALS research should consider these frequently reported molecular differences, and focus on developing personalized medicine approaches, tailored to specific patient subgroups.

Re-use potential

Our complex cross-species and sex-specific data can serve as a basis for future computational and experimental studies. Further, the stratification of ALS patients into specific subtypes through our multi-omic data could help with developing personalized, sex-specific, and efficient treatment approaches. Furthermore, newly found treatment candidates can be directly investigated in the four available mouse models, to detect the potentially best mouse model for in vivo testing. Furthermore, this rich resource of human sALS and mouse models for gALS could be utilized to detect subtle differences between sALS and gALS, e.g. on splicing level, which are currently not well understood and can provide new biomarkers or treatment options in the early stages of ALS. To facilitate future usage, intermediate files are saved in a format that is readable using most common programming languages, mainly in CSV format, allowing for flexible integration of new methods at every step of the existing pipeline. Several downstream applications, such as differential gene expression analysis, are already implemented and can be executed using DVC. Furthermore, these methods are highly configurable using the parameter files and allow for a multitude of different analyses. To achieve continuous high reproducibility, we recommend the implementation of executable scripts, which can be automatically executed by DVC.

In addition, the repository provides code for the analysis of transcription factor activity, RNA stability, and possible RNA variants. Further details can be found in the three following sections.

Transcription factor activity

Transcription factor activity was estimated using decoupleR[37] with default settings. For activity estimation, a univariate linear model, a weighted sum, and a multivariate linear model were used as recommended by decoupleR. The DoRothEA[38] database was used for potential transcription factor targets. Only targets of at least category C were used (Category A = high confidence, category E = low confidence).

RNA stability analysis

RNA stability analysis was performed using REMBRANDTS[39]. Exon and intron regions, as required for the analysis with REMBRANDTS were extracted from GENCODE v37 annotations for the human data and GENCODE vM26 for the mouse data. The quantification of exon and intron abundance was performed using htseq (v1.99.2) as described in Alkallas et al[39] and the REMBRANDTS manual. REMBRANDTS was run using default arguments with a linear bias-mode, a stringency of 0.99, and no further batch information.

Variant analysis

To analyze variations in the mRNA, we performed variant calling on the RNAseq data. Bcftools (v1.14) was used for variant calling based on the STAR alignments as provided by the NextFlow Core RNASeq pipeline, version 3.0 described in Ewels et al.[17].

Conclusion

In summary, our study provides a valuable data resource, including sex-specific and cross-species datasets. The stratified multi-omics data from ALS prefrontal cortices highlights male and female differences, with implications for future personalized treatment approaches. Additionally, we offer a robust analysis pipeline and high-quality data for investigating early ALS mechanisms.

Our methods ensure robust comparability and reproducibility of the analysis across all generated datasets, both within omics layers for the analyzed cohorts, and also across species. Overall, these datasets can help with the understanding of ALS pathogenesis and assist in identifying new and

personalized therapeutic targets for this devastating neurodegenerative disease.

Data Availability

The workflow contains scripts for the automatic download of all mouse sequencing data from SRA to be used as input to the workflow. However, the workflow can also be used with manually downloaded files, which is required for human samples, due to the restricted access. Details about how to access the human data deposited in European Genome Phenome Archive are available online[40]. All supporting data and materials are available in the *GigaScience* GigaDB database[41].

Mouse RNAseq data

Raw RNAseq data (FASTQ format) and processed data (CSV format) were deposited to the National Center for Biotechnology Information Gene Expression Omnibus database (GSE234245) and are openly available.

Mouse small RNAseq data

Raw small RNAseq data (FASTQ format) and processed data (CSV format) were deposited to the National Center for Biotechnology Information Gene Expression Omnibus database (GSE234243) and are openly available.

Human RNAseq & small RNAseq data

Human raw data (FASTQ format) is encrypted and stored at the European Genome Phenome Archive (registered study: EGAS00001007318). This data is available upon request to the European Genome Phenome Archive. Details about how to access the human data deposited in European Genome Phenome Archive are available online[40].

Proteomics data

Human and mouse proteomics data were deposited to the ProteomeXchange Consortium database (PXD043300) and are openly available. The results from the open modification search are available via figshare[42].

Availability of source code and requirements

Project name: MAXOMOD

Project home page: https://github.com/imsb-uke/MAXOMOD_Pipeline

Operating system(s): Platform independent

Programming language: Python, R, Bash, Nextflow

Other requirements: DVC (https://dvc.org/), Docker

License: MIT

Workflowhub.eu: https://doi.org/10.48546/workflowhub.workflow.1191.1

All code for preprocessing and analyzing the data is available online[43]. The workflow consists of multiple scripts R & Python (`src/directory`) which can be executed in a dockerized environment which is provided on GitHub as well. The execution order is provided as a Data Version Control workflow, which can be automatically executed with DVC. All parameters are provided in the params/ directory. Further information can be found in the `README` file in the GitHub repository.

List of abbreviations

PFC: prefrontal cortex

ALS: Amyotrophic lateral sclerosis

sALS: sporadic Amyotrophic lateral sclerosis

gALS: genetic Amyotrophic lateral sclerosis

CTR: control

PBS: phosphate-buffered saline

RNAseq: RNA sequencing

FDR: false discovery rate

PCA: Principal Component Analysis

DVC: Data Version Control

Acknowledgments

We thank all the members of the Lingor, Bonn, Carapito, Schlapbach, Pasterkamp, Edbauer, and Bonetto laboratories for their contribution to the generation of the omics data and subsequent analyses. This work was performed by the research consortium “Multi-omic analysis of axono-synaptic degeneration in motoneuron disease (MAXOMOD)” funded in the scope of the E-Rare Joint Transnational Call for Proposals 2018 “Transnational research projects on hypothesis-driven use of multi-omic integrated approaches for discovery of disease causes and/or functional validation in the context of rare diseases.” granted to the labs of SB, VB, CC, RS, and PL. LCG, MP, LT, and PL were supported by the Bundesministerium für Bildung und Forschung (01GM1917A), and by the Munich Cluster for Systems Neurology (SyNergy). SH received funding from BMBF (grant 01GM2202A; STOP- FSGS and DFG, CRC1192). SO was funded by CRC1286 SP02 and KFO296 P8. RK was funded by FOR5068 P9 and FH by the M3I excellence initiative and a UKE postdoctoral stipend. SB was funded by CRC1286 SP02, CRC1192 PB8, and PC3. We thank Tobias B. Huber for his support. The funders had no role in designing the study or generating the data/analyses in this work.

Author contributions

PL and LCG conceived the project, designed the sample collection methodology, reviewed the sample/data quality, and coordinated the acquisition of human tissue samples from the selected brain banks. SH, FH, RK, SO, and SB conceptualized the data analysis. LCG, MP, LT, LP, MG, PZ, and QZ processed postmortem brain material for multiomics experiments with the infrastructure provided by RJP, DE, VB, CC, and PL. MG and HR performed high-throughput sequencing experiments (RNAseq/mass spectrometry runs, respectively) with the infrastructure provided by CC and RS. SH, FH, RK, and SO were responsible for the bioinformatics analysis, and conceived/performed the statistical analyses. FH, SH, and LCG wrote the manuscript; PL and SB

reviewed the manuscript with input from all co-authors. LCG, SH, and FH contributed equally. SB and PL contributed equally.

Competing interests

The authors declare no competing interests.

References

1. Goutman, S. A. Diagnosis and Clinical Management of Amyotrophic Lateral Sclerosis and Other Motor Neuron Disorders. *Contin. Minneap. Minn* **23**, 1332–1359 (2017). https://doi.org/10.1212/con.0000000000000535
2. Suzuki, N., Nishiyama, A., Warita, H. & Aoki, M. Genetics of amyotrophic lateral sclerosis: seeking therapeutic targets in the era of gene therapy. *J. Hum. Genet.* **68**, 131–152 (2023). https://doi.org/10.1038/s10038-022-01055-8
3. Chia, R., Chiò, A. & Traynor, B. J. Novel genes associated with amyotrophic lateral sclerosis: diagnostic and clinical implications. *Lancet Neurol.* **17**, 94–102 (2018). https://doi.org/10.1016/s1474-4422(17)30401-5
4. Talbott, E. O., Malek, A. M. & Lacomis, D. The epidemiology of amyotrophic lateral sclerosis. *Handb. Clin. Neurol.* **138**, 225–238 (2016). https://doi.org/10.1016/b978-0-12-802973-2.00013-6
5. Kim, G., Gautier, O., Tassoni-Tsuchida, E., Ma, X. R. & Gitler, A. D. ALS Genetics: Gains, Losses, and Implications for Future Therapies. *Neuron* **108**, 822–842 (2020). https://doi.org/10.1016/j.neuron.2020.08.022
6. Holm, A., Hansen, S. N., Klitgaard, H. & Kauppinen, S. Clinical advances of RNA therapeutics for treatment of neurological and neuromuscular diseases. *RNA Biol.* **19**, 594–608 (2022). https://doi.org/10.1080/15476286.2022.2066334
7. Aronica, E. *et al.* Molecular classification of amyotrophic lateral sclerosis by unsupervised clustering of gene expression in motor cortex. *Neurobiol. Dis.* **74**, 359–376 (2015). https://doi.org/10.1016/j.nbd.2014.12.002

8. Morello, G. *et al.* Integrative multi-omic analysis identifies new drivers and pathways in molecularly distinct subtypes of ALS. *Sci. Rep.* **9**, 9968 (2019). https://doi.org/10.1038/s41598-019-46355-w

9. Tam, O. H. *et al.* Postmortem Cortex Samples Identify Distinct Molecular Subtypes of ALS: Retrotransposon Activation, Oxidative Stress, and Activated Glia. *Cell Rep.* **29**, 1164-1177.e5 (2019). https://doi.org/10.1016/j.celrep.2019.09.066

10. Brettschneider, J. *et al.* Stages of pTDP-43 pathology in amyotrophic lateral sclerosis. *Ann. Neurol.* **74**, 20–38 (2013). https://doi.org/10.1002/ana.23937

11. Brettschneider, J. *et al.* TDP-43 pathology and neuronal loss in amyotrophic lateral sclerosis spinal cord. *Acta Neuropathol. (Berl.)* **128**, 423–437 (2014). https://doi.org/10.1007/s00401-014-1299-6

12. Caldi Gomes, L. *et al.* Multiomic ALS signatures highlight subclusters and sex differences suggesting the MAPK pathway as therapeutic target. *Nat. Commun.* **15**, 4893 (2024). https://doi.org/10.1038/s41467-024-49196-y

13. Humphrey, J. *et al.* Integrative transcriptomic analysis of the amyotrophic lateral sclerosis spinal cord implicates glial activation and suggests new risk genes. *Nat. Neurosci.* **26**, 150–162 (2023). https://doi.org/10.1038/s41593-022-01205-3

14. Chen, G.-B. *et al.* Integrated multi-omics analysis identifies novel risk loci for amyotrophic lateral sclerosis in the Chinese population. Research Square; 2024 Preprint at https://doi.org/10.21203/rs.3.rs-3967132/v1 .

15. Grima, N., Henden, L., Watson, O., Blair, I. P. & Williams, K. L. Simultaneous Isolation of High-Quality RNA and DNA From Postmortem Human Central Nervous System Tissues for Omics Studies. *J. Neuropathol. Exp. Neurol.* **81**, 135–145 (2022). https://doi.org/10.1093/jnen/nlab129

16. Morello, G., Salomone, S., D'Agata, V., Conforti, F. L. & Cavallaro, S. From Multi-Omics Approaches to Precision Medicine in Amyotrophic Lateral Sclerosis. *Front. Neurosci.* **14**, 577755 (2020). https://doi.org/10.3389/fnins.2020.577755

17. Ewels, P. A. *et al.* The nf-core framework for community-curated bioinformatics pipelines. *Nat. Biotechnol.* **38**, 276–278 (2020). https://doi.org/10.1038/s41587-020-0439-x

18. Gordon, D. *et al.* Single-copy expression of an amyotrophic lateral sclerosis-linked TDP-43 mutation (M337V) in BAC transgenic mice leads to altered stress granule dynamics and progressive motor dysfunction. *Neurobiol. Dis.* **121**, 148–162 (2019). https://doi.org/10.1016/j.nbd.2018.09.024

19. Gurney, M. E. *et al.* Motor neuron degeneration in mice that express a human Cu,Zn superoxide dismutase mutation. *Science* **264**, 1772–1775 (1994). https://doi.org/10.1126/science.8209258

20. LaClair, K. D. *et al.* Congenic expression of poly-GA but not poly-PR in mice triggers selective neuron loss and interferon responses found in C9orf72 ALS. *Acta Neuropathol. (Berl.)* **140**, 121–142 (2020). https://doi.org/10.1007/s00401-020-02176-0

21. Mitchell, J. C. *et al.* Overexpression of human wild-type FUS causes progressive motor neuron degeneration in an age- and dose-dependent fashion. *Acta Neuropathol. (Berl.)* **125**, 273–288 (2013). https://doi.org/10.1007/s00401-012-1043-z

22. Tyanova, S., Temu, T. & Cox, J. The MaxQuant computational platform for mass spectrometry-based shotgun proteomics. *Nat. Protoc.* **11**, 2301–2319 (2016). https://doi.org/10.1038/nprot.2016.136

23. Degroeve, S. et al. ionbot: a novel, innovative and sensitive machine learning approach to LC-MS/MS peptide identification. Preprint at https://doi.org/10.1101/2021.07.02.450686 (2021).
24. Andrews, S. FastQC: A Quality Control Tool for High Throughput Sequence Data. http://www.bioinformatics.babraham.ac.uk/projects/fastqc/. Accessed 2nd Nov 2024.
25. Patro, R., Duggal, G., Love, M. I., Irizarry, R. A. & Kingsford, C. Salmon provides fast and bias-aware quantification of transcript expression. *Nat. Methods* **14**, 417–419 (2017). https://doi.org/10.1038/nmeth.4197
26. Frankish, A. *et al.* GENCODE 2021. *Nucleic Acids Res.* **49**, D916–D923 (2021). https://doi.org/10.1093/nar/gkaa1087
27. Kang, W. *et al.* miRTrace reveals the organismal origins of microRNA sequencing data. *Genome Biol.* **19**, 213 (2018). https://doi.org/10.1186/s13059-018-1588-9
28. Langmead, B., Trapnell, C., Pop, M. & Salzberg, S. L. Ultrafast and memory-efficient alignment of short DNA sequences to the human genome. *Genome Biol.* **10**, R25 (2009). https://doi.org/10.1186/gb-2009-10-3-r25
29. Danecek, P. *et al.* Twelve years of SAMtools and BCFtools. *GigaScience* **10**, giab008 (2021). https://doi.org/10.1093/gigascience/giab008
30. Griffiths-Jones, S., Grocock, R. J., van Dongen, S., Bateman, A. & Enright, A. J. miRBase: microRNA sequences, targets and gene nomenclature. *Nucleic Acids Res.* **34**, D140-144 (2006). https://doi.org/10.1093/nar/gkj112
31. Bolstad, B. bmbolstad/preprocessCore. Github. https://github.com/bmbolstad/preprocessCore. Accessed 2nd Nov 2024.

32. Love, M. I., Huber, W. & Anders, S. Moderated estimation of fold change and dispersion for RNA-seq data with DESeq2. *Genome Biol.* **15**, 550 (2014). https://doi.org/10.1186/s13059-014-0550-8
33. Stekhoven, D. J. & Bühlmann, P. MissForest--non-parametric missing value imputation for mixed-type data. *Bioinforma. Oxf. Engl.* **28**, 112–118 (2012). https://doi.org/10.1093/bioinformatics/btr597
34. Ritchie, M. E. *et al.* limma powers differential expression analyses for RNA-sequencing and microarray studies. *Nucleic Acids Res.* **43**, e47 (2015). https://doi.org/10.1093/nar/gkv007
35. Chow, J. C., Yen, Z., Ziesche, S. M. & Brown, C. J. Silencing of the mammalian X chromosome. *Annu. Rev. Genomics Hum. Genet.* **6**, 69–92 (2005). https://doi.org/10.1146/annurev.genom.6.080604.162350
36. Giai Gianetto, Q. *et al.* Calibration plot for proteomics: A graphical tool to visually check the assumptions underlying FDR control in quantitative experiments. *Proteomics* **16**, 29–32 (2016). https://doi.org/10.1002/pmic.201500189
37. Badia-I-Mompel, P. et al. decoupleR: ensemble of computational methods to infer biological activities from omics data. Bioinforma. Adv. **2**, vbac016 (2022). https://doi.org/10.1093/bioadv/vbac016
38. Garcia-Alonso, L., Holland, C. H., Ibrahim, M. M., Turei, D. & Saez-Rodriguez, J. Benchmark and integration of resources for the estimation of human transcription factor activities. Genome Res. **29**, 1363–1375 (2019). https://doi.org/10.1101/gr.240663.118
39. Alkallas, R., Fish, L., Goodarzi, H. & Najafabadi, H. S. Inference of RNA decay rate from transcriptional profiling highlights the regulatory programs of Alzheimer's

disease. *Nat. Commun.* **8,** 909 (2017). https://doi.org/10.1038/s41467-017-00867-z

40. EGA database - how to request data. https://ega-archive.org/access/request-data/how-to-request-data/. Accessed 2nd Nov 2024.

41. Hausmann F; Gomes LC; Hänzelmann S; Khatri R; Oller S; Gebelin M; Parvaz M; Tzeplaeff L; Pasetto L; Zhou Q; Zelina P; Edbauer D; Pasterkamp RJ; Rehrauer H; Schlapbach R; Carapito C; Bonetto V; Bonn S; Lingor P. Supporting data for "A Data Set Profiling the Multi-omic Landscape of the Prefrontal Cortex in Amyotrophic Lateral Sclerosis" GigaScience Database 2024. https://doi.org/10.5524/102597

42. MAXOMOD, Figshare files (https://doi.org/10.6084/m9.figshare.27108562). https://figshare.com/articles/dataset/MAXOMOD_OpenModification_Search_Results/27108562?file=49418206. Accessed 2nd Nov 2024.

43. MAXOMOD - Github pipeline. https://github.com/imsb-uke/MAXOMOD_Pipeline. Accessed 2nd Nov 2024.

44. Altschul, S. F., Gish, W., Miller, W., Myers, E. W. & Lipman, D. J. Basic local alignment search tool. *J. Mol. Biol.* **215**, 403–410 (1990). https://doi.org/10.1016/s0022-2836(05)80360-2

45. Workflow: Multiomic ALS signatures highlight sex differences and molecular subclusters and identify the MAPK pathway as therapeutic target (WorkflowHub). https://doi.org/10.48546/workflowhub.workflow.1191.1. Accessed 11th Nov 2024.

Tables

Table 1: Summary of the cohort numbers and demographics for the human cohort

Human cohort	**Control**	**ALS**
**Subjects**	50	51
**Age at death (in years)**	75 (43–94)	67 (44–83)
**Sex (F/M)**	28 F / 22 M	16 F / 35 M
**Disease duration (years)**	-	3 (1–28)
Unprecise/unknown	-	39.2%
**Brain bank origin**		
NBB	18.0%	17.6%
Oxford BB	20.0%	27.5%
ICL MS & PD TB	38.0%	0.0%
London NDBB	24.0%	54.9%

NBB: The Netherlands Brain Bank; OBB: Oxford Brain Bank; ICL MS&PD TB: Imperial College London - Multiple Sclerosis and Parkinson's Tissue Bank; London NDBB: London Neurodegenerative Diseases Brain Bank. ALS, amyotrophic lateral sclerosis. Sex: male = M; female = F. A full description of the clinical features for the human cohort can be accessed within the supplementary data from our main publication.[12]

Table 2: Summary of the cohort numbers for the ALS mouse models

Mouse cohorts	**TDP-43**	
**Condition (WT /TG)**	10 WT	10 TG
**Sex (F/M)**	5 F / 5 M	5 F / 5 M
	**SOD1**	
**Condition (WT /TG)**	11 WT	9 TG
**Sex (F/M)**	5 F / 6 M	5 F / 4 M

	C9orf72	
**Condition (WT /TG)**	10 WT	10 TG
**Sex (F/M)**	6 F / 4 M	6 F / 4 M
	**FUS**	
**Condition (WT /TG)**	10 WT	10 TG
**Sex (F/M)**	5 F / 5 M	5 F / 5 M

Condition: wild type = WT; transgenic = TG. Sex: male = M; female = F.

Table 3: Number of mapped entities for Human and mouse data.

	Transcriptomics	Proteomics	miRNA (mature/hairpin)
**Human**	19641	2363	736 (224/512)
**SOD1 - Mouse**	16583	2854	893 (526/367)
**TDP43 - Mouse**	16801	2802	907 (534/373)
**C9orf72 - Mouse**	17465	2866	754 (271/483)
**FUS - Mouse**	17230	2522	812 (468/344)

Figure Legends

Figure 1: **Overview of the bioinformatics workflow for RNAseq, small RNAseq, and proteomics data.** Methods and processing scripts are shown in orange diamonds, high-throughput technologies depicted in blue rectangles with round edges, and datasets available on disk in green rectangles with round edges, and Nextflow pipelines in red parallelograms. For the Nextflow pipelines, only a few steps are named here. The RNASeq pipeline (v3.0) and the small RNAseq smRNASeq pipeline (v1.0) were used.

Figure 2: Demonstration of overall quality of reads on transcriptome level. Mean phred quality scores, as reported by FastQC of the RNAseq data are displayed. Regions are colored according to FastQC's quality definitions (greed: good, orange: ok, red: bad). Overall all reads show a good quality.

Figure 3: Demonstration of overall quality of reads on small RNA data level. Mean phred quality scores as reported by FastQC are displayed. Regions are colored according to FastQC's quality definitions (greed: good, orange: ok, red: bad). For SOD1 and TDP43 the expected length after trimming is indicated by the gray-dotted line, for the others only the expected length of reads was provided. Overall all reads show a good quality.

Figure 4: **Verification of sex on transcriptome level.** VST-transformed *XIST* expression in human and mouse RNAseq experiments colored by Sex. *XIST* expression confirms the correct sex annotation.

Figure 5: **Verification of the transgenic animals. A**: Fraction of reads from the RNAseq experiments aligning against the human genome in the region of the *Fus*, the *Sod1,* and the *Tardbp* gene (+-200 bp) in the corresponding mouse model. Reads aligning against the human genome, confirm that the corresponding samples express the transgenic transcript correctly. **B:** Number of reads mapping to the pEGFP construct (U76561.1) using blastn[44] (v2.15.0) in C9orf72 transgenic animals.

Figure 6: **Overall quality of transformed transcriptomic data.** Histogram of VST-transformed expression values with samples on the x-axis colored by sex and condition. No strong difference between the sexes and conditions could be observed.

Figure 7: **Evaluation of batch effects (sex and condition).** Barchart of miRTrace quality checks with samples on the x-axis colored by detected RNA type. The fraction of reads that could not be assigned to any of the RNA types is not displayed. No strong difference between the sexes or conditions could be observed.

Figure 8: **Overall quality of transformed small RNAseq data.** Histogram of normalized mature miRNA expression values with samples on the x-axis colored by sex and condition. No strong difference between the sexes and conditions could be observed.

Figure 9: **Completeness of raw proteomics data.** Fraction of measured zeros in the proteomics experiments colored by Sex. No difference in the distribution between the sexes and the condition could be detected.

Figure 10: **Overall quality of transformed proteomics data.** Histogram of normalized protein abundance values with samples on the x-axis colored by sex and condition. No strong difference between the sexes and conditions could be observed.

Figure 11: **Evaluation of proteomics differential protein abundance analysis.** Calibration plots for case vs control differential protein abundance analysis to check if the p-values respect the assumptions of classical FDR control procedures. A high (close to 100%) differential concentration (in green) and a low uniformity underestimation (close to 0) are preferred.

Figure 12: Top modifications found by the open modification search using ionbot for the four mouse models and human samples. For each model, the top 10 modifications were selected and the number of occurrences of the union of those (17 modifications) is displayed. Fixed modifications (Carbamidomethyl, Oxidation, Acetyl[N-term]) and sequence variations (Glu->Ser, Arg->Orn, Ser->Ala, Gln->pyro-Glu, Xle->Pro, Tyr->Phe, Delta:H(2)C(2)[N-term]) were removed for display.

Figure 13: Evaluation of batch effects using principal component analysis. Principal component analysis (PCA) of human transcriptomics (**A**) and proteomics (**B**) data. For the transcriptomics data the 500 most variable genes, and for the proteomics data all proteins were used.

Click here to access/download;Figure;Fig1.pdf

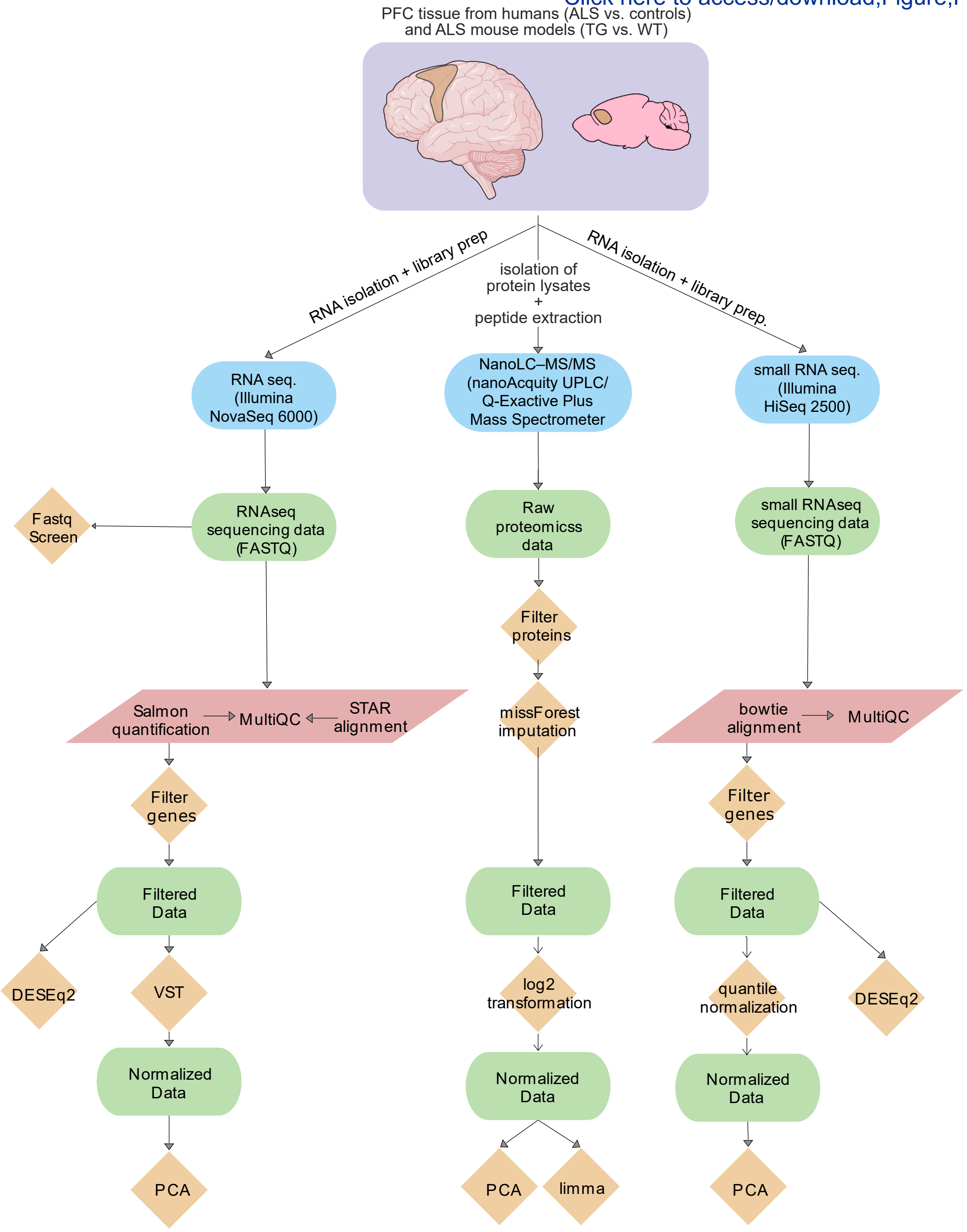

Click here to access/download;Figure;Fig2.pdf

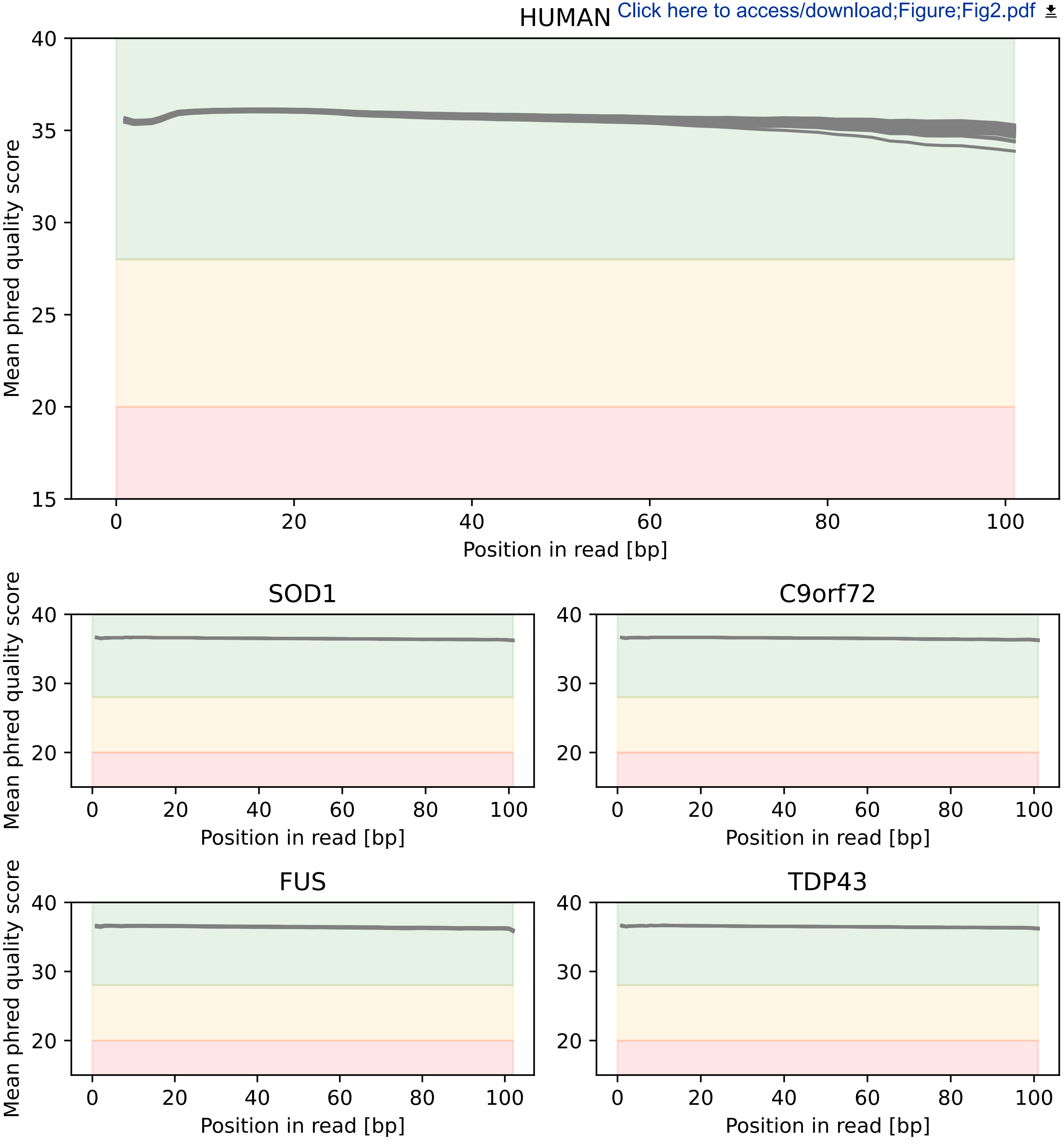

Click here to access/download;Figure;Fig3.pdf

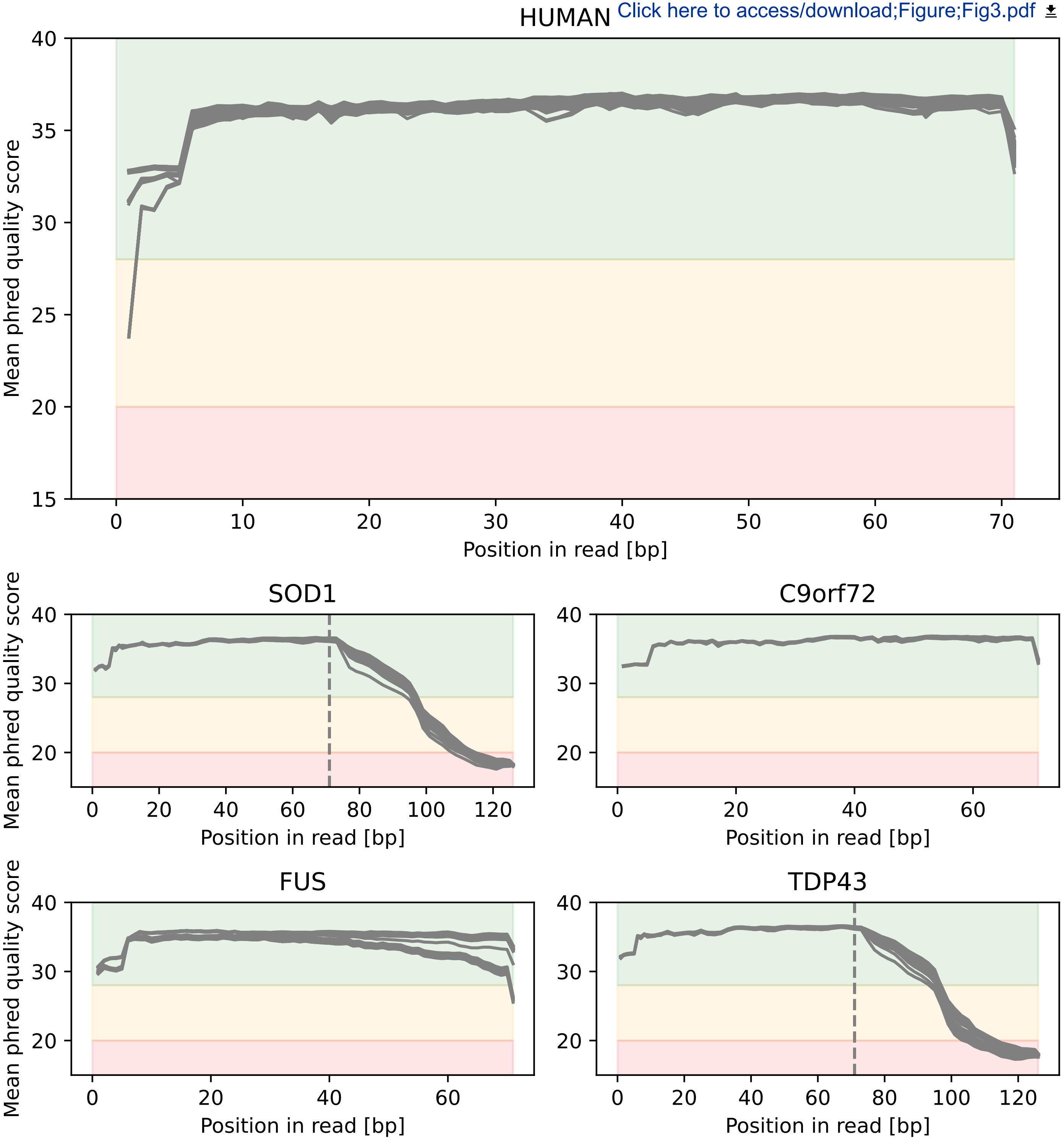

Click here to access/download;Figure;Fig4.pdf

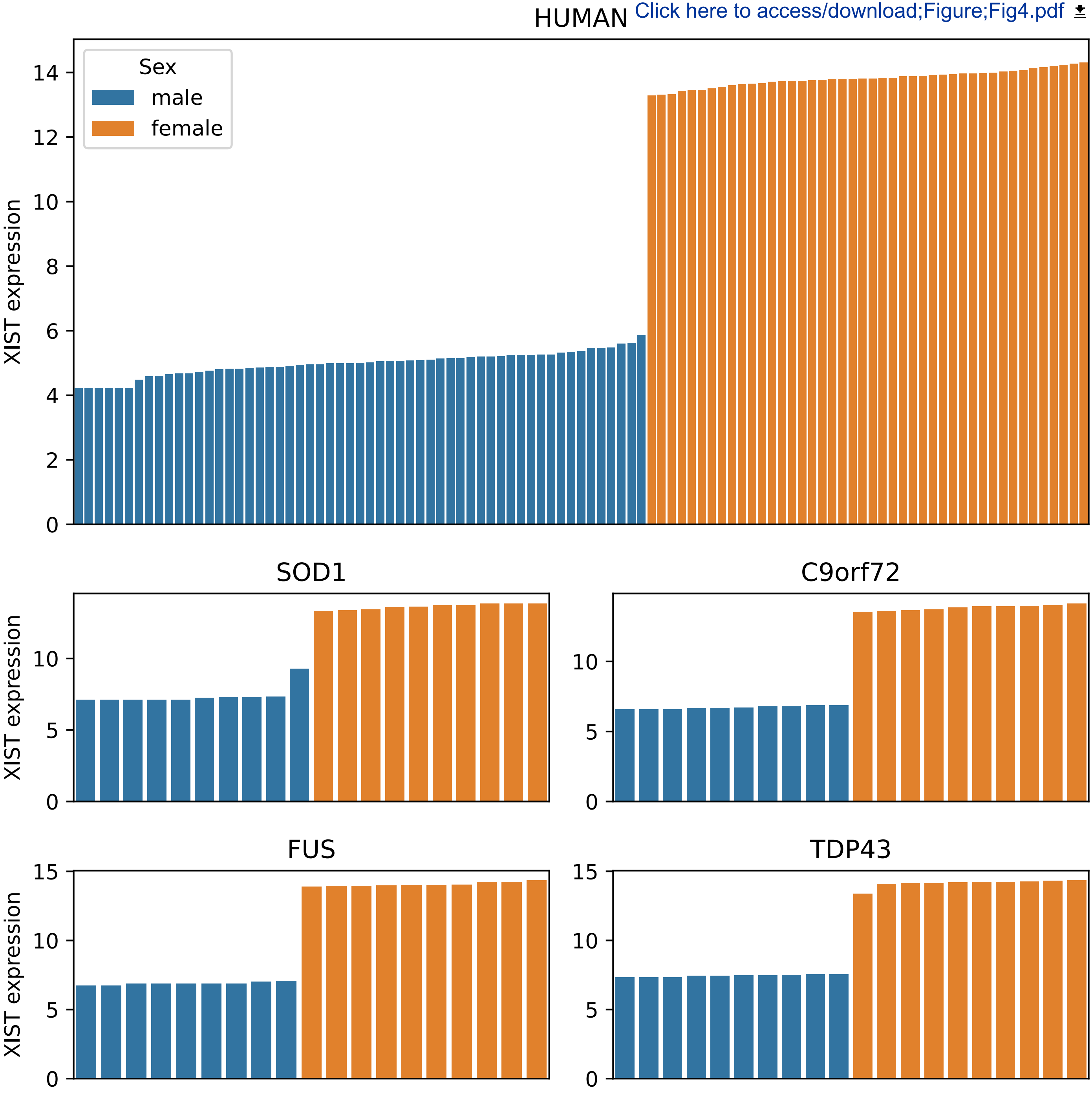

Click here to access/download;Figure;Fig5.pdf

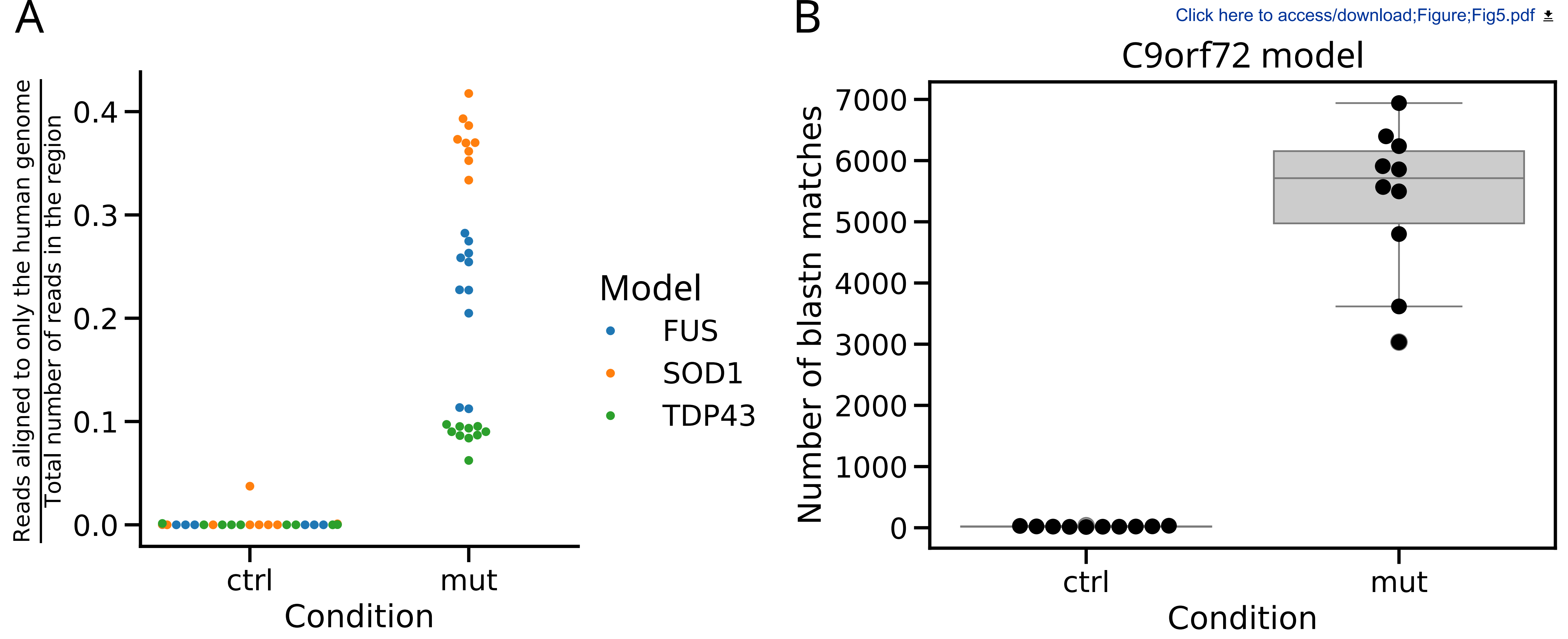

Figure

Click here to access/download;Figure;Figure6.pdf

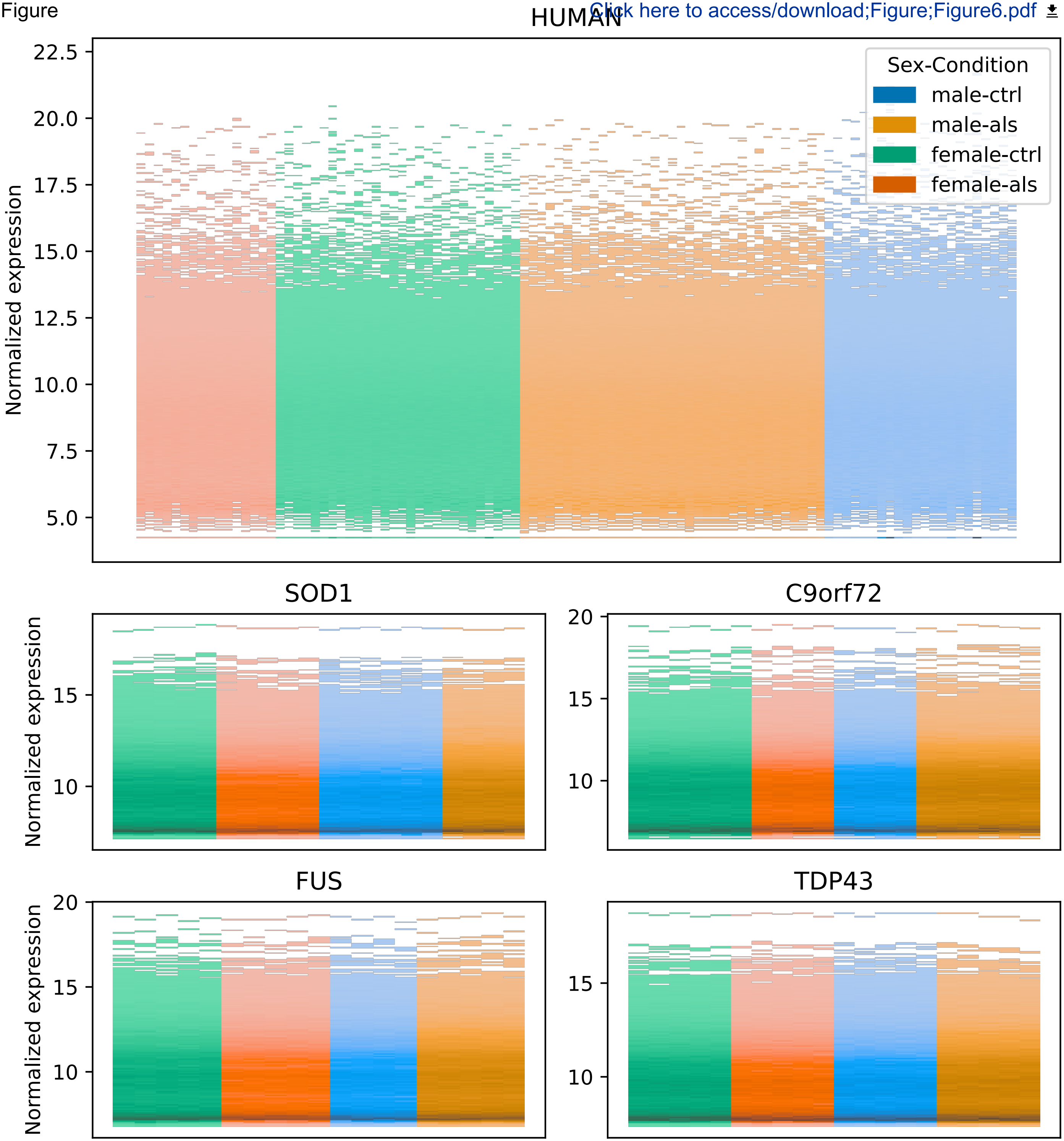

Click here to access/download;Figure;Fig7.pdf

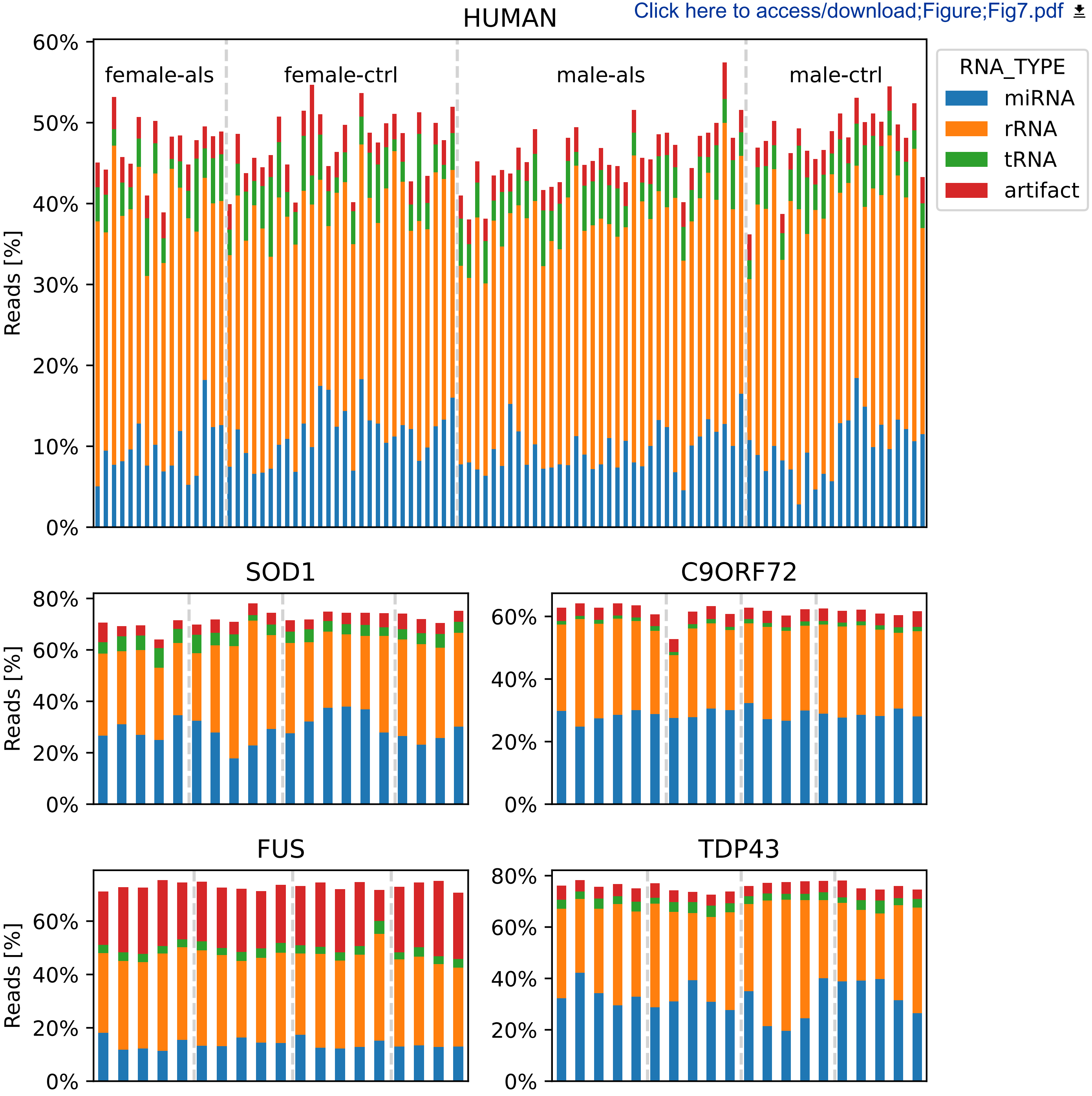

Figure

Click here to access/download;Figure;Figure8.pdf

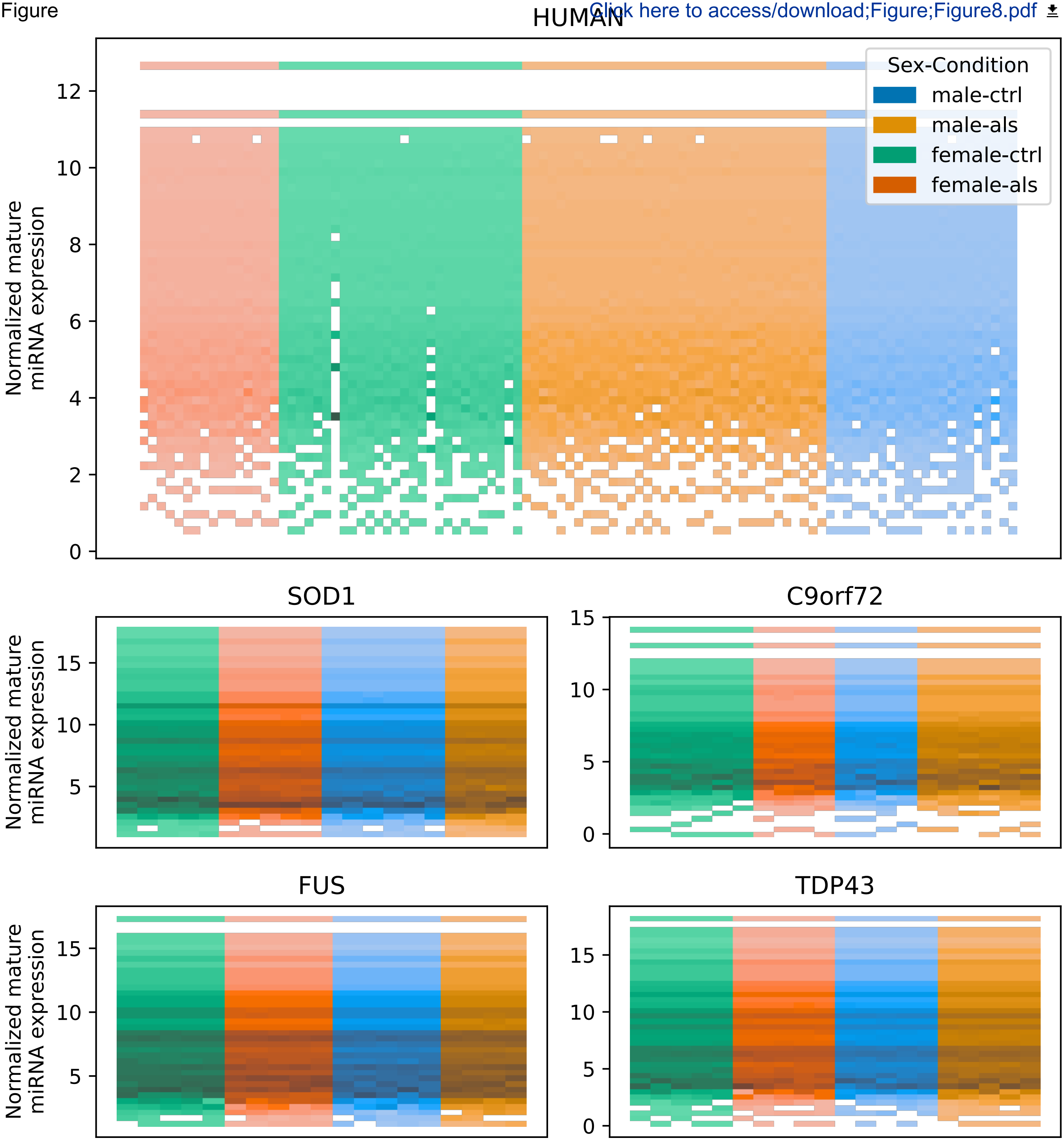

Click here to access/download;Figure;Fig9.pdf

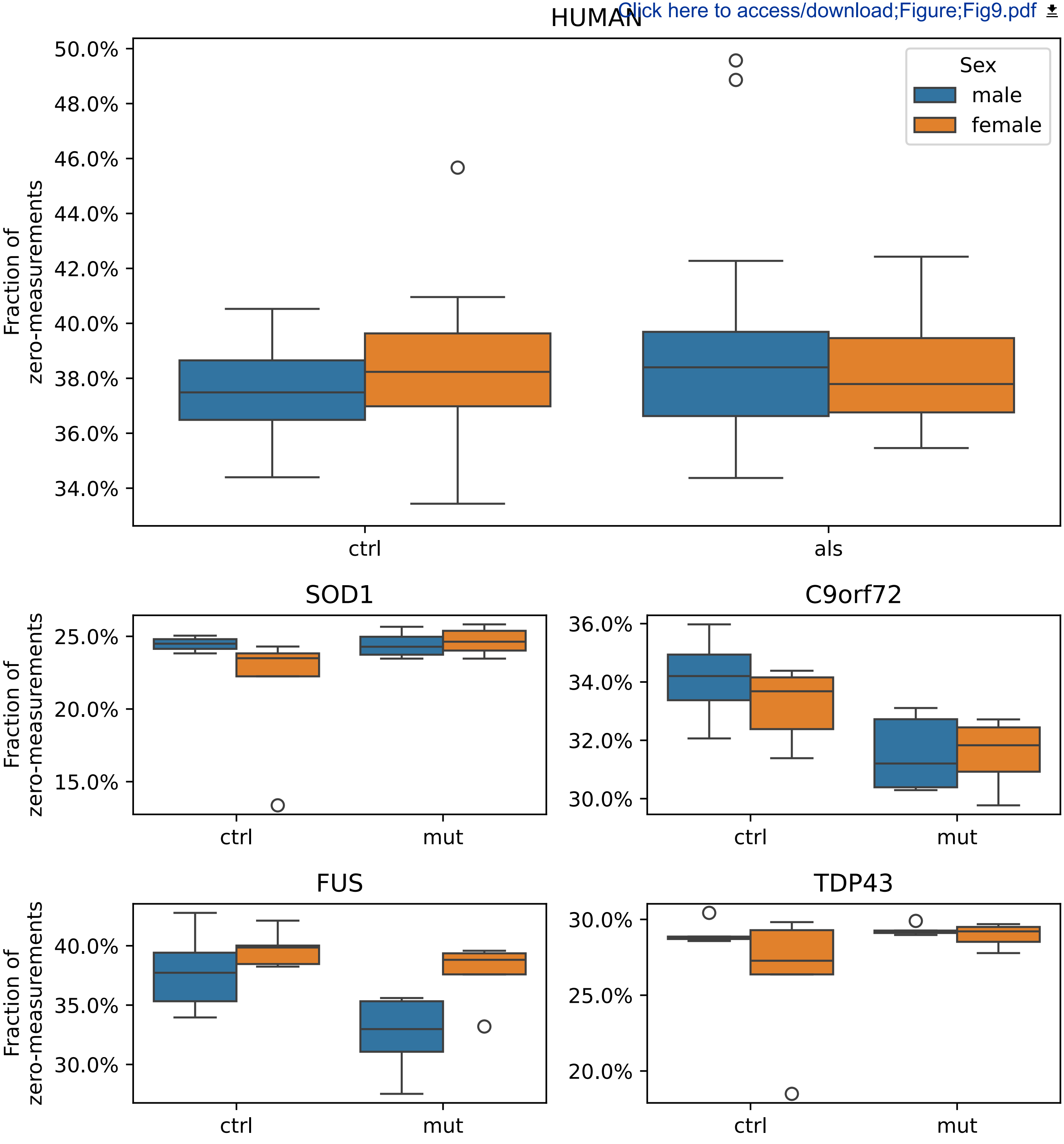

Figure

Click here to access/download;Figure;Figure10.pdf

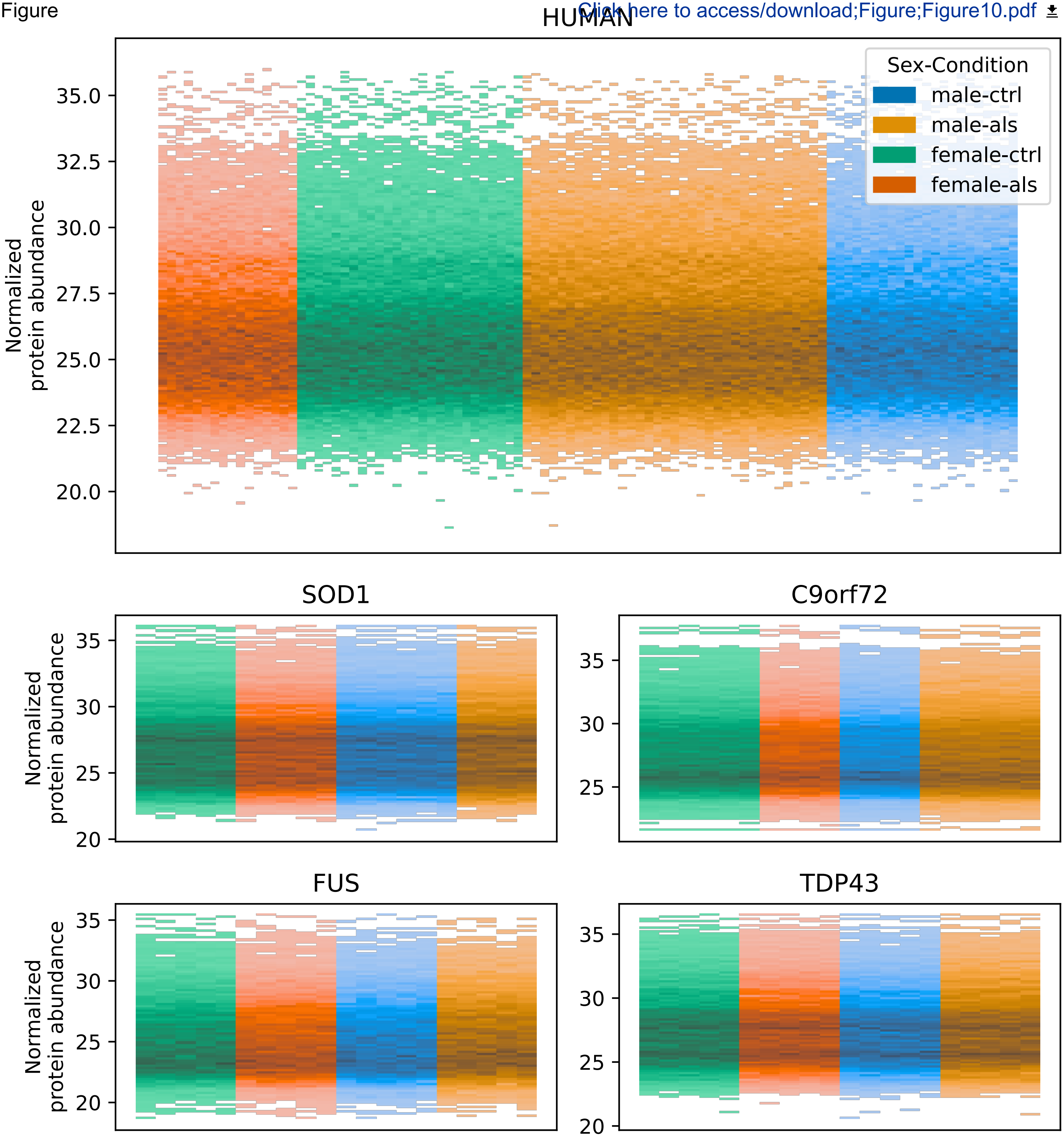

Click here to access/download;Figure;Fig11.pdf

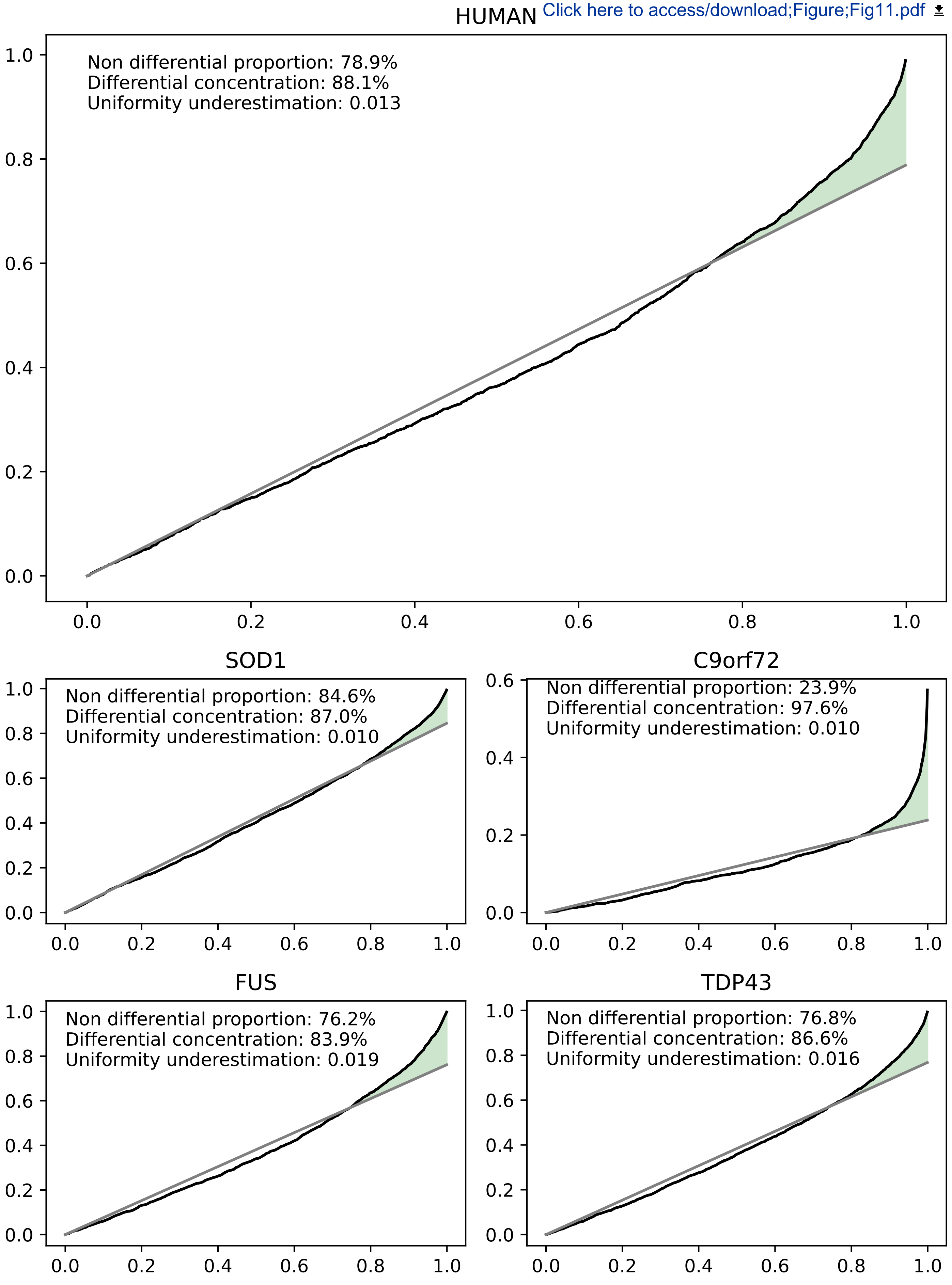

Figure

Click here to access/download;Figure;Figure12.pdf

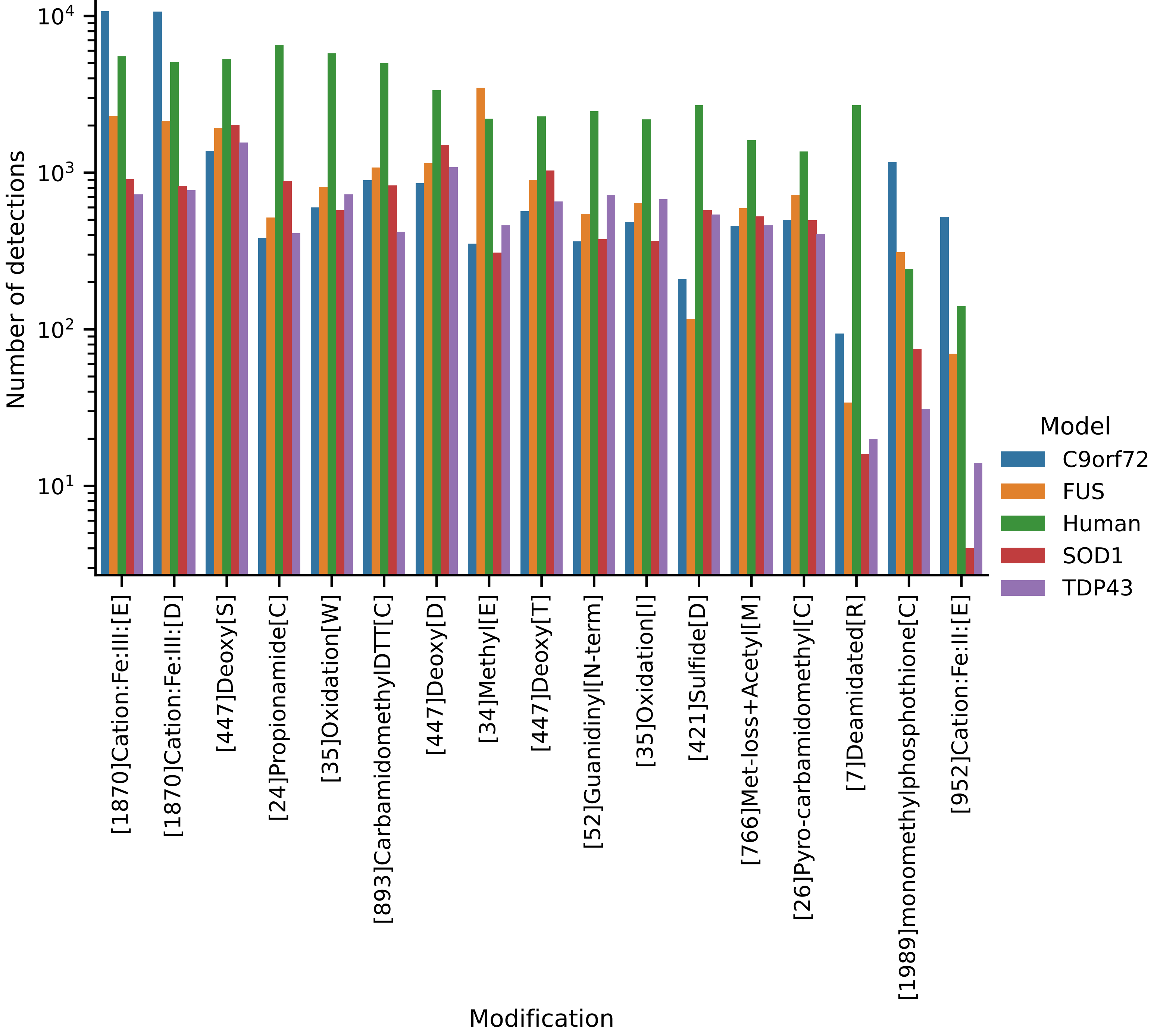

Figure
Click here to access/download;Figure;Figure13.pdf

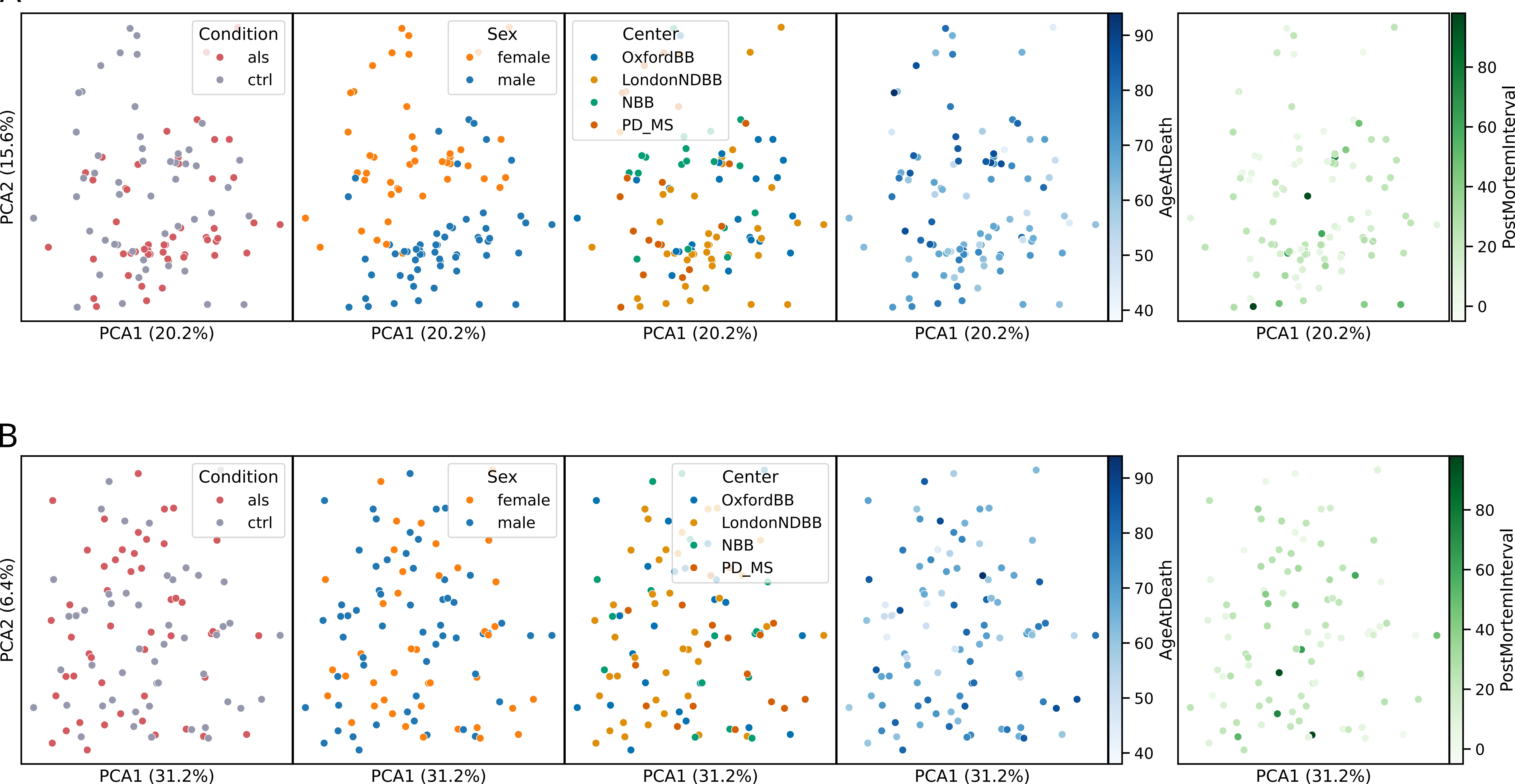

Click here to access/download;Personal Cover;Rebuttal Letter GigaScience.docx

Rebuttal Letter for GIGA-D-24-00236

A Data Set Profiling the Multi-omic Landscape of the Prefrontal Cortex in Amyotrophic Lateral Sclerosis

Fabian Hausmann; Lucas Caldi Gomes; Sonja Hänzelmann; Robin Khatri; Sergio Oller; Mojan Parvaz; Laura Tzeplaeff; Laura Pasetto; Marie Gebelin; Qihui Zhou; Pavol Zelina; Dieter Edbauer; R. Jeroen Pasterkamp; Hubert Rehrauer; Ralph Schlapbach; Christine Carapito; Valentina Bonetto; Stefan Bonn; Paul Lingor

Note from the Editor

Dear Prof Lingor,

Your manuscript "A Data Set Profiling the Multi-omic Landscape of the Prefrontal Cortex in Amyotrophic Lateral Sclerosis" (GIGA-D-24-00236) has been assessed by our reviewers. Although it is of interest, we are unable to consider it for publication in its current form. The reviewers have raised a number of points which we believe would improve the manuscript and may allow a revised version to be published in GigaScience.

Their reports, together with any other comments, are below. Please also take a moment to check our website at https://www.editorialmanager.com/giga/ for any additional comments that were saved as attachments.
In addition, please register any new software application in the bio.tools and SciCrunch.org databases to receive RRID (Research Resource Identification Initiative ID) and biotoolsID identifiers, and include these in your manuscript. Computational workflows should be registered in workflowhub.eu and the DOIs cited in the relevant places in the manuscript. These will facilitate tracking, reproducibility and re-use of your tool.

If you are able to fully address these points, we would encourage you to submit a revised manuscript to GigaScience. Once you have made the necessary corrections, please submit online at: https://www.editorialmanager.com/giga/
If you have forgotten your username or password please use the "Send Login Details" link to get your login information. For security reasons, your password will be reset.

Please include a point-by-point within the 'Response to Reviewers' box in the submission system. Please ensure you describe additional experiments that were carried out and include a detailed rebuttal of any criticisms or requested revisions that you disagreed with. Please also ensure that your revised manuscript conforms to the journal style, which can be found in the Instructions for Authors on the journal homepage. If the data and code has been modified in the revision process please be sure to update the public versions of this too.

The due date for submitting the revised version of your article is 04 Nov 2024.
I look forward to receiving your revised manuscript soon.
Best wishes,
Hongling Zhou
GigaScience

Reviewer reports:

Reviewer #1: This "Data Note" appears to be a well-written introduction to the dataset that was used to write citation 12 (i.e. https://doi.org/10.1038/s41467-024-49196-y). In order to evaluate this Data Note I retrieved the journals stated purpose for such article types and have included the pertinent details below:
One of the aims of a Data Note is to incentivize and more rapidly release data before subsequent detailed analysis has been carried out. We do also publish Data Notes in coordination with or after the publication of an analysis paper, but we expect the Data Note to add value, especially in cases where the analysis paper has already been published. Data Notes should include significant additional detail that might not have been appropriate in the research paper, including information on data collection, detailed data validation, and information on exactly how these data can be re-used.
As the article has been published and the data made available, I would like to see the second part of the paragraph, an inclusion of significant additional detail, before publication, if that is what the authors and editor decide.

Answer: We thank the reviewer for this comment about reusability and agree that this is a very important aspect of this paper. We want to stress the point that none of the quality control and data validation procedures presented here are described in or are part of the original publication. We also present complete new datasets (e.g., open modification searches covering post translational modifications with the proteomics data). To further increase the reuse potential of the dataset we added three new sections about possible analyses and provided code to execute these, in addition to our previous section about the reuse potential.

I had a few questions related to the Data Note on its own that I would like to see addressed.

1) I applaud the authors focus on sporadic ALS. As the authors point out, a subset of sALS patients harbor disease causing mutations citing references 2 and 3. Did you find any mutations associated with these cases? If so which and where? I'm also a little uncertain as to why the mouse models are the best method of interrogating the differences between sALS and gALS. Please expand on the utility of these specific mouse models and why you chose these models over gALS cases from the 4 brain banks.

Answer: We thank the reviewer for their thorough review and insightful comments. As explained in our original publication (Caldi Gomes & Hänzelmann et al., 2024, Nature Communications), we conducted a comprehensive genetic assessment of our ALS cohort using a gene panel analysis. This genetic screening included 30 major ALS-associated genes, and the findings indicated that the cohort predominantly consists of sporadic cases: only 2 out of the 51 ALS cases analyzed presented genetic alterations (one *C9orf72* repeat expansion and one pathogenic variant of *NEK1* [c.3107C>G, p.Ser1036Ter] were detected).

As there are currently no mouse models available that replicate sporadic ALS, we selected transgenic SOD1, TDP43, C9orf72, and FUS mouse models based on the importance and prevalence of these causative mutations in ALS. These models represent the most common ALS-causing mutations and provide a broad spectrum of disease mechanisms to study. Our choice was also influenced by the need to utilize well-characterized models that offer reproducibility and a wealth of existing data for comparison. The SOD1-G93A mouse model, for example, is the most-commonly used mouse model for ALS and provides a valuable benchmark for comparative studies.

Although sALS and gALS present differences in their etiology, both exhibit common key pathological features. These include, for example, mitochondrial dysfunction, neuroinflammation, and protein aggregation (PMID: 28871262, PMID: 32854276, PMID: 27830784). Indeed, up to 97% of all ALS patients

(including sALS and gALS) demonstrate TDP43 pathology that can be reproduced in transgenic mice (https://doi.org/10.2217/fnl.10.47 ; PMID: 37638324; PMID: 32854276). While the selected models are based on specific genetic mutations often associated with familial ALS, they are very valuable for studying the broader mechanisms that are also relevant to sALS. All four models used here are known to exhibit key pathological features shared between gALS and sALS. In this way, they serve as powerful tools for investigating the underlying mechanisms contributing to motoneuron degeneration, regardless of whether the ALS is of genetic or sporadic form.

Thus, studying these models can provide insights that apply to both forms of the disease. Our data strongly suggests that each of the genetic mouse models used, represents a subgroup of sporadic ALS patients. In conclusion, although the mouse models employed here are based on genetic mutations, their ability to elucidate shared pathogenic pathways in ALS justified their use in our study of sporadic ALS.

2) I am also a little dubious as to the justification of PFC as having signs of early alterations that are absent in other brain regions post mortem. I would like to see additional evidence that the authors may not have had space to include in the original publication.

Answer: We appreciate the reviewer's request for further clarification regarding our focus on the prefrontal cortex (PFC) in ALS. Our decision to examine the PFC (particularly Brodmann area 6) is based on both pathological staging and the search for early disease-related alterations that may be less apparent in regions more severely affected by ALS at the time of death. Moreover, post-mortem motor cortex and spinal cord have already been extensively studied and described in the disease, but as most-severely affected regions are strongly biased by end-stage alterations.

As extensively characterized by Brettschneider et al. (2013) (PMID: 23686809), the spread of TDP-43 inclusions follows a distinct pattern: it shows a rostrocaudal progression, starting in the motor cortex and subsequently spreading to other cortical and subcortical areas. By the end stage of ALS, the motor cortex shows extensive degeneration and gliosis, which obscures the identification of early molecular changes due to the overwhelming presence of advanced and rather unspecific pathology.
With our multiomics studies in the PFC, we aimed to circumvent the description of end-stage pathology. Instead, we tried to capture molecular changes that occur before widespread neuronal death/associated cellular and tissue changes, providing an overview of earlier disease mechanisms. Being affected only later in the disease course, the PFC is deemed to exhibit only intermediate levels of TDP-43 pathology at the time of death, possibly harboring early signs of disease-related alterations that are not as evident in more severely affected brain regions. It is worth noting that the findings of Brettschneider et al. (2013) have been consistently supported by subsequent studies, reinforcing the understanding that the prefrontal cortex is affected later in the disease course.

As requested by the reviewer, we present an overview of some of the studies that provide further evidence for the findings described in Brettschenider et al:
A study by Kassubek et al. (2014) used diffusion tensor imaging to demonstrate that ALS-related axonal damage in white-matter tracts affected the same regions as reported in the TDP-43 staging study of Brettschneider et al (PMID: 24736303). Moreover, a recent study using an ALS mouse model demonstrated that TDP-43-mediated pathology spreads through corticofugal tracts affected in ALS (PMID: 37011806). Another study investigated the TDP-43 spreading pattern in transgenic mice, showing that injecting TDP-43 preformed fibrils (PFFs) into the motor cortex resulted in the prion-like spread of TDP-43 pathology along the pyramidal tract. Over time, this led to widespread TDP-43 pathology throughout various brain regions, confirming the prion-like transmission of TDP-43 in ALS (PMID: 33461623).
Finally, a recent perspective paper (PMID: 32157757) compiled evidence from several studies that reinforce the anatomical spreading and propagation hypothesis of TDP-43 pathology in ALS-affected patients, as

proposed by Brettschneider et al. Overall, these studies provide further evidence that the prefrontal cortex (PFC) is affected only later in the disease course, offering insights into the early mechanisms of ALS and potential intervention points.

In summary, by focusing on the PFC, we aim to uncover molecular changes that represent early, pre-symptomatic stages of ALS. Understanding these changes could provide critical insights into the initial pathogenic events that precede widespread neurodegeneration.

3) In the data preparation please include the number of files, fractionation, number of peptides, and total number of proteins and or transcripts.

Answer: As requested by the reviewer, we added in the data preparation section in more detail the number of detected transcripts, sRNAs and detected proteins (Table 3 and below). The sequencing experiments resulted in one FASTQ file per sample, e.g. 20 FASTQ files for SOD1 transcriptomics data.

**Model**	**Transcriptomics**	**Proteomics**	**sRNA (mature/hairpin)**
**Human**	19641	2363	736 (224/512)
**SOD1 - Mouse**	16583	2854	893 (526/367)
**TDP43 - Mouse**	16801	2802	907 (534/373)
**C9orf72 - Mouse**	17465	2866	754 (271/483)
**FUS - Mouse**	17230	2522	812 (468/344)

4) I would like to see additional searches for modifications to round out the inclusion of additional information about this dataset (for proteomics) that may not have fit in the original paper.

Answer:
Thank you for the suggestion. We performed an open modification search using ionbot and the results are available at 10.6084/m9.figshare.27108562 for re-use by other researchers. In addition, we added the newly created Figure 12 (shown below) as an overview of the results of the open modification search in the main manuscript.

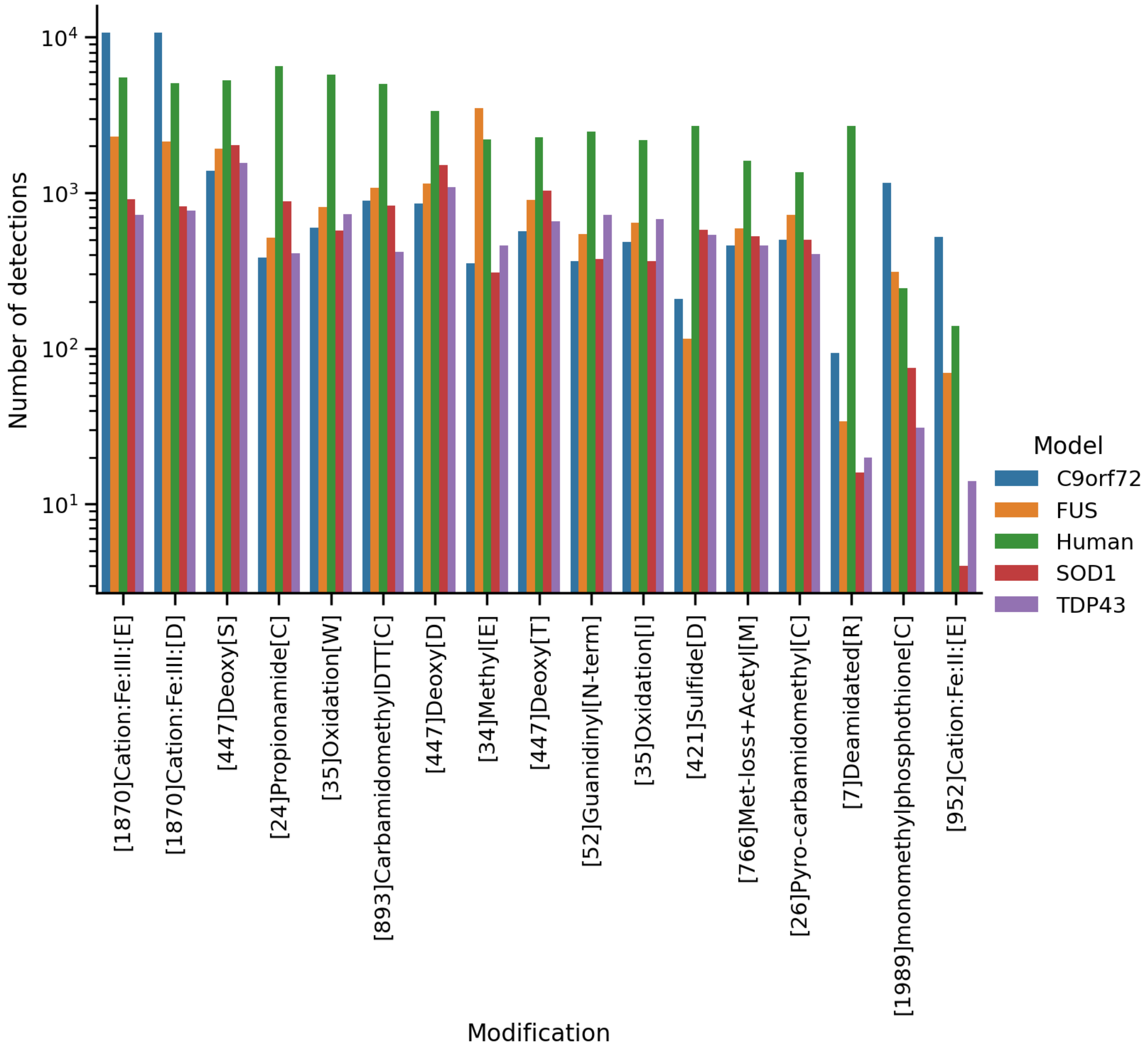

Figure legend: Top modifications found by the open modification search using ionbot for the four mouse models and human samples. For each model, the top 10 modifications were selected and the number of occurrences of the union of those (17 modifications) is displayed. Fixed modifications (Carbamidomethyl, Oxidation, Acetyl[N-term]) and sequence variations (Glu->Ser, Arg->Orn, Ser->Ala, Gln->pyro-Glu, Xle->Pro, Tyr->Phe, Delta:H(2)C(2)[N-term]) were removed for display.

The following methods description was added the methods part of the manuscript:

“In addition, an open modification search was performed. MGF files from the mouse and human proteomics data were loaded into IonBot (https://ionbot.cloud/) software (v. 0.11.0). Provided databases were used, either human (9606 entries) or mus musculus (10090 entries), with a K|R cleavage pattern. Error tolerances were set on default values: MS precursor tolerance at 20 ppm and MS/MS fragment tolerance at 0.02 Da. Methionine oxidation and protein N-term acetylation were set as variable modifications, while cysteine carbamidomethylation as a fixed modification. Open modification search option was enabled.”

Well done getting this information together, it was clearly a lot of work. Following major revisions I would be interested in seeing this note again.

Reviewer #2:
Major comments:
The authors have presented a useful omics resource for ALS studies, which could be used to identify biomarkers and drug targets for ALS treatments,. However, the following points should be addressed.

1. Although the authors have described differential expression and enrichment analyses, as well as proteomics data analysis in their methods section, they did not report any of these results in the manuscript. Including these findings is crucial for providing a comprehensive understanding of the data and its implications. The absence of these results limits the ability of readers to fully assess the scope and impact of the analyses performed. I recommend that the authors include detailed results from these analyses to enhance the manuscript's completeness and clarity.

Answer: We thank the reviewer for the constructive suggestions. We would like to clarify that the original multiomics data, as well as the functional interpretation for all datasets and models, have been extensively detailed in our prior publication in Nature Communications (https://doi.org/10.1038/s41467-024-49196-y). With our submission of this Data Note manuscript to GigaScience, we aimed at providing better documentation, improving data accessibility and reuse for this comprehensive set of data. We believe that revisiting these findings, describing yet again the main results to draw functional conclusions might lead to redundancy and detract from the primary aim of a data note article.

To improve clarity, we decided to provide a summary of our main results here, and also included a new paragraph for this purpose in our *Context* section for the current submission. In brief, in our initial study, we conducted a multiomic analysis of PFC tissue, revealing distinct molecular subclusters within ALS patients. These subclusters showed varying patterns in gene, protein and miRNA expression, suggesting the presence of different underlying disease mechanisms. These findings underscore the need for personalized therapeutic approaches. Another important aspect of our study was the identification of pronounced sex differences captured in the molecular profiles of ALS patients, with male patients exhibiting more pronounced alterations overall.

Our study emphasized and focused on the MAPK pathway as a critical therapeutic target. The involvement of this pathway suggests it could be a focal point for developing targeted treatments, which could improve the prognosis for ALS patients. Other important pathways identified in our study were the activation of immune response, extracellular matrix composition, mitochondrial function, and RNA processing.

In parallel, we have analyzed four different mouse models, bearing the most common ALS-causing mutations. The results from human analyses were corroborated in ALS mouse models, which exhibited similar molecular patterns, and partially resembled the human subclusters revealed through the analysis of human brain tissue. This cross-species validation strengthens the relevance of the identified subclusters and pathways as potential therapeutic targets.

The findings summarized here were validated across multiple datasets, reinforcing the significance of the identified molecular sub clusters and pathways. This validation suggests that future ALS research should consider these frequently reported molecular differences, and focus on developing personalized medicine approaches, tailored to specific patient subgroups.

We hope that this clarifies the points put forward by the reviewer, as well as the intent and scope of the present manuscript.

2. The authors employed DESeq2 for identifying differentially expressed genes (DEGs) and miRNAs between experimental conditions (ALS vs. controls). While DESeq2 is a robust tool for differential expression analysis, it has limitations, particularly in handling low-count genes and in estimating dispersion in small sample sizes.

I would suggest the authors perform DEGs using the limma/voom approach. Limma, with its voom transformation, can provide better performance in terms of handling heteroscedasticity and is particularly effective for RNA-Seq data with varied library sizes. It also offers more accurate estimation of mean-variance relationships, which can improve the detection of true DEGs.

Additionally, using limma/voom can complement DESeq2 results and provide a more comprehensive analysis by cross-validating the findings. This dual approach would enhance the robustness and reliability of the differential expression analysis, potentially uncovering more biologically relevant insights.

Answer: We thank the reviewer for these suggestions. While limma/voom have been adapted for RNA-Seq data, it is important to highlight that limma was originally developed for microarray data. The voom transformation referred by the reviewer allows limma to handle RNA-Seq data by converting raw counts into log2 counts per million (log-CPM). However, this additional layer of modeling may not always capture the unique characteristics of RNA-Seq data as effectively as DESeq2. Limma-voom was originally only tested against the previous version, DESeq, and not against DESeq2. Furthermore, in a recent benchmark by Li et al. (2022) [PMID: 36112652] DESeq2 remains the recommended method for most use cases with higher sample sizes (>6).

DESeq2, on the other hand, implements independent filtering and shrinkage estimation of dispersion, which makes it particularly robust in handling low-count genes — a common challenge in RNA-Seq data analysis. These features help minimize some false positives, ensuring more accurate results for low-count genes. Moreover, DESeq2’s robust statistical framework, including its use of a negative binomial distribution, accounts for variability between experimental conditions. This makes DESeq2 especially well-suited for studies with expected variability, such as those comparing ALS patients to controls.

As requested by the reviewer, we have performed comparative analyses for both methods and the human samples and found that, in general, DESeq2 detected more differentially expressed genes than limma/voom - especially for males, which encompassed the most changes captured in our datasets (Venn diagrams). This is also the case for our proteomics samples (379 DEPs in males vs 251 DEPs in females) where we didn’t use DESeq2 but limma (without voom) for our analysis.

While it can be valuable to use limma/voom as a complementary approach to validate DESeq2 findings, in our view, DESeq2 should remain the primary method due to its direct modeling of RNA-Seq data, and its handling of biological variability, low-count genes, the high detection rate for exploratory research and its consistency with the proteomics data. Employing both methods could enhance the robustness of the analysis, but DESeq2's approach is likely to yield more comprehensive primary results, particularly in our context.

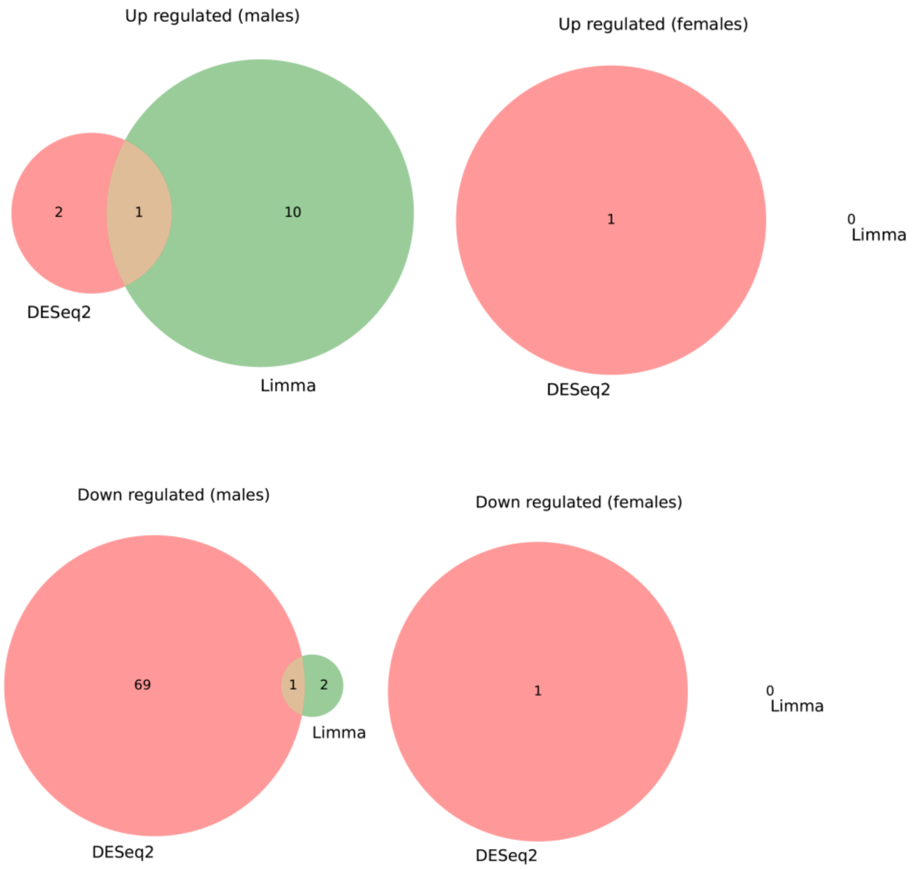

3. The authors should provide a detailed description of how they adjusted for potential confounding variables in their DEGs and proteomics analyses analysis. Specifically, it is important to describe the methods used to account for batch effects, principal components of gene/protein expression, and the effects of age and sex on their analyses. Proper adjustment for these variables is crucial for ensuring the validity and reliability of the results.

Answer: We used PCA and a sample distance heatmap to check for batch effects in our transcriptomics and proteomics analyses. We could not detect any batch effects in our proteomics and transcriptomics data and exemplify this in the transcriptomics data below. We specifically investigated batch effects related to the factors brain bank, sex, case/control condition, as well as age at death. However, we identified marked sex-related differences (Figure 1b of Caldi Gomes, Hänzelmann et al., 2024; shown below) and therefore, all analyses were performed separately for each sex.

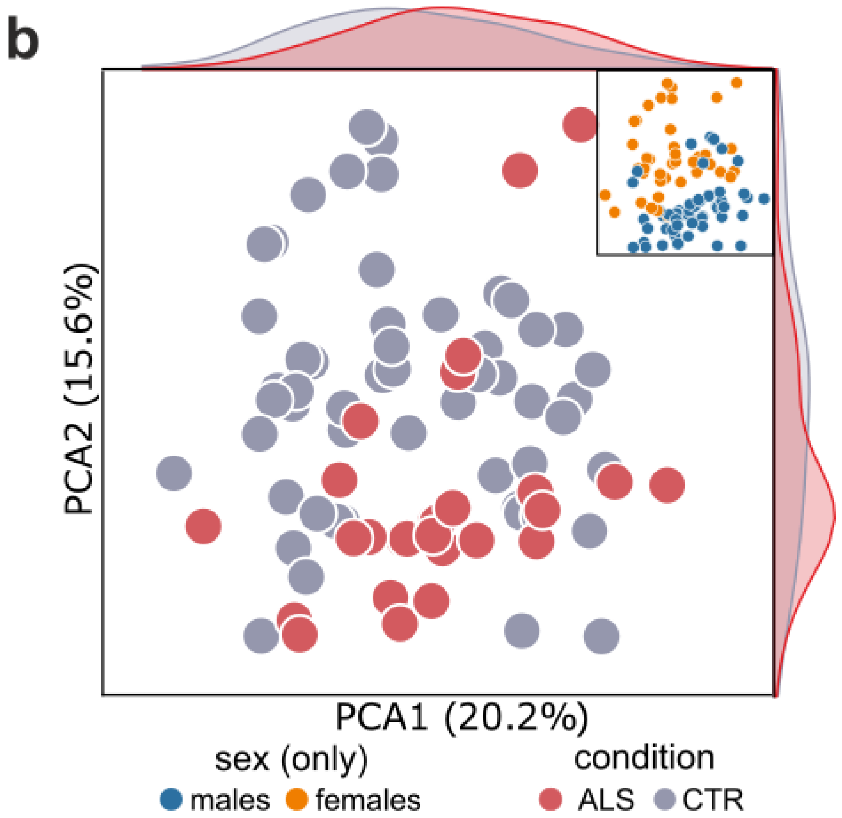

For the other factors, we found no evidence that they influenced the results (Supplementary Figure 1 of Caldi Gomes, Hänzelmann et al., 2024; shown below). For instance, if batch effects had been present, they would likely have caused distinct clustering or separation of samples. However, our analysis showed no such patterns, indicating that these factors did not introduce systematic bias to our data.

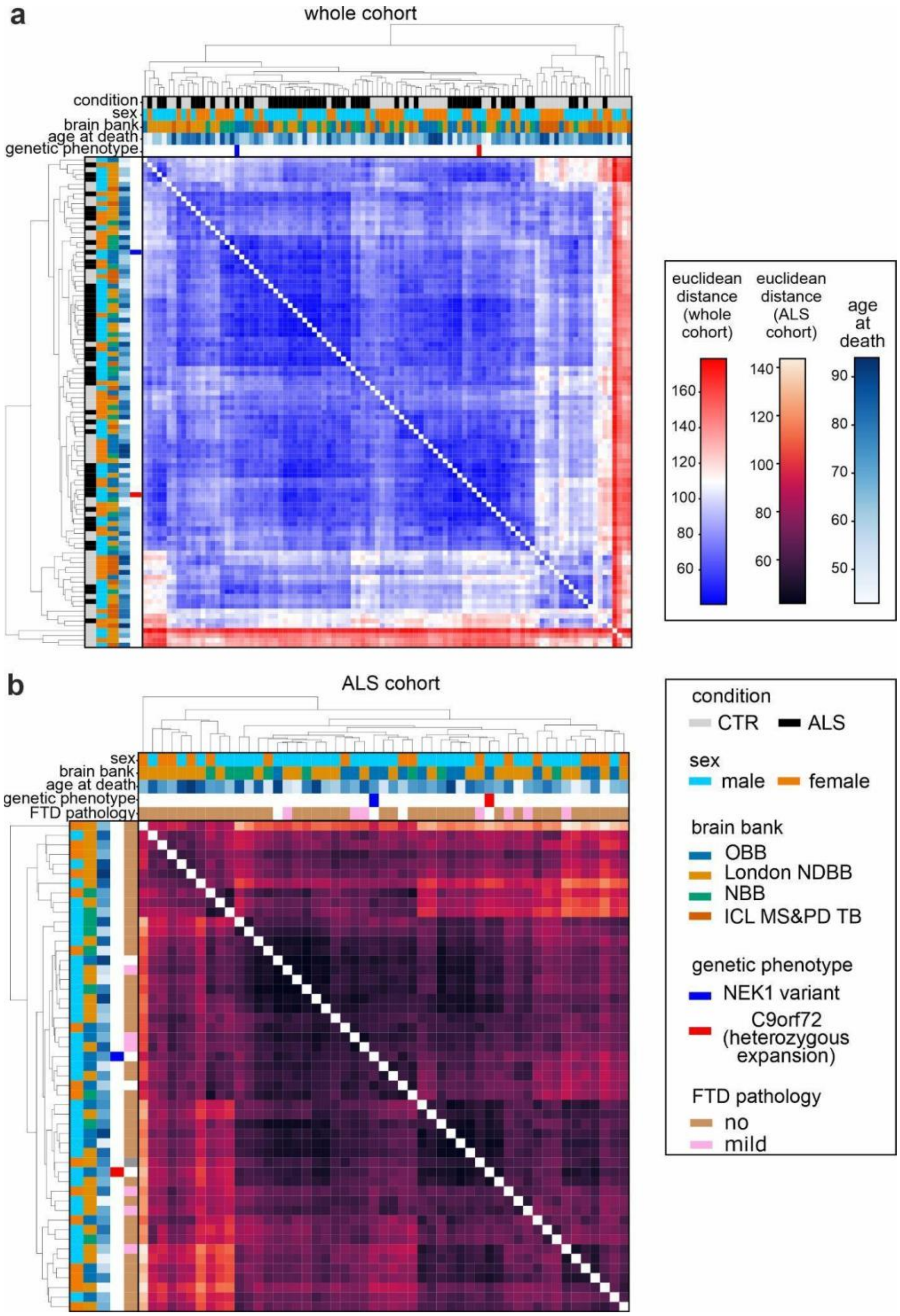

Furthermore, we could not detect any influence of the post-mortem interval on the expression of known neuronal cell death markers, indicating measurable influence by the sample handling (Supplementary Figure 4a of Caldi Gomes, Hänzelmann et al., 2024; shown below).

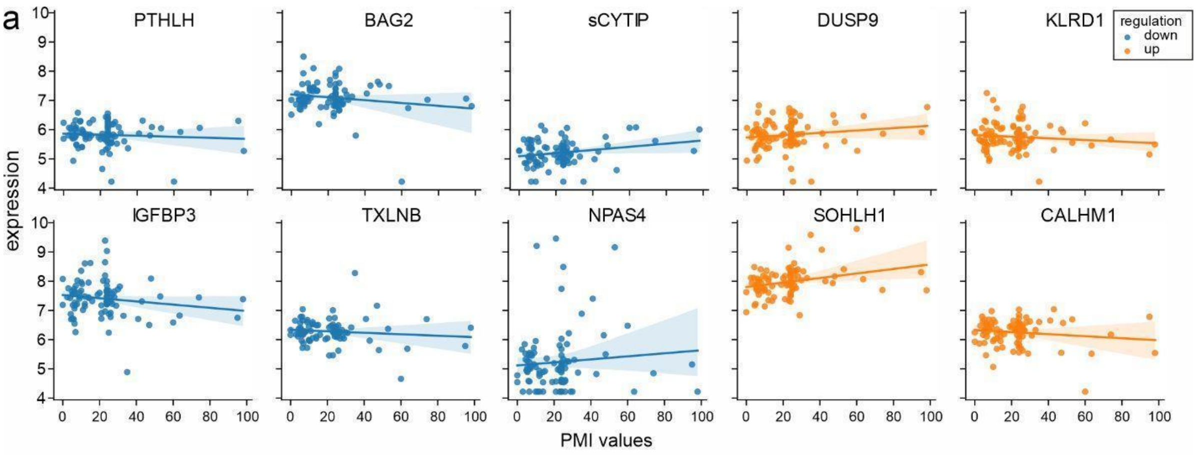

We now discuss this in a newly added section of the manuscript and added Figure 13 showing all known factors in the PCA representation for the transcriptomic (A) and proteomics (B) data.

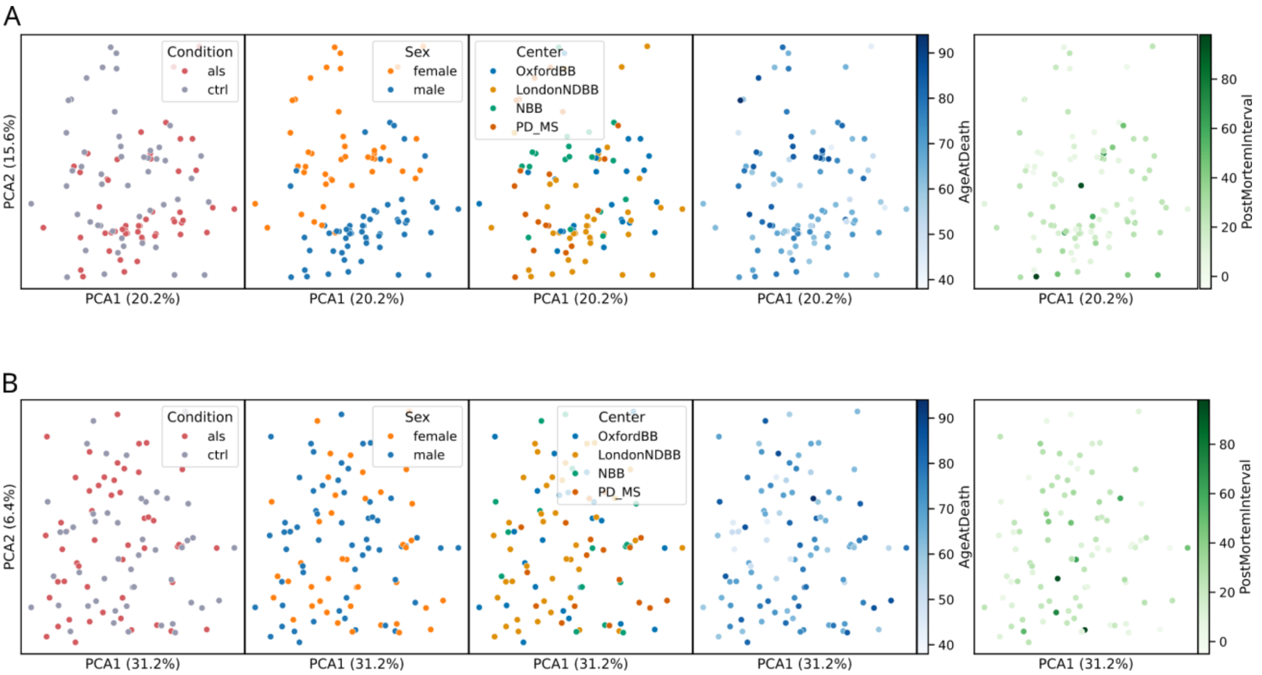

Minor comments:

1. The authors should describe whether their omics data consist of single-end or paired-end reads.

Answer: Single-end reads. We made sure that this information is more explicitly stated in the methods.

2. The color scheme in Figure 6 makes it difficult to differentiate between cases and controls, particularly male-ctrl and male-als. I recommend that the authors revise the color scheme to improve contrast and distinguishability.

Answer: We thank the reviewer for the comment and agree with the color choice change. We resorted to a color-blind-friendly palette now and adjusted the respective figures.

--
Please also take a moment to check our website at https://www.editorialmanager.com/giga/l.asp?i=191766&l=V4LNH38F for any additional comments that were saved as attachments. Please note that as GigaScience has a policy of open peer review, you will be able to see the names of the reviewers.

No additional files for our submission.

Appendix – Response to editorial request

E-Mail from the editorial office:

Dear Dr Lingor,
Thank you for your submission to GigaScience.

During the review process we collect author ORCID iDs (https://orcid.org/) for all of the co-authors on the manuscript, should the manuscript ultimately be approved for publication. This ensures we are able to link manuscripts submitted to GigaScience by their ORCID Identifier, thus enabling a better, more cohesive and distinguished search of our authors and their papers.
Please note, if authors do not have an ORCID iD, it is not compulsory to create one. For authors who have ORCID iDs, please adhere to the following format:

First, Middle and Last Name, proceeded by the ORCID iD in square brackets
John A Doe [0000-1234-5678-0000]

Answer:
Fabian Hausmann [0000-0001-6110-5824]; Lucas Caldi Gomes [0000-0003-4959-2169]; Sonja Hänzelmann [0000-0003-4953-0101]; Robin Khatri [0009-0006-5311-1718]; Sergio Oller [0000-0002-8994-1549]; Mojan Parvaz [0000-0002-0644-5559]; Laura Tzeplaeff [0009-0003-7643-3232]; Laura Pasetto [0000-0003-0319-4448]; Marie Gebelin [0009-0001-4696-3799]; Qihui Zhou [0000-0001-5733-9820]; Pavol Zelina [0009-0008-6776-663X]; Dieter Edbauer [0000-0002-7186-4653]; R Jeroen Pasterkamp [0000-0003-1631-6440]; Hubert Rehrauer [0000-0001-7612-9394]; Ralph Schlapbach [0000-0002-7488-4262]; Christine Carapito [0000-0002-0079-319X]; Valentina Bonetto [0000-0003-0456-2054]; Stefan Bonn [0000-0003-4366-5662]; Paul Lingor [0000-0001-9362-7096];

Please also clarify for me the funding section of your manuscript. The format to adhere to (for the purposes of this email only) is: Funding body, Program/Award name, award ID, Recipient; e.g. Wellcome Trust, Biomedical Resources Grant, 12345/Z/, R E Franklin
I have for your convenience formatted the funding section as per your submission. Please do confirm whether this is correct. You may send both the formatted funding, and the ORCID iDs to me at this address.

Answer:
BMBF, MAXOMOD, 01GM1917A, P Lingor;
Munich Cluster for Systems Neurology, P Lingor;
DFG, Immune-Mediated Glomerular Diseases – Basic Concepts and Clinical Implications, CRC1192, S Hänzelmann;
BMBF, STOP-FSGS, 01GM2202A, S Hänzelmann;
DFG, Quantitative Synaptology, CRC1286, S Bonn;
BMBF, MAXOMOD, 01GM1917A, S Bonn;

Personal Cover

Click here to access/download;Personal Cover;Final - Cover Letter after revision.docx

Klinikum rechts der Isar · Klinik für Neurologie · 81675 München

DIE DEUTSCHEN UNIVERSITÄTSKLINIKA®

Klinikum rechts der Isar

Klinikum rechts der Isar
Anstalt des öffentlichen Rechts

Klinik und Poliklinik für Neurologie
Direktor: Prof. Dr. B. Hemmer

Prof. Dr. Paul Lingor
Oberarzt

Ismaninger Straße 22
81675 München
E-Mail: paul.lingor@tum.de
Tel: 089 4140-8257
Fax: 089 4140-4867

Technische Universität München

Vorstand:
Dr. Martin Siess
(Ärztlicher Direktor, Vorsitzender)

Marie le Claire
(Kaufmännische Direktorin)

Silke Großmann
(Pflegedirektorin)

Prof. Dr. Stephanie E. Combs
(Dekanin)

Bankverbindung:
Bayer. Landesbank Girozentrale

BIC: BYLADEMM
IBAN: DE82 7005 0000 0000 0202 72
USt-IdNr. DE 129 52 3996

To the Editor in Chief of *Giga Science*

Dr. Scott Edmunds

October 1st, 2024

Dear Dr. Edmunds,

I hope this letter finds you well. Thank you and the reviewers very much for the thorough review of the manuscript titled "*A Data Set Profiling the Multi-omic Landscape of the Prefrontal Cortex in Amyotrophic Lateral Sclerosis*", submitted as a *Data Note* to *Giga Science*. The paper was co-authored by Fabian Hausmann, Lucas Caldi Gomes, Sonja Hänzelmann, Robin Khatri, Sergio Oller, Mojan Parvaz, Laura Tzeplaeff, Laura Pasetto, Marie Gebelin, Qihui Zhou, Pavol Zelina, Dieter Edbauer, R. Jeroen Pasterkamp, Hubert Rehrauer, Ralph Schlapbach, Christine Carapito, Valentina Bonetto and Stefan Bonn.

We greatly appreciate the opportunity to revise our work and have carefully considered all points of criticism put forward by the reviewers. In addition to textual and structural changes in the paper, we have included several additional manuscript sections, and also provided completely new analyses and datasets, as requested for this revision work. You will find our answers to the reviewer's questions in the point-by-point response attached to this submission. Our answers are marked by A: and blue text. We also would like to point out that there were slight changes in the authorship order, following the new contributions for the revision experiments, text, and figure changes.

As initially reported, this manuscript provides a comprehensive summary of the multi-omic analyses conducted as part of our study recently published by Nature Communications titled "Multiomic ALS signatures highlight subclusters and sex differences suggesting the MAPK pathway as therapeutic target" (https://doi.org/10.1038/s41467-024-49196-y). Our research resulted in unique datasets and a reproducible and extendable computational workflow that integrates multiple omics types to understand the molecular architecture of ALS in the PFC comprehensively. Our data description manuscript now includes detailed methodologies and quality control measures for RNA sequencing (mRNA and small RNA) and proteomics, as well as newly derived analyses and data sources from the original multi-omic data. This manuscript also emphasizes the importance of accessibility and reproducibility, providing extensive documentation of bioinformatics workflows and code to facilitate data reuse and transparency in analysis adhering to the FAIR (findable, accessible, interoperable, reproducible) principles.

With this revised submission, we believe we have strengthened the manuscript, enhancing its overall clarity and impact. We are confident that the detailed data description we present here will serve as a valuable resource for your journal's readership. We sincerely hope that the revised manuscript now meets the highest standards of *Giga Science.*

This manuscript has not been published or presented elsewhere in part or in entirety and is not under consideration by another journal. The study design was approved by the appropriate ethics review board. We have read and understood your journal's policies, and we believe that neither the manuscript nor the study violates any of these. There are no conflicts of interest to declare.

Thank you for your time and consideration, looking forward to hearing from you soon.

Yours sincerely,

Paul Lingor
(for all co-authors)
