# Supplementary material for: A dataset profiling the multiomic landscape of the prefrontal cortex in amyotrophic lateral sclerosis
Source: Gigascience. 2024 Dec 18;13:giae100. doi: 10.1093/gigascience/giae100 (PMC11653894; doi:10.1093/gigascience/giae100)
Supplement: giae100_Response_to_Reviewer_Comments_Original_Submission [file giae100_response_to_reviewer_comments_original_submission.pdf]

Author's Response To Reviewer Comments

Rebuttal Letter for GIGA-D-24-00236

A Data Set Profiling the Multi-omic Landscape of the Prefrontal Cortex in Amyotrophic Lateral Sclerosis
Fabian Hausmann; Lucas Caldi Gomes; Sonja Hänzelmann; Robin Khatri; Sergio Oller; Mojan Parvaz; Laura Tzeplaeff; Laura Pasetto; Marie Gebelin; Qihui Zhou; Pavol Zelina; Dieter Edbauer; R. Jeroen Pasterkamp; Hubert Rehrauer; Ralph Schlapbach; Christine Carapito; Valentina Bonetto; Stefan Bonn; Paul Lingor

Reviewer reports:

Reviewer #1: This "Data Note" appears to be a well-written introduction to the dataset that was used to write citation 12 (i.e. https://doi.org/10.1038/s41467-024-49196-y). In order to evaluate this Data Note I retrieved the journals stated purpose for such article types and have included the pertinent details below: One of the aims of a Data Note is to incentivize and more rapidly release data before subsequent detailed analysis has been carried out. We do also publish Data Notes in coordination with or after the publication of an analysis paper, but we expect the Data Note to add value, especially in cases where the analysis paper has already been published. Data Notes should include significant additional detail that might not have been appropriate in the research paper, including information on data collection, detailed data validation, and information on exactly how these data can be re-used.
As the article has been published and the data made available, I would like to see the second part of the paragraph, an inclusion of significant additional detail, before publication, if that is what the authors and editor decide.

Answer: We thank the reviewer for this comment about reusability and agree that this is a very important aspect of this paper. We want to stress the point that none of the quality control and data validation procedures presented here are described in or are part of the original publication. We also present complete new datasets (e.g., open modification searches covering post translational modifications with the proteomics data). To further increase the reuse potential of the dataset we added three new sections about possible analyses and provided code to execute these, in addition to our previous section about the reuse potential.

I had a few questions related to the Data Note on its own that I would like to see addressed.

1) I applaud the authors focus on sporadic ALS. As the authors point out, a subset of sALS patients harbor disease causing mutations citing references 2 and 3. Did you find any mutations associated with these cases? If so which and where? I'm also a little uncertain as to why the mouse models are the best method of interrogating the differences between sALS and gALS. Please expand on the utility of these specific mouse models and why you chose these models over gALS cases from the 4 brain banks.

Answer: We thank the reviewer for their thorough review and insightful comments. As explained in our original publication (Caldi Gomes & Hänzelmann et al., 2024, Nature Communications), we conducted a comprehensive genetic assessment of our ALS cohort using a gene panel analysis. This genetic screening included 30 major ALS-associated genes, and the findings indicated that the cohort predominantly consists of sporadic cases: only 2 out of the 51 ALS cases analyzed presented genetic alterations (one C9orf72 repeat expansion and one pathogenic variant of NEK1 [c.3107C>G, p.Ser1036Ter] were detected).

As there are currently no mouse models available that replicate sporadic ALS, we selected transgenic SOD1, TDP43, C9orf72, and FUS mouse models based on the importance and prevalence of these causative mutations in ALS. These models represent the most common ALS-causing mutations and provide a broad spectrum of disease mechanisms to study. Our choice was also influenced by the need to utilize well-characterized models that offer reproducibility and a wealth of existing data for comparison. The SOD1-G93A mouse model, for example, is the most-commonly used mouse model for ALS and provides a

valuable benchmark for comparative studies.

Although sALS and gALS present differences in their etiology, both exhibit common key pathological features. These include, for example, mitochondrial dysfunction, neuroinflammation, and protein aggregation (PMID: 28871262, PMID: 32854276, PMID: 27830784). Indeed, up to 97% of all ALS patients (including sALS and gALS) demonstrate TDP43 pathology that can be reproduced in transgenic mice (https://doi.org/10.2217/fnl.10.47 ; PMID: 37638324; PMID: 32854276). While the selected models are based on specific genetic mutations often associated with familial ALS, they are very valuable for studying the broader mechanisms that are also relevant to sALS. All four models used here are known to exhibit key pathological features shared between gALS and sALS. In this way, they serve as powerful tools for investigating the underlying mechanisms contributing to motoneuron degeneration, regardless of whether the ALS is of genetic or sporadic form.

Thus, studying these models can provide insights that apply to both forms of the disease. Our data strongly suggests that each of the genetic mouse models used, represents a subgroup of sporadic ALS patients. In conclusion, although the mouse models employed here are based on genetic mutations, their ability to elucidate shared pathogenic pathways in ALS justified their use in our study of sporadic ALS.

2) I am also a little dubious as to the justification of PFC as having signs of early alterations that are absent in other brain regions post mortem. I would like to see additional evidence that the authors may not have had space to include in the original publication.

Answer: We appreciate the reviewer's request for further clarification regarding our focus on the prefrontal cortex (PFC) in ALS. Our decision to examine the PFC (particularly Brodmann area 6) is based on both pathological staging and the search for early disease-related alterations that may be less apparent in regions more severely affected by ALS at the time of death. Moreover, post-mortem motor cortex and spinal cord have already been extensively studied and described in the disease, but as most-severely affected regions are strongly biased by end-stage alterations.

As extensively characterized by Brettschneider et al. (2013) (PMID: 23686809), the spread of TDP-43 inclusions follows a distinct pattern: it shows a rostrocaudal progression, starting in the motor cortex and subsequently spreading to other cortical and subcortical areas. By the end stage of ALS, the motor cortex shows extensive degeneration and gliosis, which obscures the identification of early molecular changes due to the overwhelming presence of advanced and rather unspecific pathology.
With our multiomics studies in the PFC, we aimed to circumvent the description of end-stage pathology. Instead, we tried to capture molecular changes that occur before widespread neuronal death/associated cellular and tissue changes, providing an overview of earlier disease mechanisms. Being affected only later in the disease course, the PFC is deemed to exhibit only intermediate levels of TDP-43 pathology at the time of death, possibly harboring early signs of disease-related alterations that are not as evident in more severely affected brain regions. It is worth noting that the findings of Brettschneider et al. (2013) have been consistently supported by subsequent studies, reinforcing the understanding that the prefrontal cortex is affected later in the disease course.

As requested by the reviewer, we present an overview of some of the studies that provide further evidence for the findings described in Brettschenider et al:
A study by Kassubek et al. (2014) used diffusion tensor imaging to demonstrate that ALS-related axonal damage in white-matter tracts affected the same regions as reported in the TDP-43 staging study of Brettschneider et al (PMID: 24736303). Moreover, a recent study using an ALS mouse model demonstrated that TDP-43-mediated pathology spreads through corticofugal tracts affected in ALS (PMID: 37011806). Another study investigated the TDP-43 spreading pattern in transgenic mice, showing that injecting TDP-43 preformed fibrils (PFFs) into the motor cortex resulted in the prion-like spread of TDP-43 pathology along the pyramidal tract. Over time, this led to widespread TDP-43 pathology throughout various brain regions, confirming the prion-like transmission of TDP-43 in ALS (PMID: 33461623).
Finally, a recent perspective paper (PMID: 32157757) compiled evidence from several studies that reinforce the anatomical spreading and propagation hypothesis of TDP-43 pathology in ALS-affected patients, as proposed by Brettschneider et al. Overall, these studies provide further evidence that the prefrontal cortex (PFC) is affected only later in the disease course, offering insights into the early

mechanisms of ALS and potential intervention points.

In summary, by focusing on the PFC, we aim to uncover molecular changes that represent early, pre-symptomatic stages of ALS. Understanding these changes could provide critical insights into the initial pathogenic events that precede widespread neurodegeneration.

3) In the data preparation please include the number of files, fractionation, number of peptides, and total number of proteins and or transcripts.

Answer: As requested by the reviewer, we added in the data preparation section in more detail the number of detected transcripts, sRNAs and detected proteins (Table 3 and below). The sequencing experiments resulted in one FASTQ file per sample, e.g. 20 FASTQ files for SOD1 transcriptomics data.

Model Transcriptomics Proteomics sRNA (mature/hairpin)
Human 19641 2363 736 (224/512)
SOD1 - Mouse 16583 2854 893 (526/367)
TDP43 - Mouse 16801 2802 907 (534/373)
C9orf72 - Mouse 17465 2866 754 (271/483)
FUS - Mouse 17230 2522 812 (468/344)

4) I would like to see additional searches for modifications to round out the inclusion of additional information about this dataset (for proteomics) that may not have fit in the original paper.

Answer:
Thank you for the suggestion. We performed an open modification search using ionbot and the results are available at 10.6084/m9.figshare.27108562 for re-use by other researchers. In addition, we added the newly created Figure 12 (shown below) as an overview of the results of the open modification search in the main manuscript.
Figure legend: Top modifications found by the open modification search using ionbot for the four mouse models and human samples. For each model, the top 10 modifications were selected and the number of occurrences of the union of those (17 modifications) is displayed. Fixed modifications (Carbamidomethyl, Oxidation, Acetyl[N-term]) and sequence variations (Glu->Ser, Arg->Orn, Ser->Ala, Gln->pyro-Glu, Xle->Pro, Tyr->Phe, Delta:H(2)C(2)[N-term]) were removed for display.

The following methods description was added the methods part of the manuscript:

“In addition, an open modification search was performed. MGF files from the mouse and human proteomics data were loaded into IonBot (https://ionbot.cloud/) software (v. 0.11.0). Provided databases were used, either human (9606 entries) or mus musculus (10090 entries), with a K|R cleavage pattern. Error tolerances were set on default values: MS precursor tolerance at 20 ppm and MS/MS fragment tolerance at 0.02 Da. Methionine oxidation and protein N-term acetylation were set as variable modifications, while cysteine carbamidomethylation as a fixed modification. Open modification search option was enabled.”

Well done getting this information together, it was clearly a lot of work. Following major revisions I would be interested in seeing this note again.

Reviewer #2:
Major comments:
The authors have presented a useful omics resource for ALS studies, which could be used to identify biomarkers and drug targets for ALS treatments,. However, the following points should be addressed.

1. Although the authors have described differential expression and enrichment analyses, as well as proteomics data analysis in their methods section, they did not report any of these results in the manuscript. Including these findings is crucial for providing a comprehensive understanding of the data and its implications. The absence of these results limits the ability of readers to fully assess the scope and impact of the analyses performed. I recommend that the authors include detailed results from these

analyses to enhance the manuscript's completeness and clarity.

Answer: We thank the reviewer for the constructive suggestions. We would like to clarify that the original multiomics data, as well as the functional interpretation for all datasets and models, have been extensively detailed in our prior publication in Nature Communications (https://doi.org/10.1038/s41467-024-49196-y). With our submission of this Data Note manuscript to GigaScience, we aimed at providing better documentation, improving data accessibility and reuse for this comprehensive set of data. We believe that revisiting these findings, describing yet again the main results to draw functional conclusions might lead to redundancy and detract from the primary aim of a data note article.

To improve clarity, we decided to provide a summary of our main results here, and also included a new paragraph for this purpose in our Context section for the current submission. In brief, in our initial study, we conducted a multiomic analysis of PFC tissue, revealing distinct molecular subclusters within ALS patients. These subclusters showed varying patterns in gene, protein and miRNA expression, suggesting the presence of different underlying disease mechanisms. These findings underscore the need for personalized therapeutic approaches. Another important aspect of our study was the identification of pronounced sex differences captured in the molecular profiles of ALS patients, with male patients exhibiting more pronounced alterations overall.

Our study emphasized and focused on the MAPK pathway as a critical therapeutic target. The involvement of this pathway suggests it could be a focal point for developing targeted treatments, which could improve the prognosis for ALS patients. Other important pathways identified in our study were the activation of immune response, extracellular matrix composition, mitochondrial function, and RNA processing.

In parallel, we have analyzed four different mouse models, bearing the most common ALS-causing mutations. The results from human analyses were corroborated in ALS mouse models, which exhibited similar molecular patterns, and partially resembled the human subclusters revealed through the analysis of human brain tissue. This cross-species validation strengthens the relevance of the identified subclusters and pathways as potential therapeutic targets.

The findings summarized here were validated across multiple datasets, reinforcing the significance of the identified molecular sub clusters and pathways. This validation suggests that future ALS research should consider these frequently reported molecular differences, and focus on developing personalized medicine approaches, tailored to specific patient subgroups.

We hope that this clarifies the points put forward by the reviewer, as well as the intent and scope of the present manuscript.

2. The authors employed DESeq2 for identifying differentially expressed genes (DEGs) and miRNAs between experimental conditions (ALS vs. controls). While DESeq2 is a robust tool for differential expression analysis, it has limitations, particularly in handling low-count genes and in estimating dispersion in small sample sizes.

I would suggest the authors perform DEGs using the limma/voom approach. Limma, with its voom transformation, can provide better performance in terms of handling heteroscedasticity and is particularly effective for RNA-Seq data with varied library sizes. It also offers more accurate estimation of mean-variance relationships, which can improve the detection of true DEGs.

Additionally, using limma/voom can complement DESeq2 results and provide a more comprehensive analysis by cross-validating the findings. This dual approach would enhance the robustness and reliability of the differential expression analysis, potentially uncovering more biologically relevant insights.

Answer: We thank the reviewer for these suggestions. While limma/voom have been adapted for RNA-Seq data, it is important to highlight that limma was originally developed for microarray data. The voom transformation referred by the reviewer allows limma to handle RNA-Seq data by converting raw counts into log2 counts per million (log-CPM). However, this additional layer of modeling may not always capture the unique characteristics of RNA-Seq data as effectively as DESeq2. Limma-voom was originally only

tested against the previous version, DESeq, and not against DESeq2. Furthermore, in a recent benchmark by Li et al. (2022) [PMID: 36112652] DESeq2 remains the recommended method for most use cases with higher sample sizes (>6).
DESeq2, on the other hand, implements independent filtering and shrinkage estimation of dispersion, which makes it particularly robust in handling low-count genes — a common challenge in RNA-Seq data analysis. These features help minimize some false positives, ensuring more accurate results for low-count genes. Moreover, DESeq2's robust statistical framework, including its use of a negative binomial distribution, accounts for variability between experimental conditions. This makes DESeq2 especially well-suited for studies with expected variability, such as those comparing ALS patients to controls.
As requested by the reviewer, we have performed comparative analyses for both methods and the human samples and found that, in general, DESeq2 detected more differentially expressed genes than limma/voom - especially for males, which encompassed the most changes captured in our datasets (Venn diagrams). This is also the case for our proteomics samples (379 DEPs in males vs 251 DEPs in females) where we didn't use DESeq2 but limma (without voom) for our analysis.
While it can be valuable to use limma/voom as a complementary approach to validate DESeq2 findings, in our view, DESeq2 should remain the primary method due to its direct modeling of RNA-Seq data, and its handling of biological variability, low-count genes, the high detection rate for exploratory research and its consistency with the proteomics data. Employing both methods could enhance the robustness of the analysis, but DESeq2's approach is likely to yield more comprehensive primary results, particularly in our context.

3. The authors should provide a detailed description of how they adjusted for potential confounding variables in their DEGs and proteomics analyses analysis. Specifically, it is important to describe the methods used to account for batch effects, principal components of gene/protein expression, and the effects of age and sex on their analyses. Proper adjustment for these variables is crucial for ensuring the validity and reliability of the results.

Answer: We used PCA and a sample distance heatmap to check for batch effects in our transcriptomics and proteomics analyses. We could not detect any batch effects in our proteomics and transcriptomics data and exemplify this in the transcriptomics data below. We specifically investigated batch effects related to the factors brain bank, sex, case/control condition, as well as age at death. However, we identified marked sex-related differences (Figure 1b of Caldi Gomes, Hänzelmann et al., 2024; shown below) and therefore, all analyses were performed separately for each sex.

For the other factors, we found no evidence that they influenced the results (Supplementary Figure 1 of Caldi Gomes, Hänzelmann et al., 2024; shown below). For instance, if batch effects had been present, they would likely have caused distinct clustering or separation of samples. However, our analysis showed no such patterns, indicating that these factors did not introduce systematic bias to our data.

Furthermore, we could not detect any influence of the post-mortem interval on the expression of known neuronal cell death markers, indicating measurable influence by the sample handling (Supplementary Figure 4a of Caldi Gomes, Hänzelmann et al., 2024; shown below).
We now discuss this in a newly added section of the manuscript and added Figure 13 showing all known factors in the PCA representation for the transcriptomic (A) and proteomics (B) data.

Minor comments:
1. The authors should describe whether their omics data consist of single-end or paired-end reads.

Answer: Single-end reads. We made sure that this information is more explicitly stated in the methods.

2. The color scheme in Figure 6 makes it difficult to differentiate between cases and controls, particularly male-ctrl and male-als. I recommend that the authors revise the color scheme to improve contrast and distinguishability.

Answer: We thank the reviewer for the comment and agree with the color choice change. We resorted to a color-blind-friendly palette now and adjusted the respective figures.

--
Please also take a moment to check our website at https://www.editorialmanager.com/giga/l.asp?i=191766&l=V4LNH38F for any additional comments that were saved as attachments. Please note that as GigaScience has a policy of open peer review, you will be able to see the names of the reviewers.

No additional files for our submission.
