# Supplementary material for: A dataset profiling the multiomic landscape of the prefrontal cortex in amyotrophic lateral sclerosis
Source: Gigascience. 2024 Dec 18;13:giae100. doi: 10.1093/gigascience/giae100 (PMC11653894; doi:10.1093/gigascience/giae100)
Supplement: giae100_Reviewer_1_Report_Original_Submission — Adam Trautwig -- 7/23/2024 [file giae100_reviewer_1_report_original_submission.pdf]

Reviewer Report

Title: A Data Set Profiling the Multi-omic Landscape of the Prefrontal Cortex in Amyotrophic Lateral Sclerosis

Version: Original Submission **Date:** 7/23/2024

Reviewer name: Adam Trautwig

Reviewer Comments to Author:

This "Data Note" appears to be a well-written introduction to the dataset that was used to write citation 12 (i.e. https://doi.org/10.1038/s41467-024-49196-y). In order to evaluate this Data Note I retrieved the journals stated purpose for such article types and have included the pertinent details below:One of the aims of a Data Note is to incentivize and more rapidly release data before subsequent detailed analysis has been carried out. We do also publish Data Notes in coordination with or after the publication of an analysis paper, but we expect the Data Note to add value, especially in cases where the analysis paper has already been published. Data Notes should include significant additional detail that might not have been appropriate in the research paper, including information on data collection, detailed data validation, and information on exactly how these data can be re-usedAs the article has been published and the data made available, I would like to see the second part of the paragraph, an inclusion of significant additional detail, before publication, if that is what the authors and editor decide. I had a few questions related to the Data Note on its own that I would like to see addressed.1) I applaud the authors focus on sporadic ALS. As the authors point out, a subset of sALS patients harbor disease causing mutations citing references 2 and 3. Did you find any mutations associated with these cases? If so which and where? I'm also a little uncertain as to why the mouse models are the best method of interrogating the differences between sALS and gALS. Please expand on the utility of these specific mouse models and why you chose these models over gALS cases from the 4 brain banks.2) I am also a little dubious as to the justification of PFC as having signs of early alterations that are absent in other brain regions post mortem. I would like to see additional evidence that the authors may not have had space to include in the original publication.3) In the data preparation please include the number of files, fractionation, number of peptides, and total number of proteins and or transcripts.4) I would like to see additional searches for modifications to round out the inclusion of additional information about this dataset (for proteomics) that may not have fit in the original paper.Well done getting this information together, it was clearly a lot of work. Following major revisions I would be interested in seeing this note again.

Level of Interest

Please indicate how interesting you found the manuscript: Choose an item.

Quality of Written English

Please indicate the quality of language in the manuscript: Choose an item.

Declaration of Competing Interests

Please complete a declaration of competing interests, considering the following questions:

- Have you in the past five years received reimbursements, fees, funding, or salary from an organisation that may in any way gain or lose financially from the publication of this manuscript, either now or in the future?
- Do you hold any stocks or shares in an organisation that may in any way gain or lose financially from the publication of this manuscript, either now or in the future?
- Do you hold or are you currently applying for any patents relating to the content of the manuscript?
- Have you received reimbursements, fees, funding, or salary from an organization that holds or has applied for patents relating to the content of the manuscript?
- Do you have any other financial competing interests?
- Do you have any non-financial competing interests in relation to this paper?

If you can answer no to all of the above, write 'I declare that I have no competing interests' below. If your reply is yes to any, please give details below.

I declare that I have no competing interests

I agree to the open peer review policy of the journal. I understand that my name will be included on my report to the authors and, if the manuscript is accepted for publication, my named report including any attachments I upload will be posted on the website along with the authors' responses. I agree for my report to be made available under an Open Access Creative Commons CC-BY license (http://creativecommons.org/licenses/by/4.0/). I understand that any comments which I do not wish to be included in my named report can be included as confidential comments to the editors, which will not be published.

Choose an item.

To further support our reviewers, we have joined with Publons, where you can gain additional credit to further highlight your hard work (see: https://publons.com/journal/530/gigascience). On publication of this paper, your review will be automatically added to Publons, you can then choose whether or not to claim your Publons credit. I understand this statement.

Yes Choose an item.
