# Supplementary material for: A dataset profiling the multiomic landscape of the prefrontal cortex in amyotrophic lateral sclerosis
Source: Gigascience. 2024 Dec 18;13:giae100. doi: 10.1093/gigascience/giae100 (PMC11653894; doi:10.1093/gigascience/giae100)
Supplement: giae100_Reviewer_2_Report_Original_Submission — Lu Zeng -- 8/5/2024 [file giae100_reviewer_2_report_original_submission.pdf]

Reviewer Report

Title: A Data Set Profiling the Multi-omic Landscape of the Prefrontal Cortex in Amyotrophic Lateral Sclerosis

Version: Original Submission **Date:** 8/5/2024

Reviewer name: Lu Zeng

Reviewer Comments to Author:

Major comments:The authors have presented a useful omics resource for ALS studies, which could be used to identify biomarkers and drug targets for ALS treatments,. However, the following points should be addressed.1. Although the authors have described differential expression and enrichment analyses, as well as proteomics data analysis in their methods section, they did not report any of these results in the manuscript. Including these findings is crucial for providing a comprehensive understanding of the data and its implications. The absence of these results limits the ability of readers to fully assess the scope and impact of the analyses performed. I recommend that the authors include detailed results from these analyses to enhance the manuscript's completeness and clarity.2. The authors employed DESeq2 for identifying differentially expressed genes (DEGs) and miRNAs between experimental conditions (ALS vs. controls). While DESeq2 is a robust tool for differential expression analysis, it has limitations, particularly in handling low-count genes and in estimating dispersion in small sample sizes.I would suggest the authors perform DEGs using the limma/voom approach. Limma, with its voom transformation, can provide better performance in terms of handling heteroscedasticity and is particularly effective for RNA-Seq data with varied library sizes. It also offers more accurate estimation of mean-variance relationships, which can improve the detection of true DEGs.Additionally, using limma/voom can complement DESeq2 results and provide a more comprehensive analysis by cross-validating the findings. This dual approach would enhance the robustness and reliability of the differential expression analysis, potentially uncovering more biologically relevant insights.3. The authors should provide a detailed description of how they adjusted for potential confounding variables in their DEGs and proteomics analyses analysis. Specifically, it is important to describe the methods used to account for batch effects, principal components of gene/protein expression, and the effects of age and sex on their analyses. Proper adjustment for these variables is crucial for ensuring the validity and reliability of the results.Minor comments:1. The authors should describe whether their omics data consist of single-end or paired-end reads.2. The color scheme in Figure 6 makes it difficult to differentiate between cases and controls, particularly male-ctrl and male-als. I recommend that the authors revise the color scheme to improve contrast and distinguishability.

Level of Interest

Please indicate how interesting you found the manuscript: Choose an item.

Quality of Written English

Please indicate the quality of language in the manuscript: Choose an item.

Declaration of Competing Interests

Please complete a declaration of competing interests, considering the following questions:

- Have you in the past five years received reimbursements, fees, funding, or salary from an organisation that may in any way gain or lose financially from the publication of this manuscript, either now or in the future?
- Do you hold any stocks or shares in an organisation that may in any way gain or lose financially from the publication of this manuscript, either now or in the future?
- Do you hold or are you currently applying for any patents relating to the content of the manuscript?
- Have you received reimbursements, fees, funding, or salary from an organization that holds or has applied for patents relating to the content of the manuscript?
- Do you have any other financial competing interests?
- Do you have any non-financial competing interests in relation to this paper?

If you can answer no to all of the above, write 'I declare that I have no competing interests' below. If your reply is yes to any, please give details below.

I declare that I have no competing interests

I agree to the open peer review policy of the journal. I understand that my name will be included on my report to the authors and, if the manuscript is accepted for publication, my named report including any attachments I upload will be posted on the website along with the authors' responses. I agree for my report to be made available under an Open Access Creative Commons CC-BY license (http://creativecommons.org/licenses/by/4.0/). I understand that any comments which I do not wish to be included in my named report can be included as confidential comments to the editors, which will not be published.

Choose an item.

To further support our reviewers, we have joined with Publons, where you can gain additional credit to further highlight your hard work (see: https://publons.com/journal/530/gigascience). On publication of this paper, your review will be automatically added to Publons, you can then choose whether or not to claim your Publons credit. I understand this statement.

Yes Choose an item.
